# Cysteine-rich with EGF-like domains 2 (CRELD2) is an endoplasmic reticulum stress-inducible angiogenic growth factor promoting ischemic heart repair

**DOI:** 10.1038/s44161-023-00411-x

**Published:** 2024-01-17

**Authors:** Xuekun Wu, Linqun Zheng, Marc R. Reboll, Lillian F. Hyde, Elvira Mass, Hans W. Niessen, Maike Kosanke, Andreas Pich, Evangelos Giannitsis, Jochen Tillmanns, Johann Bauersachs, Joerg Heineke, Yong Wang, Mortimer Korf-Klingebiel, Felix Polten, Kai C. Wollert

**Affiliations:** 1 Division of Molecular and Translational Cardiology, Hans Borst Center for Heart and Stem Cell Research, Hannover Medical School, Hannover, Germany; 2 Department of Cardiology and Angiology, Hannover Medical School, Hannover, Germany; 3 Developmental Biology of the Immune System, Life & Medical Sciences Institute, University of Bonn, Bonn, Germany; 4 Department of Pathology and Department of Cardiac Surgery, Institute for Cardiovascular Research, Amsterdam University Medical Center, Amsterdam, The Netherlands; 5 Research Core Unit Genomics, Hannover Medical School, Hannover, Germany; 6 Core Unit Proteomics and Institute of Toxicology, Hannover Medical School, Hannover, Germany; 7 Department of Medicine III, Heidelberg University, Heidelberg, Germany; 8 Department of Cardiovascular Physiology, European Center for Angioscience, Medical Faculty Mannheim, Heidelberg University, Mannheim, Germany; 9 Present Address: Stanford University School of Medicine, Stanford, CA USA; 10 Present Address: Department of Cardiology, Shanghai General Hospital, Shanghai, China

**Keywords:** Angiogenesis, Extracellular signalling molecules, Cardiovascular diseases

## Abstract

Tissue repair after myocardial infarction (MI) is guided by autocrine and paracrine-acting proteins. Deciphering these signals and their upstream triggers is essential when considering infarct healing as a therapeutic target. Here we perform a bioinformatic secretome analysis in mouse cardiac endothelial cells and identify cysteine-rich with EGF-like domains 2 (CRELD2), an endoplasmic reticulum stress-inducible protein with poorly characterized function. CRELD2 was abundantly expressed and secreted in the heart after MI in mice and patients. *Creld2*-deficient mice and wild-type mice treated with a CRELD2-neutralizing antibody showed impaired de novo microvessel formation in the infarct border zone and developed severe postinfarction heart failure. CRELD2 protein therapy, conversely, improved heart function after MI. Exposing human coronary artery endothelial cells to recombinant CRELD2 induced angiogenesis, associated with a distinct phosphoproteome signature. These findings identify CRELD2 as an angiogenic growth factor and unravel a link between endoplasmic reticulum stress and ischemic tissue repair.

## Abstract (web summary)

Wu et al. profile the secretome of cardiac endothelial cells exposed to endoplasmic reticulum (ER) stress during acute myocardial infarction and identify CRELD2 as an angiogenic growth factor supporting infarct repair.

## Main

Acute myocardial infarction (MI) is a medical emergency caused by coronary artery thrombosis and occlusion, leading to progressive cell death in the hypoperfused territory^1^. Adult mammalian cardiomyocytes have limited regenerative capacity, and the infarcted heart therefore heals by scar formation^2^. Loss of contractile myocardium and scarring may cause deleterious changes of left ventricular (LV) tissue architecture resulting in heart failure^3^.

Wound healing after MI involves interactions among multiple cell types, which occur to a large extent through secreted proteins and their cognate receptors. Endothelial cells (ECs) function as a central hub in these ligand–receptor networks^4^. Reacting to angiogenic cues in the infarct wound, ECs mount a vigorous angiogenic response that mitigates scarring and worsening of heart function and may represent a therapeutic target^5–7^. In other disease contexts, ECs are known to also supply specific sets of growth factors promoting tissue adaptation and repair^8^. In the setting of acute MI, such angiocrine signals and their upstream triggers remain poorly defined^9^.

Many growth factors are released from cells via the classical endoplasmic reticulum (ER)–Golgi secretory pathway. Along this route, about one-third of all cellular proteins are synthesized, folded, posttranslationally modified and delivered to their final intra- or extracellular destinations^10^. These essential chores are performed by proteins that are retained in the ER–Golgi lumen by their C-terminal ER retention sequence (ERS) interacting with KDEL (lysine, aspartic acid, glutamic acid, leucine) receptors^11,12^. When physiological demands or pathological insults overwhelm the ER–Golgi system, misfolded proteins accumulate in the ER lumen, a condition referred to as ER stress. Three ER transmembrane receptors, activating transcription factor 6 (ATF6), inositol-requiring enzyme 1 alpha (IRE1α) and PKR-like ER kinase (PERK), sense misfolded proteins through their ER luminal domains and activate signal transduction pathways, collectively termed the unfolded protein response (UPR)^13^. Aiming to restore ER homeostasis, the UPR reduces the ER protein-folding load, by down-tuning translation and promoting messenger RNA (mRNA) decay, and increases the ER protein-folding capacity, by transcriptionally upregulating the ER luminal protein-folding machinery^13^. Although overall protein trafficking through the classical secretory pathway is impaired during ER stress, secretion of a select set of proteins, including many growth factors, may increase owing to increased production or release from the ER^5,14–17^.

Ischemia–reperfusion injury, inflammation and the increased secretory demand in the infarct wound jeopardize ER homeostasis and provoke ER stress^18,19^. Hypothesizing that ER stress triggers angiocrine signaling after acute MI, we performed a bioinformatic secretome analysis in cardiac ECs exposed to ER stress. We thus identified a poorly characterized ER stress-inducible secreted protein, cysteine-rich with EGF-like domains 2 (CRELD2), promoting infarct repair and showing therapeutic potential.

## Results

### In silico secretome analysis

**Fig. 1:**
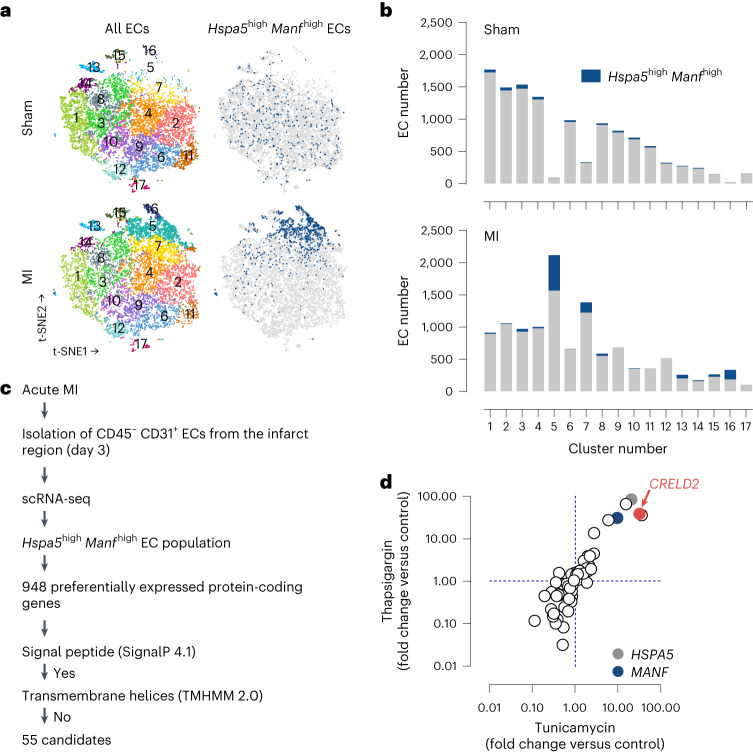
Secretome analysis. **a**, t-SNE plots of the scRNA-seq data depicting all cardiac ECs and *Hspa5*^high^ *Manf* ^high^ cardiac ECs (blue) 3 days after sham or MI surgery in wild-type mice. **b**, Total (gray and blue) and *Hspa5*^high^ *Manf* ^high^ (blue) EC numbers in each of the 17 EC clusters after sham or MI surgery. **c**, Experimental strategy to identify putatively secreted proteins preferentially expressed in the *Hspa5*^high^ *Manf* ^high^ EC population. **d**, mRNA expression (RT-qPCR, normalized to *GAPDH*; fold change versus unstimulated control) of 55 candidate genes in HCAECs stimulated for 20 h with thapsigargin (2 μmol l^−1^) or tunicamycin (2.5 µmol l^−1^). The vertical and horizontal dashed lines indicate no change versus control (ratio = 1). Source data

To identify previously uncharacterized proteins released from cardiac ECs under ER stress conditions after MI, we subjected wild-type mice to transient coronary ligation for 1 h (ischemia) followed by reperfusion. This model simulates the situation in patients with acute MI receiving reperfusion therapy and was used throughout our study. Three days after reperfusion, we isolated ECs from the infarct region (infarct core and border zone) by fluorescence-activated cell sorting. We transcriptionally profiled 11,743 ECs by single-cell RNA sequencing (scRNA-seq) and defined an EC subpopulation strongly expressing *heat shock protein family A member 5* (*Hspa5*) and *mesencephalic astrocyte-derived neurotrophic factor* (*Manf*) (Fig. 1a), two canonical ER stress-inducible genes^20^ intensely expressed in the cardiomyocyte and noncardiomyocyte compartments in the infarcted heart^15,21^. While few *Hspa5*^high^ *Manf*^high^ ECs, scattered across several EC clusters, were detected under sham-operated baseline conditions (*n* = 372, 3.2% of all cells), the *Hspa5*^high^ *Manf* ^high^ EC population expanded and clustered together after MI (*n* = 1,100, 9.4%) (Fig. 1a,b). Pathway analysis indicated that genes related to cell survival, proliferation and migration were enriched, whereas genes related to apoptosis were downregulated in *Hspa5*^high^ *Manf* ^high^ ECs (Supplementary Table 1). Gene expression data were filtered for transcripts that were preferentially expressed in *Hspa5*^high^ *Manf* ^high^ ECs after MI and predicted to encode secreted proteins (Fig. 1c and Supplementary Table 2). In a subsequent screen in human coronary artery endothelial cells (HCAECs), we found one of these transcripts, *CRELD2*, to be strongly upregulated during ER stress imposed by thapsigargin (Tg) or tunicamycin (Tm) stimulation (Fig. 1d and Supplementary Table 2). *CRELD2* encodes a 36 kDa protein (CRELD2) with an N-terminal signal peptide and a noncanonical C-terminal ERS (REDL). As previously shown in (tumor) cell lines, CRELD2 is weakly expressed under basal conditions but strongly induced and secreted during ER stress^22,23^. CRELD2 has been detected in urine after Tm-induced renal injury in mice or perioperative kidney injury in patients, indicating that it may be secreted also in vivo^24^. The function of CRELD2 in the cardiovascular system was previously unknown.

### Simulated ischemia–reperfusion induces CRELD2 via UPR signaling

**Fig. 2:**
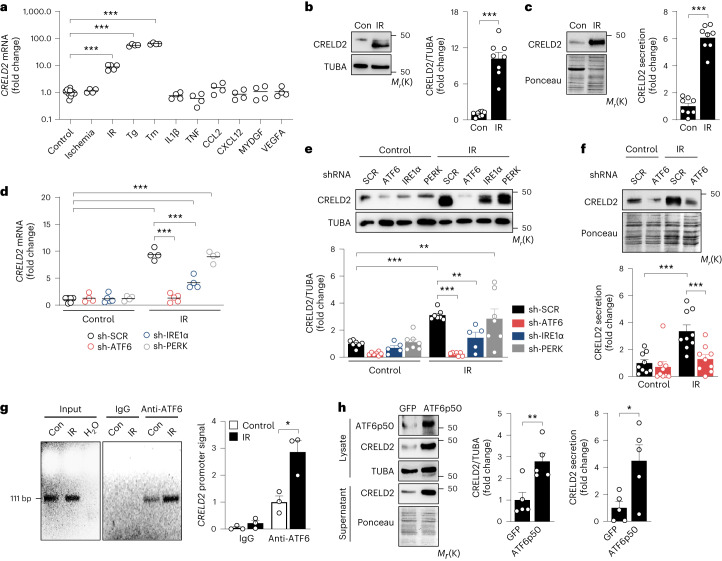
CRELD2 expression and secretion in ECs. **a**, *CRELD2* mRNA expression (RT-qPCR, normalized to *GAPDH*) in HCAECs exposed to simulated ischemia (20 h) or simulated ischemia (4 h) followed by reperfusion (16 h) (IR), or stimulated with Tg (2 μmol l^−1^), Tm (2.5 μmol l^−1^), IL1β (10 ng ml^−1^), TNF (5 ng ml^−1^), CCL2 (100 ng ml^−1^), CXCL12 (50 ng ml^−1^), mouse MYDGF (100 ng ml^−1^) or VEGFA (50 ng ml^−1^) for 20 h. There were 4–10 experiments conducted. Individual data points and mean values are shown. ***P < 0.001 (one-way ANOVA with Dunnett test). **b**,**c** HCAECs were cultured under control conditions (Con) or exposed to IR; exemplary immunoblots and summary data show CRELD2 expression in HCAEC lysates (normalized to alpha-tubulin (TUBA)) (**b**) and supernatants (Ponceau S staining used as loading control) (**c**). There were eight experiments conducted. ***P < 0.001 (two-sided independent-sample *t*-test). *M*_*r*_(K) denotes molecular mass in kDa. **d**–**f**, HCAECs were infected with lentiviruses encoding a scrambled (SCR) shRNA or shRNAs targeting ATF6, IRE1α or PERK. Cells were then cultured under control conditions or exposed to IR. **d**, *CRELD2* mRNA expression (RT-qPCR, normalized to *GAPDH*). There were four to six experiments conducted. Individual data points and mean values are shown. ***P < 0.001 (two-way ANOVA with Sidak test). **e**,**f**, Exemplary immunoblots and summary data showing CRELD2 expression in HCAEC lysates (**e**) and supernatants (**f**). There were five to nine experiments conducted. **P < 0.01; ***P < 0.001 (two-way ANOVA with Sidak test). **g**, Exemplary PCR analysis and summary data (three experiments) showing the chromatin immunoprecipitation signal of ATF6 binding to the *CRELD2* promoter region in HCAECs cultured under control conditions or exposed to IR. *P < 0.05 (two-sided independent-sample *t*-test). **h**, HUVECs were transfected with expression plasmids encoding the N-terminal fragment of ATF6 (ATF6p50) or GFP (used as a control). Exemplary immunoblots and summary data showing CRELD2 expression in cell lysates and supernatants. There were five experiments conducted. *P < 0.05; **P < 0.01 (two-sided independent-sample *t*-test). Unless otherwise stated, individual data points, mean values and s.e.m. are shown. Source data

**Extended Data Fig. 1:**
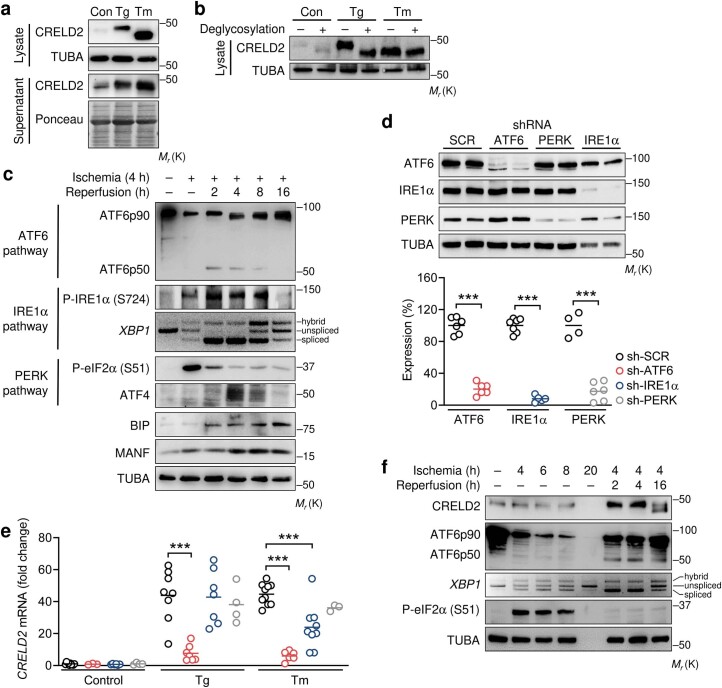
ER stress-related signaling in human coronary artery endothelial cells (HCAECs). (**a**, **b**) HCAECs were cultured under control conditions (Con) or stimulated with thapsigargin (Tg, 2 µmol/L) or tunicamycin (Tm, 2.5 µmol/L) for 20 hours. (**a**) Exemplary immunoblots (out of 4) showing CRELD2 and alpha-tubulin (TUBA) expression in cell lysates and CRELD2 expression in supernatants (Ponceau S staining used as loading control). (**b**) Treating protein extracts with deglycosidases before immunoblotting revealed that CRELD2 was glycosylated under control and Tg-stimulated conditions, but not after stimulation with the N-glycosylation-inhibitor Tm. Exemplary immunoblots (out of 3). (**c**) HCAECs were cultured under control conditions or exposed to simulated ischemia or simulated ischemia followed by reperfusion. Exemplary immunoblots (out of 3) show activating transcription factor 6 (ATF6; full length [p90] and N-terminal fragment [p50]), phosphorylated (S724)-inositol-requiring enzyme 1 alpha (P-IRE1α), phosphorylated (S51)-eukaryotic initiation factor 2 subunit alpha (P-eIF2α), activating transcription factor 4 (ATF4), endoplasmic reticulum chaperone BiP (BIP, which is encoded by *HSPA5*) and mesencephalic astrocyte-derived neurotrophic factor (MANF) expression (normalized to TUBA). *XBP1* mRNA splicing was assessed by RT-PCR. (**d, e**) HCAECs were infected with lentiviruses encoding a scrambled (SCR) short hairpin (sh)RNA or shRNAs targeting ATF6, IRE1α, or PERK. Individual data points and mean values are shown. (d) Exemplary immunoblots showing ATF6, IRE1α, and PERK expression (normalized to TUBA) and summary data 3 days after infection. 4–7 experiments. ***P < 0.001 (two-sided independent samples *t* tests). (e) Infected cells were cultured under control conditions or stimulated for 20 hours with Tg (2 μmol/L) or Tm (2.5 μmol/L) as indicated. 3–10 experiments. ***P < 0.001 (1-way ANOVA with Dunnett test). (**f**) HCAECs were cultured under control conditions or exposed to simulated ischemia or simulated ischemia followed by reperfusion. Exemplary immunoblots (out of 3) are shown. Note reduced protein content after 20 hours of ischemia. Source data

Aiming to define upstream inducers of CRELD2 in ECs, we found that exposing HCAECs to simulated ischemia–reperfusion (IR) enhanced *CRELD2* mRNA expression, whereas exposing the cells to simulated ischemia alone, or stimulating them with inflammatory cytokines, chemokines or angiogenic growth factors involved in infarct repair, did not (Fig. 2a). Simulated IR also induced CRELD2 protein expression (Fig. 2b) and secretion (Fig. 2c), as did treating the cells with Tg or Tm (Extended Data Fig. 1a,b). Simulated IR activated all three UPR pathways in HCAECs (Extended Data Fig. 1c), and we therefore explored whether IR induces CRELD2 expression via UPR signaling. To this end, we silenced each of the three UPR branches in HCAECs using short hairpin RNA (shRNA)-encoding lentiviral vectors (Extended Data Fig. 1d). ATF6 silencing prevented IR-induced increases in *CRELD2* mRNA expression (Fig. 2d) and CRELD2 protein expression (Fig. 2e) and secretion (Fig. 2f). Silencing IRE1α attenuated IR-induced CRELD2 expression to a lesser degree, whereas inhibiting PERK did not affect IR-induced CRELD2 expression (Fig. 2d,e). ATF6 silencing also exerted the strongest inhibitory effect on Tg or Tm induction of *CRELD2* mRNA abundance in HCAECs (Extended Data Fig. 1e). Consistent with these findings, chromatin immunoprecipitation documented increased ATF6 binding to the *CRELD2* promoter region in HCAECs exposed to simulated IR (Fig. 2g). Conversely, overexpressing the transcriptionally active N-terminal fragment of ATF6 (ATF6p50) increased CRELD2 protein expression and secretion in human umbilical vein ECs (HUVECs; Fig. 2h). Of note, simulated ischemia alone did not promote ATF6 activation and CRELD2 protein expression in HCAECs (Extended Data Fig. 1f).

### Increased CRELD2 expression and secretion after acute MI

**Fig. 3:**
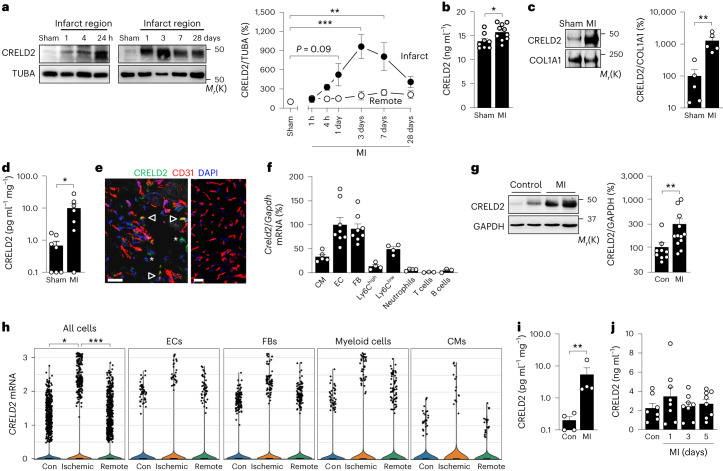
CRELD2 expression after MI. **a**, Exemplary immunoblots and summary data showing CRELD2 expression (normalized to TUBA) in the infarct region and remote (noninfarcted) region of the left ventricle after sham (28 days) or MI surgery. There were five to six mice per group. Data are presented as mean values ± s.e.m. **P < 0.01; ***P < 0.001 (one-way ANOVA with Dunnett test). **b**, CRELD2 plasma concentrations 3 days after sham surgery or MI. There were 8–10 mice per group. *P < 0.05 (two-sided independent-sample *t*-test). **c**, Exemplary immunoblots and summary data showing CRELD2 expression (normalized to collagen type I alpha 1 chain (COL1A1)) in the ECM of the infarct region 3 days after sham or MI surgery. There were five mice per group. **P < 0.01 (two-sided independent-sample *t*-test after logarithmic transformation). **d**, CRELD2 concentrations in supernatants of ex vivo heparinase-digested infarct regions 3 days after sham or MI surgery. There were seven mice per group. *P < 0.05 (two-sided independent-sample *t*-test after logarithmic transformation). **e**, Exemplary confocal immunofluorescence microscopy images taken from the infarct border zone 3 days after MI. Sections were stained with antibodies against CRELD2 and the endothelial marker CD31. The sections are from a wild-type mouse (left) and a *Creld2*-deficient mouse (right) (scale bars, 20 μm). CRELD2 was expressed by CD31^+^ (arrowheads) and CD31^–^ cells (asterisks). **f**, *Creld2* mRNA expression (RT-qPCR, normalized to *Gapdh*) in cardiomyocytes (CMs), ECs, fibroblasts (FBs), Ly6C^high^ monocytes, Ly6C^low^ monocytes or macrophages, neutrophils, T cells and B cells isolated from the infarct region 3 days after MI. There were three to eight mice per group. Expression in ECs was defined as 100%. **g**, Exemplary immunoblots and summary data showing CRELD2 expression (normalized to GAPDH) in LV tissue samples from 12 patients who had died of an acute MI and 9 patients who had died from noncardiac causes (Con). **P < 0.01 (two-sided independent-sample *t*-test after logarithmic transformation). **h**, Expression distribution of normalized nuclear *CRELD2* mRNA expression in all cell types (7,993 nuclei per group), ECs, FBs, myeloid cells and CMs in LV myocardial samples from nontransplanted donor hearts (Con) or in ischemic or remote (noninfarcted) LV myocardial samples from patients with acute MI. *P < 0.05; ***P < 0.001 (Wilcoxon rank sum test). **i**, CRELD2 concentrations in supernatants of heparinase-digested LV tissue samples from four patients who had died of an acute MI and four control patients. **P < 0.01 (two-sided independent-sample *t*-test after logarithmic transformation). **j**, CRELD2 plasma concentrations in seven apparently healthy control individuals and eight patients with acute MI (serially measured 1, 3 and 5 days after hospital admission). Unless otherwise stated, individual data points, mean values and s.e.m. are shown. Source data

Having identified CRELD2 in ECs from the infarcted mouse heart, we further explored its expression and cellular sources after acute MI. CRELD2 protein expression in the LV myocardium was low under baseline conditions but swiftly (within hours) and strongly upregulated in the infarct region after MI, attaining peak expression on day 3 (Fig. 3a). Increased cardiac CRELD2 expression was associated with only a slight increment in CRELD2 plasma concentrations (Fig. 3b). Instead, we found CRELD2 to be highly enriched in the extracellular matrix (ECM) of the infarct region (Fig. 3c). Heparan sulfate (HS) proteoglycans abound in the ECM of healing infarcts and provide binding sites for biologically active proteins, including growth factors and cytokines^25,26^. To assess, therefore, whether CRELD2 is attached to HS in the ECM, we digested infarct tissue with heparinase to depolymerize HS polysaccharide chains and release HS-bound protein ligands into the supernatant^25^. Indeed, ex vivo heparinase digestion released CRELD2 from infarcted tissue samples, indicating that secreted CRELD2 associates with HS in the ECM (Fig. 3d). Confocal immunofluorescence microscopy of the infarct region identified 46 ± 5% of the CRELD2-expressing cells as CD31^+^ ECs (data from five mice), indicating that cells other than ECs also express CRELD2 after MI (Fig. 3e). Consistently, reverse transcription-quantitative polymerase chain reaction (RT-qPCR) analysis defined ECs, fibroblasts, Ly6C^low^ monocytes or macrophages, and cardiomyocytes as the predominant *Creld2* mRNA-expressing cell types in the infarct region (Fig. 3f). Likewise, CRELD2 was abundantly expressed in myocardial tissue specimens from patients who had died of an acute MI (Fig. 3g). In a single nucleus RNA sequencing (snRNA-seq) database^27^, cells strongly expressing *CRELD2* mRNA in the infarcted human heart were found among the EC, fibroblast, myeloid cell and cardiomyocyte populations (Fig. 3h). Like in mice, secreted CRELD2 remained associated with HS in the ECM of the infarcted human heart (Fig. 3i) with no noticeable rise in CRELD2 plasma concentrations (Fig. 3j).

### CRELD2 enhances angiogenesis and heart function after MI

**Fig. 4:**
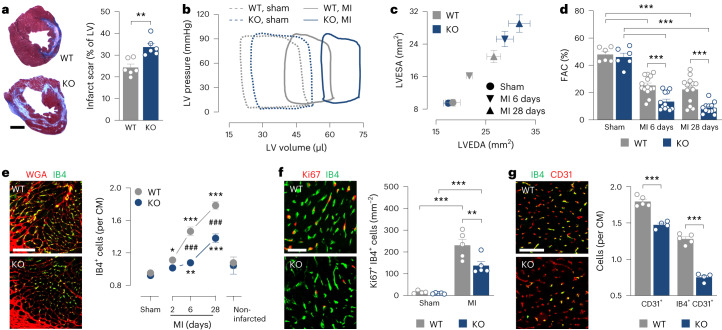
CRELD2 attenuates adverse LV remodeling after MI. *Creld2*-deficient (knockout (KO)) mice and their wild-type (WT) littermates underwent sham or MI surgery. **a**, Scar size 28 days after MI. Exemplary tissue sections (scale bar, 1 mm) and summary data from six mice per group. **P < 0.01 (two-sided independent-sample *t*-test). **b**, Exemplary LV pressure–volume loops recorded at 28 days. **c**, LV end-diastolic area (LVEDA) and LV end-systolic area (LVESA) as determined by echocardiography 28 days after sham surgery or 6 and 28 days after MI. There were 6–14 mice per group. Data are presented as mean values ± s.e.m. **d**, Fractional area change (FAC) as determined by echocardiography. Same mice as in **c**. ***P < 0.001 (one-way ANOVA with Dunnett test, MI versus same-genotype sham; two-sided independent-sample *t*-test, KO versus WT). **e**, Fluorescent images showing IB4^+^ ECs in the infarct border zone 28 days after MI. ECM and CM borders are highlighted by wheat germ agglutinin (WGA) staining (scale bar, 100 μm). Summary data from 5–10 mice per group (sham, 28 days; infarct border, indicated time points; noninfarcted region, 28 days). Data are presented as mean values ± s.e.m. *P < 0.05; **P < 0.01; ***P < 0.001 MI versus same-genotype sham (one-way ANOVA with Dunnett test); ^###^*P* < 0.001 KO versus WT (two-sided independent-sample *t*-test). **f**, Fluorescent images showing Ki67^+^ IB4^+^ proliferating ECs in the infarct border zone 6 days after MI (scale bar, 50 μm) and summary data from four to five mice per group. **P < 0.01; ***P < 0.001 (two-way ANOVA with Sidak test). **g**, Mice were intravenously injected with fluorescently labeled IB4 to label perfused capillaries. CD31 immunostaining was used to visualize all capillaries (perfused and nonperfused). Fluorescence microscopy images from the infarct border zone 28 days after MI (scale bar, 50 μm). Summary data from four to five mice per group. ***P < 0.001 (two-sided independent-sample *t*-test). Unless otherwise stated, individual data points, mean values and s.e.m. are shown. Source data

We next explored the function of CRELD2 after MI by subjecting *Creld2*-deficient mice and their wild-type littermates to MI surgery. *Creld2*-deficient mice breed normally and show no apparent phenotype at a young age^28^. Indeed, these mice had a normal heart size and preserved LV function under sham-operated baseline conditions (Supplementary Table 3). After MI, *Creld2*-deficient mice developed bigger infarct scars than the wild-type controls (Fig. 4a), with more pronounced LV dilatation and systolic dysfunction, as shown by microcatheterization (Fig. 4b and Supplementary Table 3) and serial echocardiography (Fig. 4c,d). Exploring the underlying causes of this detrimental response, we found that the formation of new capillaries in the infarct border zone during the first week after MI was impaired in *Creld2*-deficient mice (Fig. 4e). Impaired angiogenesis in *Creld2*-deficient mice was related to locally reduced EC proliferation (Fig. 4f) and resulted in a lower number of perfused capillaries in the infarct border zone (Fig. 4g). Myocardial capillarization under baseline conditions or remote from the infarct region was preserved in *Creld2*-deficient mice (Fig. 4e), indicating that CRELD2 specifically drives angiogenesis after MI.

### CRELD2 is an angiogenic growth factor

**Fig. 5:**
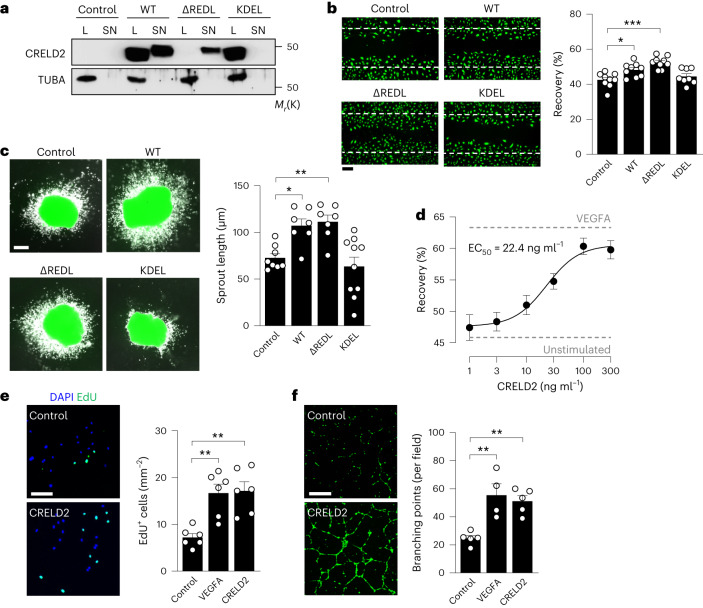
CRELD2 promotes angiogenic effects. **a**, Exemplary immunoblots showing CRELD2 expression (normalized to TUBA) in HCAEC lysates (L) and conditioned supernatants (SN) 24 h after infection with adenoviruses encoding DsRed (control), wild-type CRELD2 (WT), CRELD2 lacking its ER retention signal (ΔREDL) or CRELD2 in which RDEL was replaced by KDEL (KDEL). **b**, Recovery 16 h after scratch injury of adenovirus-infected HCAEC monolayers. Exemplary fluorescence microscopy images after BCECF-AM staining; the positions of the initial scratches are indicated by dashed lines (scale bar, 250 μm). Summary data from eight to nine experiments. *P < 0.05; ***P < 0.001 (one-way ANOVA with Dunnett test). **c**, MI was induced in *Tie2*-GFP mice. After 3 days, tissue samples from the infarct region were explanted and infected with adenoviruses encoding DsRed (control), wild-type CRELD2, CRELD2-ΔREDL or CRELD2-KDEL. Exemplary fluorescence microscopy images (scale bar, 100 μm) and average sprout length after 3 days. There were 7–10 experiments. *P < 0.05; **P < 0.01 (one-way ANOVA with Dunnett test). **d**, Recovery after scratch injury of HCAEC monolayers stimulated for 16 h with various concentrations of recombinant CRELD2. Dashed lines indicate recovery in unstimulated cells and VEGFA (50 ng ml^−1^)-stimulated cells. There were 5–11 experiments. Data are presented as mean values ± s.e.m. EC_50_ denotes half maximal effective concentration as calculated by four-parameter logistic regression. **e**, EdU incorporation in HCAECs cultured for 24 h in the absence or presence of VEGFA (50 ng ml^−1^) or CRELD2 (100 ng ml^−1^). Exemplary fluorescence images (scale bar, 100 μm). Summary data are from six experiments. **P < 0.01 (one-way ANOVA with Dunnett test). **f**, HCAEC network formation after stimulation for 4 h with VEGFA (50 ng ml^−1^) or CRELD2 (100 ng ml^−1^). Exemplary images after BCECF-AM staining (scale bar, 500 μm). Summary data are from four to five experiments. **P < 0.01 (one-way ANOVA with Dunnett test). Unless otherwise stated, individual data points, mean values and s.e.m. are shown. Source data

While we found endothelial CRELD2 to be secreted in response to ER stress, CRELD2 may also function inside the ER, acting as a chaperone and protein disulfide isomerase in a context-dependent manner^29,30^. The angiogenic defect in *Creld2*-deficient mice may therefore relate to a loss of EC-intrinsic, intra-ER functions of CRELD2 or a lack of autocrine (CRELD2 released from ECs) or paracrine (CRELD2 released from nonendothelial cell types) support of EC expansion. To test these hypotheses, we generated adenoviruses encoding wild-type CRELD2, CRELD2 lacking its noncanonical ER retention signal (CRELD2-ΔREDL) or CRELD2 in which RDEL was replaced by the canonical ER retention signal KDEL (CRELD2-KDEL). Expressing these CRELD2 variants in HCAECs, we detected wild-type CRELD2 in the cell lysate and supernatant, CRELD2-ΔREDL solely in the supernatant and CRELD2-KDEL solely in the cell lysate (Fig. 5a). Expressing either wild-type CRELD2 or CRELD2-ΔREDL in HCAECs enhanced their migratory ability in a confluent monolayer scratch assay, whereas expressing CRELD2-KDEL did not (Fig. 5b). Supporting the notion that secreted (extracellular) CRELD2 promotes angiogenic effects, expressing wild-type CRELD2 or CRELD2-ΔREDL stimulated EC outgrowth from infarcted mouse heart explants, whereas expressing CRELD2-KDEL did not (Fig. 5c). In line with this conclusion, exposure to recombinant CRELD2 stimulated HCAEC migration in a dose-dependent manner (Fig. 5d) and enhanced HCAEC proliferation (Fig. 5e) and network formation (Fig. 5f).

### CRELD2 promotes angiogenesis through AMPK and AKT1

**Fig. 6:**
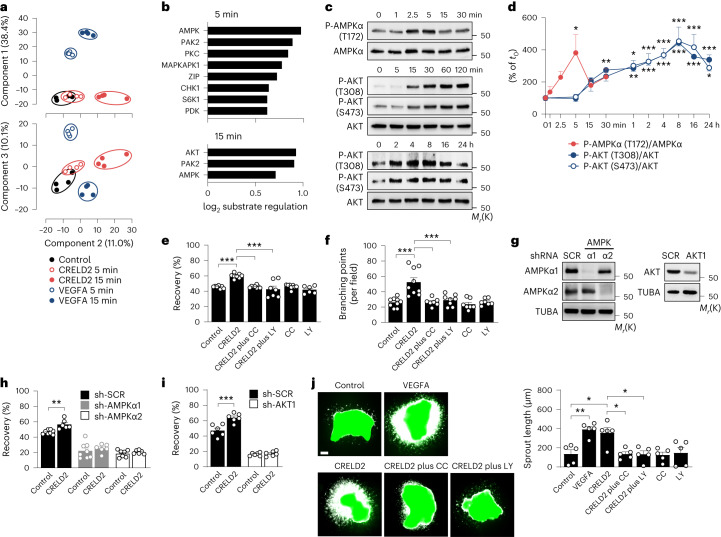
Phosphoproteome analysis identifies AMPK and AKT as signaling targets of CRELD2. **a**,**b**, Phosphoproteome analysis of HCAECs stimulated with CRELD2 (100 ng ml^−1^) or VEGFA (50 ng ml^−1^) for 5 or 15 min. **a**, Principal component analysis of the phosphoproteome datasets; there were four technical replicates per condition. **b**, Substrate-based kinase activity inference in CRELD2-stimulated versus unstimulated (control) cells. **c**,**d**, Exemplary immunoblots (**c**) and summary data (**d**) from three to nine experiments showing phosphorylated (P)-AMPKα (T172), AMPKα, P-AKT (T308), P-AKT (S473) and AKT expression in CRELD2-stimulated HCAECs. Data are presented as mean values ± s.e.m. *P < 0.05; **P < 0.01; ***P < 0.001 versus stimulation for 0 min (*t*_0_) (one-way ANOVA with Dunnett test). **e**,**f**, Recovery after scratch injury (**e**) and network formation (**f**) of HCAECs cultured for 16 h (scratch assay) or 4 h (networks) in the absence (control) or presence of CRELD2 (100 ng ml^−1^), compound C (CC, 5 μmol l^−1^) or LY294002 (LY, 5 μmol l^−1^). Summary data are from six to nine experiments. ***P < 0.001 (one-way ANOVA with Tukey test). **g**–**i**, HCAECs were infected with lentiviruses encoding a scrambled (SCR) shRNA or shRNAs targeting AMPKα1, AMPKα2 or AKT1. **g**, Exemplary immunoblots documenting shRNA-induced AMPKα1, AMPKα2 and AKT1 downregulation (−93 ± 5%, −82 ± 12% and −61 ± 6% versus sh-SCR, respectively; four to eight experiments). **h**,**i**, After lentiviral infection (SCR, AMPKα1 or AMPKα2 in **h**; SCR or AKT1 in **i**), cell monolayers were scratch-injured and cultured in the absence (control) or presence of CRELD2. Recovery was assessed after 16 h. There were six to eight experiments. **P < 0.01; ***P < 0.001 (two-way ANOVA with Sidak test). **j**, MI was induced in *Tie2*-GFP mice. After 3 days, tissue samples from the infarct region were explanted and cultured in the absence or presence of VEGFA (50 ng ml^−1^), CRELD2 (100 ng ml^−1^), CC (10 μmol l^−1^) or LY294002 (10 μmol l^−1^). Exemplary fluorescence microscopy images (scale bar, 200 μm) and average sprout length after 3 days. There were five experiments. *P < 0.05; **P < 0.01 (one-way ANOVA with Tukey test). Unless otherwise stated, individual data points, mean values and s.e.m. are shown. Source data

**Extended Data Fig. 2:**
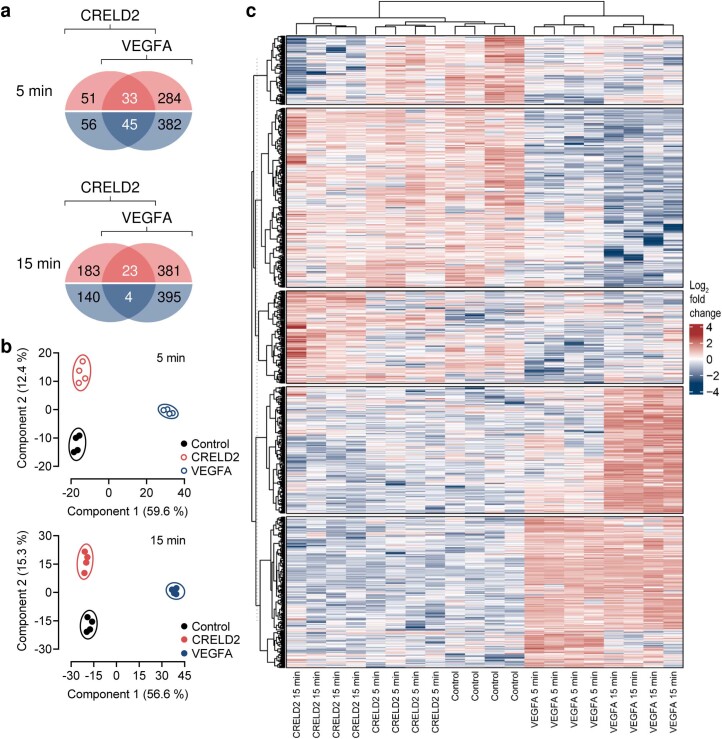
Venn diagram visualization, principal component analysis, and unsupervised hierarchical clustering of the phosphoproteome datasets. (**a**–**c**) Phosphoproteome analysis of human coronary artery endothelial cells stimulated for 5 or 15 min with CRELD2 (100 ng/mL) or vascular endothelial growth factor (VEGFA, 50 ng/mL); 4 technical replicates per time point and condition. (**a**) Venn diagrams showing the numbers of significantly up- (red) or downregulated (blue) phosphosites in CRELD2 or VEGFA-stimulated cells. (**b**) Principal component analysis. (**c**) Dendrograms depicting hierarchical clusters based on 499 significantly regulated phosphosites (Benjamini-Hochberg false discovery rate-corrected ANOVA, *P* < 0.05) using Ward’s method and Euclidian distance as a similarity metric. Source data

We used phosphoproteomics to identify the downstream signaling targets of CRELD2 in ECs. High-content liquid chromatography with tandem mass spectrometry (LC–MS/MS) data were collected from HCAECs cultured for 5 or 15 min in the absence or presence of recombinant CRELD2 or vascular endothelial growth factor A (VEGFA). Venn diagram visualization (Extended Data Fig. 2a), principal component analysis (Fig. 6a and Extended Data Fig. 2b) and unsupervised hierarchical clustering (Extended Data Fig. 2c) indicated that CRELD2 and VEGFA had distinct, albeit overlapping, phosphoproteome signatures. The numbers of phosphosites significantly upregulated or downregulated in CRELD2-stimulated cells were smaller than in VEGFA-stimulated cells (Extended Data Fig. 2a). Applying previous knowledge of kinase–substrate interactions to our phosphoproteome datasets, we predicted CRELD2 to activate several protein kinases, most prominently AMP-activated protein kinase (AMPK, at 5 min) and AKT serine/threonine kinase (AKT, at 15 min) (Fig. 6b). Immunoblotting experiments (Fig. 6c,d) supported these predictions and indicated that recombinant CRELD2 rapidly promotes AMP-activated protein kinase catalytic subunit alpha (AMPKα) phosphorylation at threonine 172 and, more slowly and sustainedly, AKT phosphorylation at threonine 308 and serine 473, phosphorylation events required for maximal AMPK and AKT activation^31,32^. The angiogenic effects of CRELD2 in HCAECs were curtailed by the AMPK-inhibiting compound C (ref. ^33^) (Fig. 6e,f), the phosphatidylinositol 3-kinase (PI3K) and PI3K-AKT pathway inhibitor LY294002 (ref. ^34^) (Fig. 6e,f), and shRNA-mediated AMPKα or AKT1 downregulation (Fig. 6g–i), indicating that CRELD2 functions through AMPKα and AKT. Affirming the importance of these signaling events in the context of an acute MI, CRELD2 stimulated EC outgrowth from infarcted mouse heart explants in a compound C- and LY294002-sensitive manner (Fig. 6j).

### Secreted CRELD2 enhances angiogenesis and heart function after MI

**Fig. 7:**
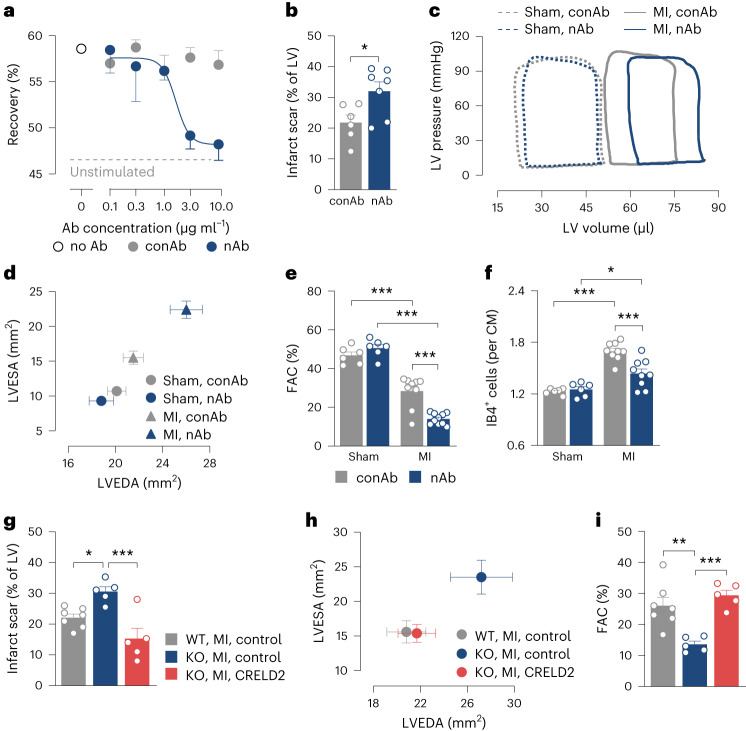
Secreted CRELD2 promotes angiogenesis and functional adaptation after MI. **a**, Recovery 16 h after scratch injury of HCAEC monolayers exposed to mouse CRELD2 (100 ng ml^−1^), either alone (no antibody (Ab)) or combined with increasing concentrations of a CRELD2-neutralizing Ab (nAb) or control Ab (conAb). The dashed line indicates recovery in unstimulated cells (no CRELD2). There were four to eight experiments. Data are presented as mean values ± s.e.m. **b**–**f**, Wild-type mice underwent sham or MI surgery and were injected with nAb or conAb (100 μg each) into the LV cavity at the time of reperfusion (matching time point in sham-operated mice). **b**, Scar size 28 days after MI. There were six or seven mice per group. *P < 0.05 (two-sided independent-sample *t*-test). **c**, Exemplary LV pressure–volume loops recorded at 28 days. **d**, LV end-diastolic area (LVEDA) and LV end-systolic area (LVESA) as determined by echocardiography at 28 days. There were 6–10 mice per group. Data are presented as mean values ± s.e.m. **e**, Fractional area change (FAC) as determined by echocardiography. Same mice as in **d**. ***P < 0.001 (two-way ANOVA with Sidak test). **f**, IB4^+^ ECs in the infarct border zone at 28 days (CM denotes cardiomyocyte). There were 6–10 mice per group. *P < 0.05; ***P < 0.001 (two-way ANOVA with Sidak test). **g**–**i**, MI was induced in *Creld2*-deficient (KO) mice and their WT littermates. In KO mice, CRELD2 (10 μg) was injected into the LV cavity at the time of reperfusion and then subcutaneously infused for 7 days (10 μg per day). WT and KO control mice were treated with diluent only (phosphate-buffered saline, bolus and infusion) only. **g**, Scar size at 28 days. There were five to seven mice per group. *P < 0.05; ***P < 0.001 (one-way ANOVA with Dunnett test). **h**, LVEDA and LVESA. There were five to seven mice per group. Data are presented as mean values ± s.e.m. **i**, FAC. Same mice as in **j**. **P < 0.01; ***P < 0.001 (one-way ANOVA with Dunnett test). Unless otherwise stated, individual data points, mean values and s.e.m. are shown. Source data

**Extended Data Fig. 3:**
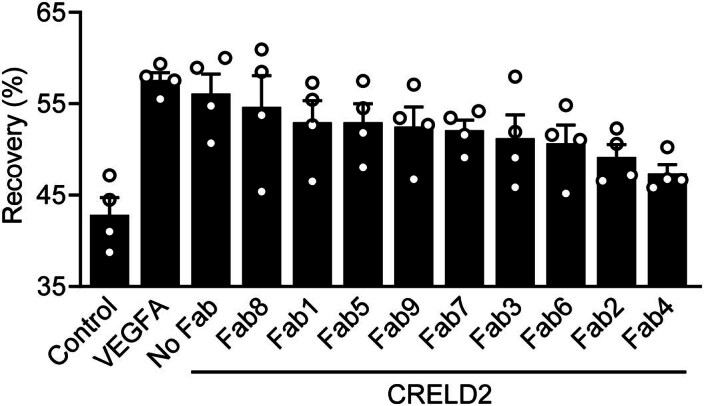
CRELD2-neutralizing activities of CRELD2-binding Fab fragments. Recovery 16 hours after scratch injury of human umbilical vein endothelial cell monolayers cultured in the absence (control) or presence of human VEGFA (50 ng/mL), mouse CRELD2 (100 ng/mL), and 9 Fab fragments (3 μg/mL) directed against mouse CRELD2. 4 experiments. Individual data points, mean values, and SEMs are shown. Source data

We generated a CRELD2-neutralizing antibody to assess the importance of secreted (extracellular) CRELD2 after MI. We therefore screened a fully synthetic human combinatorial phage display antibody library for Fab fragments binding to recombinant mouse CRELD2. Nine candidates were tested for their CRELD2-neutralizing activity in an HCAEC monolayer scratch assay (Extended Data Fig. 3). Fab fragment number 4 exhibited the greatest inhibitory activity and was converted into a human (Fab)–mouse (Fc) chimeric IgG1 neutralizing antibody (nAb) that dose-dependently inhibited recombinant mouse CRELD2-induced HCAEC migration (Fig. 7a). A chimeric IgG1 control antibody (conAb) showed no CRELD2-neutralizing activity (Fig. 7a). Neither antibody (10 μg ml^−1^) attenuated VEGFA (50 ng ml^−1^)-induced HCAEC migration (recovery after scratch injury: 64 ± 1% in nAb-treated cells versus 63 ± 2% in conAb-treated cells; 3 experiments). Wild-type mice treated with the nAb (100 μg into the LV cavity at the time of reperfusion) developed larger infarct scars (Fig. 7b) with more severe LV dilatation and systolic dysfunction (Fig. 7c–e and Supplementary Table 4) than wild-type mice treated with the conAb. Infarct border zone capillarization was impaired in mice treated with the nAb (Fig. 7f). Treating sham-operated mice with the nAb did not affect LV myocardial capillary density (Fig. 7f) and LV geometry or function (Fig. 7c–e and Supplementary Table 4). Treatment with the nAb therefore replicated the phenotype of *Creld2*-deficient mice, indicating that secreted CRELD2 promotes angiogenesis and supports functional adaptation after MI. In line with this conclusion, treatment with recombinant CRELD2 (bolus injection into the LV cavity at the time of reperfusion, followed by subcutaneous infusion for 7 days) rescued *Creld2*-deficient mice from increased scarring (Fig. 7g) and more severe adverse remodeling after MI (Fig. 7h,i).

### CRELD2 effects on nonendothelial cell types

**Fig. 8:**
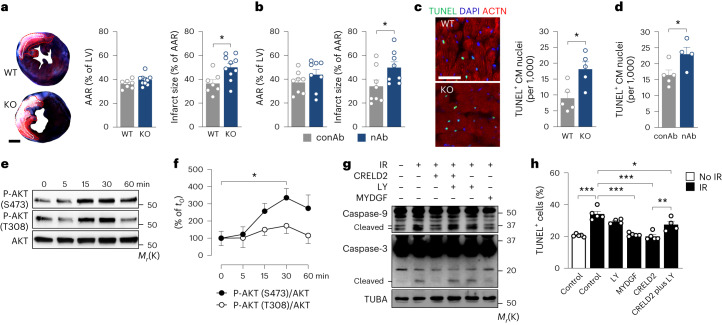
Secreted CRELD2 protects cardiomyocytes from IR injury. **a**, *Creld2*-deficient (KO) mice and their WT littermates underwent MI surgery. Area at risk (AAR, dashed red lines) and infarct size (dashed white lines) 24 h after MI. Exemplary Evans blue- and TTC-stained LV tissue sections (scale bar, 1 mm) and summary data from 7 to 10 mice per group. **b**, Wild-type mice underwent MI surgery and were injected with CRELD2-nAb or conAb (100 μg each) into the LV cavity at the time of reperfusion. AAR and infarct size 24 h after MI. There were eight mice per group. **c**, TUNEL^+^ CM nuclei in the infarct border zone 24 h after MI in WT and KO mice. Exemplary fluorescence images (ACTN, alpha-actinin; scale bar, 50 μm) and summary data from five mice per group. **d**, TUNEL^+^ CM nuclei in the infarct border zone 24 h after MI in conAb- or nAb-treated wild-type mice. There were four to five mice per group. In **a**–**d**, *P < 0.05 (two-sided independent-sample *t*-test). **e**,**f**, Exemplary immunoblots (**e**) and summary data (**f**) from three to four experiments showing phosphorylated (P)-AKT (S473), P-AKT (T308) and AKT expression in CRELD2 (100 ng ml^−1^)-stimulated NRCMs. Data are presented as mean values ± s.e.m. *P < 0.05 (one-way ANOVA with Dunnett test). **g**, Exemplary immunoblots (of three) showing caspase-9 (upper band, procaspase-9; lower bands, cleaved caspase-9), caspase-3 (upper band, procaspase-3; lower band, cleaved caspase-3) and TUBA expression in NRCMs exposed to simulated ischemia (3 h) followed by reperfusion (1 h; IR) in the absence or presence of CRELD2, MYDGF (100 ng ml^−1^) or LY294002 (LY, 10 μmol l^−1^). **h**, TUNEL assay in NRCMs exposed to IR in the absence (IR control) or presence of CRELD2, MYDGF or LY294002. There were four or five experiments. *P < 0.05; **P < 0.01; ***P < 0.001 (one-way ANOVA with Tukey test). Unless otherwise stated, individual data points, mean values and s.e.m. are shown. Source data

**Extended Data Fig. 4:**
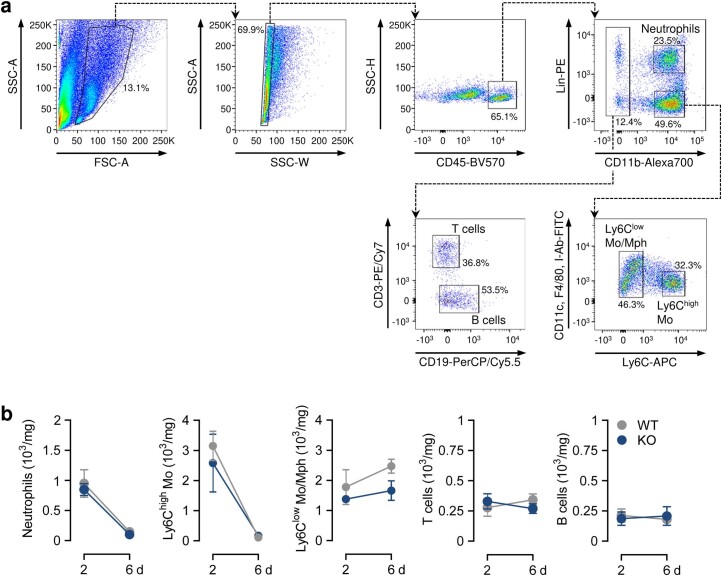
Inflammatory cell accumulation after myocardial infarction (MI). (**a**) Gating strategy. Mo denotes monocytes; Mph, macrophages. (**b**) Accumulation of neutrophils, Ly6C^high^ Mo, Ly6C^low^ Mo or Mph, T cells, and B cells in the infarct region 2 and 6 days after MI as assessed by flow cytometry in *Creld2*-deficient (knockout, KO) mice and their wild-type (WT) littermates. 4–5 mice per group. Data are presented as mean values ± SEM. Source data

**Extended Data Fig. 5:**
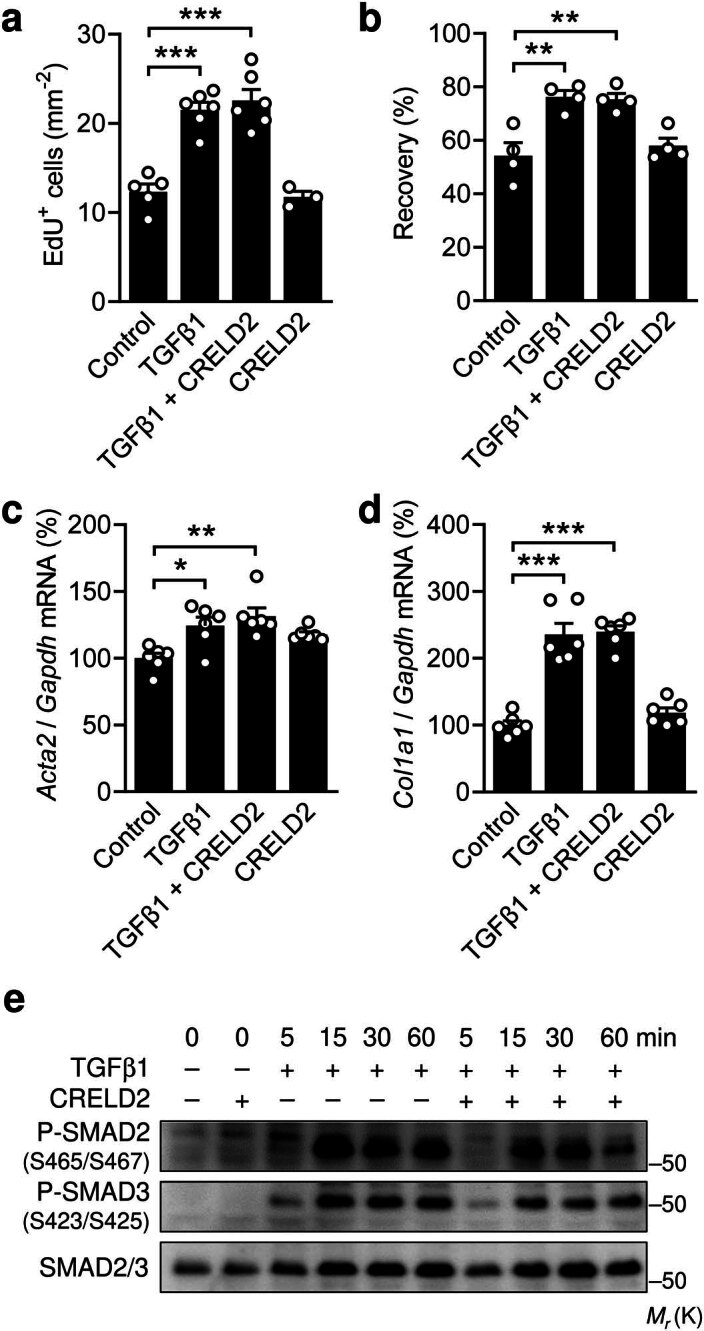
Lack of CRELD2 effects in murine embryonic fibroblasts. (**a**) EdU incorporation in mouse embryonic fibroblasts (MEFs) cultured for 24 hours in the absence or presence of transforming growth factor beta 1 (TGFβ1, 2 ng/mL) or CRELD2 (100 ng/mL). 3–6 experiments. (**b**) Recovery 16 hours after scratch injury of MEF monolayers in the absence or presence of TGFβ1 or CRELD2. 4 experiments. (**c**) *Smooth muscle actin alpha 2* (*Acta2*) and (**d**) *collagen type I alpha1* (*Col1a1*) mRNA expression (RT-qPCR, normalized to *glyceraldehyde-3-phosphate dehydrogenase* [*Gapdh*]) in MEFs cultured for 24 hours in the absence or presence of TGFβ1 or CRELD2. 6 experiments. (**a**–**d**) *P < 0.05, **P < 0.01, ***P < 0.001 (1-way ANOVA with Tukey test). Individual data points, mean values, and SEMs are shown. (**e**) Exemplary immunoblots (out of 3) showing phosphorylated (P)-SMAD2 (S465/S467), P-SMAD3 (S423/S425), and SMAD2 and SMAD3 expression in MEFs stimulated with TGFβ1 in the absence or presence of CRELD2 (added 30 min prior to TGFβ1). Source data

A properly coordinated angiogenic response is associated with smaller infarct scars, less severe adverse remodeling and better preserved heart function in animal models of acute MI^9^, suggesting that the angiogenic effects of CRELD2 are functionally important. Conceivably, secreted CRELD2 may target additional cell types in the infarcted heart. Exploring whether CRELD2 also acts on nonendothelial cells, we noticed that inflammatory cell accumulation in the infarct region was comparable between *Creld2*-deficient mice and their wild-type littermates (Extended Data Fig. 4a,b). We also found that recombinant CRELD2 does not affect proliferation (Extended Data Fig. 5a), migration (recovery after scratch injury; Extended Data Fig. 5b) and *smooth muscle actin alpha 2* and *collagen type I alpha 1* mRNA expression (RT-qPCR; Extended Data Fig. 5c,d) in unstimulated or transforming growth factor beta 1 (TGFβ1)-stimulated murine embryonic fibroblasts. CRELD2 also did not inhibit TGFβ1-induced mothers against decapentaplegic homolog 2 (SMAD2) and SMAD3 phosphorylation (Extended Data Fig. 5e), indicating that CRELD2 does not target fibroblasts directly. However, we observed that *Creld2*-deficient and CRELD2-nAb-treated mice had larger infarct sizes 24 h after reperfusion, despite a comparable area at risk (Fig. 8a,b). Larger infarct sizes in *Creld2*-deficient or nAb-treated mice were associated with increased numbers of TUNEL^+^ cardiomyocyte nuclei in the infarct border zone (Fig. 8c,d), raising the possibility that CRELD2 directly acts on cardiomyocytes. Indeed, recombinant CRELD2 promoted AKT phosphorylation at serine 473 in cultured neonatal rat cardiomyocytes (Fig. 8e,f) and protected the cells from simulated IR-induced caspase-3 and caspase-9 activation and apoptotic cell death in an LY294002-sensitive manner (Fig. 8g,h).

### CRELD2 protein therapy after MI in wild-type mice

**Extended Data Fig. 6:**
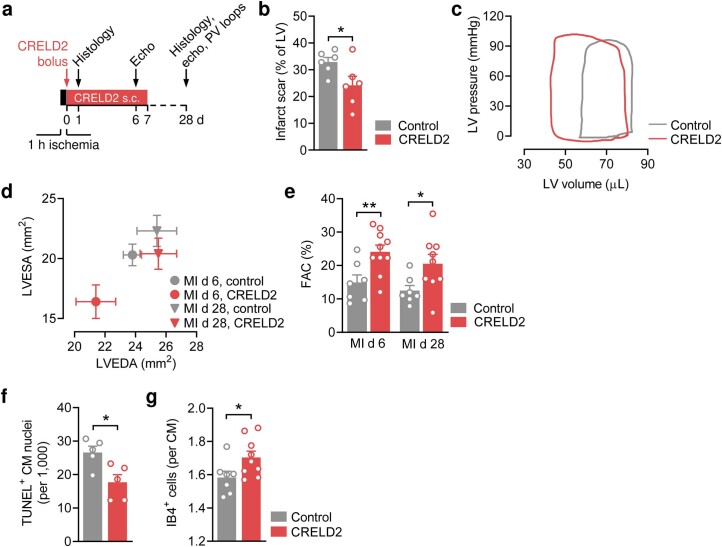
CRELD2 protein therapy after myocardial infarction (MI) in wild-type mice. (**a**) Dosing regimen. CRELD2 (10 μg) was injected into the left ventricular (LV) cavity at the time of reperfusion and then subcutaneously infused for 7 days (10 μg/day). Control mice were treated with diluent (phosphate-buffered saline) only (bolus and infusion). (**b**) Scar size at 28 days. 6 mice per group. *P < 0.05 (two-sided independent samples *t* test). (**c**) Exemplary LV pressure-volume loops recorded at 28 days. (**d**) LV end-diastolic area (LVEDA) and LV end-systolic area (LVESA) as determined by echocardiography at 6 or 28 days. 7–10 mice per group. Data are presented as mean values ± SEM. (**e**) Fractional area change (FAC) as determined by echocardiography. Same mice as in (**d**). *P < 0.05, **P < 0.01 (two-sided independent samples *t* test). (**f**) TUNEL^+^ cardiomyocyte (CM) nuclei in the infarct border zone 24 hours after MI. 5 mice per group. *P < 0.05 (two-sided independent samples *t* test). (**g**) Isolectin B4 (IB4)^+^ endothelial cells in the infarct border zone 28 days after MI. 7–9 mice per group. *P < 0.05 (two-sided independent samples *t* test). Unless otherwise stated, individual data points, mean values, and SEMs are shown. Source data

Considering the beneficial effects endogenous CRELD2 after acute MI, we tested the therapeutic potential of recombinant CRELD2 in infarcted wild-type mice. CRELD2 was bolus injected into the LV cavity at the time of reperfusion, followed by a subcutaneous infusion for 7 days (Extended Data Fig. 6a). CRELD2-treated mice developed smaller infarct scars (Extended Data Fig. 6b) with less severe LV dilatation and systolic dysfunction 6 and 28 days after reperfusion than control mice treated with diluent only (Extended Data Fig. 6c–e and Supplementary Table 5). These treatment benefits were associated with a reduced number of TUNEL^+^ cardiomyocytes in the infarct border zone 24 h after reperfusion (Extended Data Fig. 6f) and an increased capillarization of the infarct border zone at 28 days (Extended Data Fig. 6g).

## Discussion

After MI, the heart responds to numerous secreted proteins that are locally produced within the injured myocardium. Deciphering these signals and defining their upstream triggers are essential when considering infarct healing as a therapeutic target^35^. Here we have identified CRELD2 as an ER stress-inducible angiogenic growth factor promoting functional adaptation after MI.

We discovered CRELD2 in a bioinformatic secretome analysis in ECs from the infarct region of mice with reperfused acute MI. We focused our screen on ECs expressing *Hspa5* and *Manf*, two ER stress-inducible genes^20^ strongly expressed in the cardiomyocyte and noncardiomyocyte compartments in the infarcted myocardium^15,21^. Whereas few ECs expressed these marker genes under baseline conditions, the *Hspa5*- and *Manf*-expressing EC population expanded after MI. Functional annotation indicated that *Hspa5*- and *Manf*-expressing ECs are an expanding and activated cell population. *Creld2* mRNA was preferentially expressed in this EC population and strongly upregulated in HCAECs exposed to the ER stress inducers Tg or Tm. Searching for triggers that may induce endothelial CRELD2 in the setting of acute MI, we found simulated IR to greatly enhance CRELD2 mRNA and protein expression in HCAECs. IR, a known inducer of ER stress in the heart^18,19,36,37^, activated all three UPR branches in HCAECs in our study. While any of the three UPR pathways may induce CRELD2 in a context-dependent manner^20,22,38^, ATF6 emerged as the dominant driver of IR-induced endothelial CRELD2 expression.

CRELD2 contains a C-terminal ERS (REDL) that is conserved in mice and humans. In mouse models of genetic skeletal diseases in which misfolded structural proteins trigger ER stress and UPR signaling, CRELD2 has been proposed to function as a protein disulfide isomerase, acting in concert with other protein disulfide isomerases and chaperones inside the ER^29^. CRELD2 itself may also serve as a chaperone, assisting in the folding, maturation and transport of proteins along the secretory pathway^30^, thereby attenuating ER stress^28^. Compared with the canonical ERS (KDEL), REDL weakly interacts with KDEL receptors^12,39^, enabling CRELD2 to escape from the ER–Golgi system under ER stress conditions when multiple ER stress-induced, ERS-containing proteins compete for KDEL receptor binding sites^23,24,39^. Along these lines, our studies in HCAECs exposed to simulated IR or expressing CRELD2 ERS mutants revealed that augmenting the expression of CRELD2 increases its intracellular abundance and secretion.

CRELD2 was weakly expressed in the heart under baseline conditions but strongly upregulated after MI. Cells other than ECs also expressed CRELD2 in the infarcted myocardium. CRELD2 was secreted and retained by HS proteoglycans in the ECM, enabling it to serve autocrine or paracrine functions in the infarct region^25,26^. As shown in autopsy samples and an snRNA-seq dataset from patients with acute MI, this paradigm may be operative also in humans. Studying *Creld2*-deficient mice, we found that CRELD2 drives angiogenesis in the infarct border zone, thereby limiting scarring and adverse cardiac remodeling. Treating infarcted wild-type mice with a CRELD2-nAb replicated the phenotype of *Creld2*-deficient mice, indicating that extracellular (secreted) CRELD2 mediated these adaptive effects. In line with this conclusion, treating *Creld2*-deficient mice with recombinant CRELD2 rescued the animals from more severe scarring and remodeling after MI. Notably, infusing recombinant CRELD2 for 7 days after reperfusion also attenuated scarring and adverse remodeling in wild-type mice, indicating that CRELD2 protein therapy can enhance endogenous CRELD2 effects after MI and supporting the idea that infarct healing affords a time window of therapeutic opportunity wherein transient interventions can have a lasting impact on cardiac function^5–7,35^.

In HCAECs, CRELD2 promoted direct angiogenic effects that were associated with a distinct phosphoproteome signature. Substrate-based kinase activity inference predicted and loss-of-function studies confirmed that CRELD2 signals through AMPK and AKT, protein kinases that have previously been implicated in growth-factor-induced angiogenesis^40–43^. While we acknowledge the limitations of reductionist cell culture models in predicting the downstream signaling targets of CRELD2 in vivo, we posit that impaired myocardial capillarization in infarcted *Creld2*-deficient and CRELD2-nAb-treated mice pertains to direct angiogenic effects of secreted CRELD2.

Conceivably, extracellular CRELD2 may also act on nonendothelial cell types in the infarcted heart. While CRELD2 did not promote direct antifibrotic effects in cultured fibroblasts and did not affect inflammatory cell accumulation in the infarct region, it protected cardiomyocytes from IR-induced cell death. Indeed, *Creld2*-deficient and CRELD2-nAb-treated mice developed greater infarct sizes with more apoptotic cardiomyocytes in the infarct border zone. Consistent with direct cytoprotective effects, recombinant CRELD2 inhibited simulated IR-induced caspase activation and cell death in cultured cardiomyocytes in a PI3K-AKT pathway-dependent manner and inhibited cardiomyocyte apoptosis when therapeutically delivered to wild-type mice. We therefore propose that CRELD2 reduces scarring and adverse remodeling after MI by limiting infarct damage and by promoting angiogenesis and tissue repair.

While ER stress has been linked to cardiac pathologies in chronic heart failure^44,45^, our observations concur with previous reports showing that ER stress activates cardiomyocyte-protective signaling pathways after acute MI^15,21,36,37^. Our study further unravels a link between ER stress and postinfarction angiogenesis. This is reminiscent of findings from the cancer field showing that engagement of the UPR governs blood vessel formation in the tumor microenvironment by increasing angiogenic growth factor availability^46,47^. Similar to our observation that CRELD2 is retained in the ECM of the infarct region, CRELD2 has been detected in the tumor ECM where it may promote autocrine or paracrine effects^48^. Indeed, malignant tumor cells have been shown to communicate with tumor-residing stromal cells via CRELD2 (ref. ^38^).

In conclusion, we identify CRELD2 as a locally procured cardioprotective and angiogenic growth factor enhancing tissue perfusion and repair after IR injury. Our findings should stimulate further research into the role of secreted CRELD2 in other disease states, its therapeutic potential for heart repair and the identification of the putative receptor mediating its effects.

## Methods

The ethics committee of the VU University Medical Center in Amsterdam approved the use of human LV myocardial tissue samples. The ethics committees of Hannover Medical School and Heidelberg University approved the use of human plasma samples. All individuals donating plasma samples provided written informed consent. All animal procedures conformed to the guidelines from the EU directive 2010/63 on the protection of animals used for scientific purposes and were approved by the authorities in Hannover, Germany (Niedersächsisches Landesamt für Verbraucherschutz und Lebensmittelsicherheit).

### Small molecules

Tg (catalog number T9033), Tm (catalog number T7765), compound C (catalog number 171260) and LY294002 (catalog number 440202) were purchased from Sigma-Aldrich.

### Recombinant proteins

Human tumor necrosis factor (TNF; catalog number HZ-1014) and human interleukin 1 beta (IL1β; catalog number HZ-1164) were purchased from Proteintech; human CRELD2 (catalog number 3555-CR), mouse CRELD2 (catalog number 3686-CR), mouse TGFβ1 (catalog number 7666-MB), human VEGFA (catalog number 293-VE) and mouse VEGFA (catalog number 493-MV) from R&D Systems; and human C-C motif chemokine ligand 2 (CCL2; catalog number SRP3109) and human C-X-C motif chemokine ligand 12 (CXCL12; catalog number SRP3276) from Sigma-Aldrich. Mouse myeloid-derived growth factor (MYDGF) was produced as a His-tagged recombinant protein^49^. We confirmed the purity of human and mouse CRELD2 by untargeted MS/MS after trypsin digestion. Generated spectra were matched against the target protein and UniProt Knowledgebase (https://www.uniprot.org/). Purity was calculated based on unique (protein-specific) peptide intensities in relation to trace contaminants. Human CRELD2 (45 MS/MS spectra, 29 unique peptides) and mouse CRELD2 (66 MS/MS spectra, 27 unique peptides) were found to be 97.7% and 95.0% pure, respectively. Unless otherwise stated, we used the human proteins to treat human ECs and the mouse proteins to treat infarcted mice, mouse infarct explants, mouse embryonic fibroblasts and rat cardiomyocytes.

### Antibodies

Alpha-tubulin (clone EPR13478(B), catalog number ab185067, 1:3,500), ATF6 (clone EPR22690-84, catalog number ab227830, 1:1,000), phosphorylated (P) eukaryotic initiation factor 2 subunit alpha (Ser 51) (P-eIF2α, clone E90, catalog number ab32157, 1:1,000) and glyceraldehyde-3-phosphate dehydrogenase (GAPDH, clone mAbcam 9484, catalog number ab9482, 1:1,000) antibodies were purchased from Abcam; PERK (clone D11A8, catalog number 5683, 1:1,000), IRE1α (clone 14C10, catalog number 3294, 1:1,000), ER chaperone BiP (BIP, clone C50B12, catalog number 3177, 1:1,000), activating transcription factor 4 (ATF4, clone D4B8, catalog number 11815, 1:1,000), AKT serine/threonine kinase (AKT, polyclonal, catalog number 9272, 1:1,000), P-AKT (S473) (polyclonal, catalog number 9271, 1:1,000), P-AKT (T308) (polyclonal, catalog number 9275, 1:1,000), AMP-activated protein kinase catalytic subunit alpha 1 (AMPKα1, polyclonal, catalog number 2795, 1:1,000), AMP-activated protein kinase catalytic subunit alpha 2 (AMPKα2, polyclonal, catalog number 2757, 1:1,000), AMPKα (clone D5A2, catalog number 5831, 1:1,000), P-AMPKα (T172) (clone 40H9, catalog number 2535, 1:1,000), caspase-3 (clone D3R6Y, catalog number 14420, 1:1,000), caspase-9 (clone C9, catalog number 9508, 1:1,000), SMAD2 and SMAD3 (clone D7G7, catalog number 8685, 1:1,000), P-SMAD2 (S465/S467) (clone 138D4, catalog number 3108, 1:1,000) and P-SMAD3 (S423/S425) (clone C25A9, catalog number 9520, 1:1,000) antibodies from Cell Signaling Technology; a P-IRE1α (S724) (polyclonal, catalog number PA1-16927, 1:1,000) antibody from Invitrogen; a human CRELD2 (polyclonal, catalog number MBS2527807, 1:1,000) antibody from MyBioSource; mouse CRELD2 (polyclonal, catalog number AF3686, 1:1,000) and human mesencephalic astrocyte-derived neurotrophic factor (MANF, polyclonal, catalog number AF3748, 1:1,000) antibodies from R&D Systems; and a collagen type I alpha 1 chain (COL1A1, clone 3G3, catalog number sc-293182, 1:1,000) antibody from Santa Cruz Biotechnology.

### Protein deglycosylation

To analyze the glycosylation status of CRELD2, protein extracts were treated with protein deglycosylation mix II (New England Biolabs, catalog number P6044S) before gel electrophoresis and immunoblotting according to the manufacturer’s instructions.

### CRELD2-nAb

We generated a recombinant monoclonal CRELD2-nAb using Bio-Rad’s Human Combinatorial Antibody Libraries (HuCAL) platform. In brief, the fully synthetic HuCAL PLATINUM phage display antibody library encoding ∼4.5 × 10^10^ Fab fragments was screened using CysDisplay technology to identify Fab fragments binding to recombinant mouse CRELD2 (R&D Systems, catalogue number 3686-CR)^50^. After three rounds of phage panning, nine Fab fragments binding to CRELD2 were identified and tested for their CRELD2-neutralizing activity in a HUVEC monolayer scratch assay. The Fab fragment exhibiting the greatest inhibitory activity in this screen was converted into a human (Fab)–mouse (Fc) chimeric IgG1 antibody (nAb). A chimeric IgG1 antibody against green fluorescent protein (GFP) was used as a negative control (conAb).

### Adenoviruses

Adenoviruses encoding wild-type CRELD2, CRELD2 lacking its ER retention signal (CRELD2–ΔREDL) or CRELD2 in which RDEL was replaced by KDEL (CRELD2–KDEL) were generated with the AdEasy adenoviral vector system (Agilent Technologies).

### Lentiviruses

shRNAs were designed using the Genetic Perturbation Platform provided by the Broad Institute (https://portals.broadinstitute.org/gpp/public/). We applied shRNAs targeting *ATF6* (shRNA construct identifier, TRCN0000017857), endoplasmic reticulum to nucleus signaling 1 (*ERN1*, encoding IRE1α; TRCN0000195378), eukaryotic translation initiation factor 2 alpha kinase 3 (*EIF2AK3*, encoding PERK; TRCN0000195162), protein kinase AMP-activated catalytic subunit alpha 1 (*PRKAA1*, encoding AMPKα1; TRCN0000219690), protein kinase AMP-activated catalytic subunit alpha 2 (*PRKAA2*, encoding AMPKα2; TRCN0000194959) and AKT serine/threonine kinase 1 (*AKT1*; TRCN0000221552). A scrambled (sh)RNA was used as a negative control (a gift from D. M. Sabatini; Addgene, catalog number 1864). Double-stranded shRNA-encoding oligonucleotides were cloned into the pLKO.1-TRC plasmid (a gift from D. E. Root; Addgene, catalog number 10879). All generated plasmids were validated by Sanger sequencing (Eurofins Genomics). Lentiviruses were produced by co-transfecting HEK-293T cells (American Type Culture Collection, catalog number CRL-3216) with shRNA-encoding pLKO.1-TRC plasmids, the pCMV-dR8.2 dvpr lentiviral packaging plasmid (a gift from R. A. Weinberg; Addgene, catalog number 8455) and the envelope protein-encoding pCMV-VSV-G plasmid (a gift from R. A. Weinberg; Addgene, catalog number 8454). Viral titers were determined by the qPCR lentivirus titer kit (Applied Biological Materials).

### Cell culture and functional assays

HUVECs were purchased from Provitro and HCAECs from Provitro or Lonza. Cells were expanded in 75 cm² flasks (Nunc) in growth medium (EGM-2, Lonza; 12% fetal bovine serum (FBS), Capricorn Scientific; penicillin and streptomycin, Gibco). For functional assays^6^, cells from passages 3–6 were switched to maintenance medium (MCDB 131 medium, Gibco; 2% FBS; 1 g l^−1^ l-glutamine). Gap closure after scratch injury was analyzed in endothelial cell monolayers grown in 24-well plates (Techno Plastic Products). Monolayers were scratched with a 200 μl pipet tip, washed and stimulated with various agents for 16 h. Before and after stimulation, digital phase contrast images were acquired with a Zeiss Axio Observer.Z1 microscope and analyzed using Zeiss AxioVision software 4.9. Recovery (%) was calculated as ([cell-free area at 0 h − cell-free area at 16 h] / cell-free area at 0 h) × 100. To measure endothelial proliferation, cells were grown in 12-well plates (Techno Plastic Products, 5 × 10^4^ cells per well), switched to maintenance medium and stimulated for 48 h. During the final 5 h, 10 μmol l^−1^ EdU (Invitrogen) was added to the culture medium. Thereafter, all nuclei were stained with 4′,6-diamidino-2-phenyl-indol-dihydrochloride (DAPI), whereas EdU^+^ nuclei were visualized with Alexa Fluor 488-labeled azide dye (Click-iT EdU kit, Invitrogen, catalog number C10337). EdU positivity was determined by fluorescence microscopy (Zeiss Axio Observer.Z1). Branching morphogenesis was assessed on growth-factor-reduced Matrigel (Corning) in 48-well plates (9 × 10^3^ cells per well). After stimulation for 4 h, cells were stained with 2′,7′-bis-(2-carboxyethyl)-5(6)-carboxyfluorescein-tetrakis-acetoxymethylester (BCECF-AM, Sigma-Aldrich, 1 μg ml^−1^). Digital images were acquired by fluorescence microscopy. Branching points were defined as intersections of at least three tubes.

Neonatal rat ventricular cardiomyocytes (NRCMs) were isolated from 1–3-day-old Sprague Dawley rats by Percoll density gradient centrifugation. NRCMs were plated overnight on gelatin-coated culture dishes in Dulbecco’s modified Eagle medium (DMEM, Capricorn Scientific, 4 parts) and medium 199 (Sigma-Aldrich, 1 part), supplemented with 5% horse serum, 2.5% fetal calf serum, l-glutamine, penicillin and streptomycin (plating medium). Afterward, cells were switched to DMEM and medium 199 supplemented only with l-glutamine and antibiotics (maintenance medium).

Mouse embryonic fibroblasts (American Type Culture Collection, catalog number SCRC-1040) were expanded in 75 cm² flasks in growth medium (DMEM, 10% FBS, penicillin and streptomycin). For functional assays, cells from passages 10–14 were switched to maintenance medium (DMEM, 1% FBS, penicillin and streptomycin). Gap closure after scratch injury (scratch with a 1,000 μl pipet tip) and cell proliferation (12-well plates, 8 × 10^4^ cells per well) were analyzed as described for HCAECs above.

To simulate ischemia, HCAECs and NRCMs were incubated in a buffer solution containing (in mmol l^−1^) 137 NaCl, 12 KCl, 0.5 MgCl_2_, 0.9 CaCl_2_, 4 HEPES, 10 2-deoxyglucose and 20 sodium lactate (pH 6.2) in a 5% CO_2_ and 95% N_2_ atmosphere. Control (no ischemia) HCAECs were cultured in MCDB 131 medium containing 1 g l^−1^ l-glutamine in 5% CO_2_ and 95% room air. Control NRCMs were cultured in maintenance medium in 5% CO_2_ and 95% room air. To simulate reperfusion, HCAECs were switched to MCDB 131 medium with 1 g l^−1^ l-glutamine and NRCMs were switched to maintenance medium, both in 5% CO_2_ and 95% room air^5^.

DNA fragmentation was assessed using in situ terminal deoxynucleotidyl transferase-mediated dUTP nick end-labeling (TUNEL) using the ApopTag fluorescein in situ apoptosis detection kit (Sigma-Aldrich).

An expression plasmid (pcDNA/V5-HisA, Invitrogen) encoding the N-terminal fragment of ATF6 (ATF6p50) was transfected into HUVECs using X-tremeGENE HP DNA transfection reagent (Roche); pcDNA/V5-HisA encoding GFP served as control. After 6 h, the cells were switched to fresh growth medium and cultured for an additional 42 h. Conditioned supernatants and cell lysates were collected after an additional 48 h in MCDB 131 medium containing 1 g l^−1^ l-glutamine. Supernatants were concentrated using Amicon Ultra-15 centrifugal filters (Merck, catalog number UFC901024).

HCAECs were infected with adenoviruses in growth medium (multiplicity of infection of 50; as determined by the Adeno-X rapid titer kit from Takara). After 24 h, cells were switched to maintenance medium and used for functional assays. HCAECs were infected with lentiviruses in growth medium (12 virus particles per cell, as determined by the qPCR lentivirus titer kit from Applied Biological Materials). After 24 h, cells were switched to fresh growth medium and cultured for an additional 72 h. Thereafter, cells were switched to maintenance medium and used for functional assays.

### RT-qPCR

Total RNA was isolated using RNeasy kits (Qiagen) and reverse transcribed into cDNA using SuperScript III reverse transcriptase (Thermo Fisher Scientific). cDNA was subjected to qPCR using SYBR green or TaqMan assays (reagents from Thermo Fisher Scientific). Human *CRELD2* (primer sequences, TTCTTACCCGCCTTGCTGTC and ACACTCGTCCACATCCACAC) and *GAPDH* (ACATCAAGAAGGTGGTGAAGCAGG, AGCTTGACAAAGTGGTCGTTGAGG), and mouse *Acta2* (GGATGCAGAAGGAGATCACAG, TGGAAGGTAGACAGCGAAG), *Col1a1* (TGGTTTGGAGAGAGCATGACCGAT, TAGGCTACGCTGTTCTTGCAGTGA) and *Gapdh* (CTGAGTATGTCGTGGAGTCTACTG, GCAGGATGCATTGCTGACATTC), were quantified using SYBR green assays. Mouse *Creld2* (catalog number 4331182; assay ID, Mm01309160_m1) and *Gapdh* (catalog number 4448490; assay ID, Mm99999915_g1) were quantified using TaqMan assays.

To detect *XBP1* mRNA splicing, total RNA was reverse transcribed into cDNA and analyzed by PCR (primer sequences, AGGAGTTAAGACAGCGCTTGGGGATGGAT and CTGAATCTGAAGAGTCAATACCGCCAGAAT) using the following cycling conditions: 98 °C for 2 min, then 35 cycles of 98 °C for 20 s, 61 °C for 15 s, 72 °C for 20 s and, finally, 72 °C for 5 min. PCR products were resolved on a 5% agarose gel and stained with Midori green (Nippon Genetics) to visualize spliced, unspliced and hybrid mRNA variants. Gels were imaged with an InGenius3 gel documentation system (Syngene).

### scRNA-seq and bioinformatic analysis

We performed scRNA-seq of CD45^low^ CD31^high^ ECs isolated by fluorescence-activated cell sorting (FACS) from the infarct region (anteroapical wall distal to the coronary artery ligation site, representing the infarct core and border zone) 3 days after sham or MI surgery^7^. The raw data have been deposited in Gene Expression Omnibus (GEO; accession number, GSE198401). Using 10x Genomics Cell Ranger software 4.0.0, read data were aligned to the *Mus musculus* reference genome (dataset, refdata-gex-mm10-2020-A) provided by 10x Genomics. Aligned reads per gene were counted, and clustering and summary statistics were calculated. Data outputs from all samples were aggregated and normalized to the same sequencing depth. Feature-barcode matrices were recomputed and clustering was performed using the cell ranger aggr pipeline. Annotated clusters were viewed and revised using *t*-distributed stochastic neighbor embedding (t-SNE, 10x Genomics Loupe Browser software 6.3.0). For visualization, Loupe Browser data and projections were imported into RStudio (2022.07.1 + 554 and R4.2.1). Each dataset was adjusted to 11,743 cells and visualized using Seurat 4.1.1 and ggplot2 3.3.6. ECs experiencing ER stress were identified by increased (log_2_ ratio > 1) expression of the marker genes *Hspa5* (encoding ER chaperone BiP) and *Manf*^15,21^. The *Hspa5*^high^ *Manf* ^high^ EC population comprised 9.2% of all ECs after MI.

Based on 299 genes that were differentially expressed (*P* < 0.05, expression level > 0.1) in *Hspa5*^high^ *Manf* ^high^ versus non-*Hspa5*^high^ *Manf* ^high^ cardiac ECs after MI, Ingenuity Pathway Analysis software (released July 2023, Qiagen) was used to functionally annotate the *Hspa5*^high^ *Manf* ^high^ EC population. Annotations based on >20 genes, not specifically referring to nonendothelial cell types, and with a *z*-score > 1.5 (enriched) or <−1.5 (downregulated) are reported.

Using Loupe Browser, we identified 948 protein-coding transcripts that were preferentially expressed (>1.5-fold, *P* < 0.05) in this EC population after MI. Based on their predicted amino acid sequences, 55 of these proteins (expression > 0.1) were putatively secreted via the classical secretory pathway as they contained an N-terminal signal peptide (SignalP 4.1, https://services.healthtech.dtu.dk/service.php?SignalP-4.1) but no transmembrane domains (TMHMM 2.0, https://services.healthtech.dtu.dk/service.php?TMHMM-2.0). These factors were further screened for their ER stress inducibility in HCAECs exposed to Tg (2 µmol l^−1^) or Tm (2.5 µmol l^−1^) as determined by RT-qPCR using SYBR green assays (reagents from Thermo Fisher Scientific; primer sequences are listed in Supplementary Table 2).

### snRNA-seq dataset

Using Single-Cell Analysis software (scanpy 1.9.1), we interrogated a published snRNA-seq dataset^27^ to assess *CRELD2* mRNA expression in specific cell types in the infarcted human heart. Data were downloaded from https://zenodo.org/record/6578047. The control (no MI), ischemic zone (after MI) and remote zone (after MI) groups comprised 41,663, 7,993 and 42,863 nuclei, respectively. We randomly selected 7,993 nuclei from the control and remote zone groups to generate a new data object with an equal number of nuclei across all three conditions.

### Chromatin immunoprecipitation

Chromatin immunoprecipitation (ChIP) was performed with the Pierce magnetic ChIP kit (Thermo Fisher Scientific, catalog number 26157) according to the manufacturer’s instructions. HCAECs were fixed in 1% formaldehyde and 125 mmol l^−1^ glycine, washed twice with ice-cold phosphate-buffered saline (PBS) and suspended in ice-cold PBS containing protease and phosphatase inhibitors. After cell lysis in membrane extraction buffer containing protease and phosphatase inhibitors, nuclei were collected by centrifugation and incubated with MNase (0.5 units per 1 × 10^6^ nuclei) for 15 min at 37 °C to fragment chromatin and obtain DNA–protein complexes. Nuclear membranes were disrupted by ultrasonic homogenization, and nuclear membrane debris was separated from the nuclear lysates by centrifugation. For immunoprecipitation (IP), ATF6 (clone EPR22690-84, catalog number ab227830, Abcam) or rabbit IgG control (as provided by the ChIP kit) antibodies were applied to the nuclear lysates (2.5 μg per 1 × 10^6^ nuclei). After incubation overnight at 4 °C, ChIP-grade protein A/G magnetic beads were included in the mix, followed by an additional 2 h incubation at 4 °C. The beads were washed thrice in IP wash buffer 1 and once in IP wash buffer 2. Captured DNA–protein complexes were eluted in IP elution buffer for 30 min at 65 °C. Proteins in the eluted fractions were digested with 0.25 μg μl^−1^ proteinase K in 190 mmol l^−1^ NaCl for 1.5 h at 65 °C. DNA was then purified using DNA cleanup columns. The purified DNA was subjected to PCR using primers flanking the ATF6 binding site in the *CRELD2* promoter region^51^ (forward, ACTCCTGGCGGCCGCTGAT; reverse, CCCCGCGCCGCTATTGGTT) and the following touchdown PCR program: 98 °C for 2 min, then 16 cycles: 98 °C for 20 s, 73 °C to 58 °C (dropping 1 °C per cycle) for 15 s and 72 °C for 20 s; 30 cycles: 98 °C for 20 s, 58 °C for 15 s and 72 °C for 20 s; and, finally, 72 °C for 5 min. PCR products were separated on a 3% agarose gel, stained with Midori green and imaged using the InGenius3 gel documentation system.

### Phosphoproteomics

Following our previously described phosphoproteomics workflow^7^, HCAECs (1.2 × 10^6^ cells per replicate) were solubilized with ice-cold urea buffer containing cOmplete Mini protease inhibitor cocktail (Roche) and PhosSTOP (Roche), and frozen overnight at −80 °C. After thawing, samples were dispersed and centrifuged. Then, 300 μg protein from the supernatant was loaded onto a 10 kDa Amicon Ultra-0.5 ml centrifugal filter (Merck), using a filter-aided sample preparation protocol^7,52^. After carbamidomethylation of free cysteines, proteins were trypsin digested overnight. Phosphopeptides were enriched (Pierce TiO_2_ and Fe-NTA phosphopeptide enrichment kits) and fractionated (Pierce high-pH reversed-phase peptide fractionation kit; all from Thermo Fisher Scientific). The five fractions per replicate and condition were each reconstituted in high-performance liquid chromatography loading buffer and separately analyzed using an UltiMate 3000 RSLCnano system (Thermo Scientific) connected to an Orbitrap Fusion Lumos Tribrid mass spectrometer (Thermo Fisher Scientific). Peptide scans were conducted after higher-energy collisional dissociation in the Orbitrap mass analyzer in 3 s duty cycles, with the intensity threshold set to 2,000 counts, a maximum injection time of 54 ms and a quadrupole isolation window of 0.8 m/z. Generated spectra were analyzed with MaxQuant software 1.6.17, applying the integrated Andromeda search engine against the human UniProt Knowledgebase^53^. Phosphorylation (S/T/Y), oxidation (M), deamidation (N/Q) and N-terminal acetylation were set as variable modifications, and carbamidomethylation (C) was set as fixed modification. Potential contaminants, reverse database hits and phosphopeptides with a localization probability below 0.75 were excluded. Phosphosites not detectable in all four replicates from at least one experimental condition were filtered out. Missing values were imputed based on the normal distribution of all measured log_2_-transformed intensities using Perseus software 1.6.14, applying a width of 0.3 and a downshift of 1.8 separately for each replicate^54^. Principal component analysis and hierarchical clustering were based on all CRELD2- and/or VEGFA-responsive phosphosites (analysis of variance (ANOVA), *P* < 0.05). Unsupervised hierarchical clustering was performed with the ComplexHeatmap package 2.18.0 for R using Ward’s method and Euclidean distance as a similarity metric. Linear kinase motif enrichment analysis was performed based on phosphorylation site annotations from the PhosphoSitePlus database (https://www.phosphosite.org/)^49^. The kinase recognition motif around the measured phosphorylation sites was categorically enriched with Fisher exact test for each stimulation time point. For each significantly regulated kinase (Benjamini–Hochberg false discovery rate < 0.02), all potential targets were extracted (kinase–substrate recognition motif positive, localization probability > 0.75, two-sided unpaired *t*-test, *P* < 0.05) and the arithmetic mean regulation was determined.

### Mice

*Creld2*-deficient mice, in which the coding region of *Creld2* has been replaced by an enhanced GFP-coding sequence (B6.129P2(SJL)-*Creld2*^*tm1.1Emass*^/J), have previously been described by our group^28^ and have been donated to the Jackson Laboratory (strain category number 037012). The line was maintained by heterozygous matings and mice were genotyped by genomic PCR^28^. Tg(TIE2GFP)287Sato/J mice expressing GFP in ECs under the control of the *Tek* (TEK receptor tyrosine kinase, also known as *Tie2*) promoter were obtained from the Jackson Laboratory (strain number 003658)^55^. Wild-type C57BL/6J mice were purchased from Charles River (strain code 632).

### Mouse surgery

Mice were housed in individually ventilated cages in a 12 h light and dark cycle in the central animal facility of Hannover Medical School. Food and water were provided ad libitum. We induced MI in 8–10-week-old male mice by transient left anterior descending coronary artery (LAD) ligation. Mice were subcutaneously pretreated with 0.02 mg kg^−1^ atropine (B. Braun) and 2 mg kg^−1^ butorphanol (Zoetis). Anesthesia was induced with 3–4% isoflurane (Baxter) in an induction chamber. After oral intubation, mice were mechanically ventilated (Harvard Apparatus, MiniVent type 845) and anesthesia was maintained with 1.5–2% isoflurane. Mice were placed on a heating pad connected to a temperature controller (Föhr Medical Instruments) to keep rectal temperature at 37 °C during surgery. A left thoracotomy was performed, and the LAD was ligated using a Prolene 7-0 (Ethicon) suture against a PE-10 tube (ischemia). The tube was removed 1 h later, and reperfusion was visually confirmed by light microscopy. In sham-operated mice, the ligature around the LAD was not tied. After surgery, mice were transferred to a 32 °C incubator for recovery.

### Assessing heart function

Echocardiography was performed with a linear 20–46 MHz transducer (MX400, Vevo 3100, VisualSonics) in mice sedated with 0.5–1% isoflurane via face mask. LV end-diastolic area (LVEDA) and LV end-systolic area (LVESA) were recorded from the parasternal long-axis view. As a measure of systolic function, fractional area change (FAC, %) was calculated as [(LVEDA − LVESA) / LVEDA] × 100. For conductance catheterization, mice were subcutaneously injected with 2 mg kg^−1^ butorphanol. Anesthesia was induced with 3–4% isoflurane. After oral intubation, mice were mechanically ventilated and anesthesia was maintained with 2% isoflurane. LV pressure–volume loops were recorded with a 1.4-French micromanometer-tipped conductance catheter inserted via the right carotid artery (SPR-839, Millar Instruments). Steady-state pressure–volume loops were sampled at a rate of 1 kHz and analyzed with LabChart software 7 Pro (ADInstruments). At the end of the protocol, animals were euthanized by cervical dislocation during ongoing isoflurane anesthesia.

### CRELD2 protein therapy and antibody-mediated neutralization

Recombinant CRELD2 (10 μg) or an equal volume of PBS (control) was injected into the LV cavity at the time of reperfusion. Thereafter, recombinant CRELD2 (10 μg per day) or PBS was infused for 7 days using Alzet osmotic minipumps (model 1007D) implanted in a subcutaneous interscapular pocket. CRELD2-nAb or conAb (100 μg each) was injected into the LV cavity at the time of reperfusion (matching the time point in the sham-operated mice).

### Tissue collection and analyses

Mice were killed at different time points after MI or sham operation, and the left ventricles were removed. Area at risk and infarct size were determined by Evans blue and 2,3,5-triphenyltetrazolium chloride (TTC, Sigma-Aldrich) staining of basal, midventricular and apical 1 mm LV tissue slices and computerized planimetry. For later isolation of RNA and protein, the left ventricles were divided into the infarct region (anteroapical wall distal to the coronary artery ligation site representing the infarct core and border zone) and remote (noninfarcted) region (basal part of the interventricular septum). Tissues were snap-frozen in liquid N_2_ and stored at −80 °C. Corresponding parts of the left ventricle were obtained after sham surgery. For histology, basal, midventricular and apical LV tissue slices were embedded in OCT compound (Tissue-Tek), snap-frozen in liquid N_2_ and stored at −80 °C. Scar size was calculated as the average ratio of the scar area to the total LV area in basal, midventricular and apical 10 μm cryosections stained with Masson’s trichrome (Sigma-Aldrich). For fluorescence microscopy, 6 μm cryosections were cut from midventricular slices. Images were acquired with the Axio Observer.Z1 microscope. Sections were stained with rhodamine-conjugated wheat germ agglutinin (Vector Laboratories, 1:200) to highlight ECM and cardiomyocyte borders and with DAPI (Sigma-Aldrich) to identify cell nuclei. ECs were detected by fluorescein-labeled GSL I isolectin B4 (IB4, Vector Laboratories, 1:100) staining or immunofluorescence microscopy using a CD31 antibody (clone MEC 13.3, BD Biosciences, catalog number 553370, dilution 1:100) and a DyLight 550-labeled secondary antibody (polyclonal, Abcam, catalog number ab98406, 1:200). To detect proliferating ECs, mice were intraperitoneally injected with 5-ethynyl-2′-deoxyuridine (EdU, Thermo Fisher Scientific, 50 mg kg^−1^) and midventricular cryosections were prepared after 24 h. After CD31 immunostaining, EdU^+^ ECs were identified using the Click-iT plus EdU Alexa Fluor 594 imaging kit (Invitrogen). Mice were injected via the tail vein with 50 μg Alexa Fluor 488-conjugated GS IB4 (Invitrogen) to label perfused capillaries. Mice were killed 1 h later, and midventricular cryosections were prepared. Perfused and nonperfused capillaries were visualized by CD31 immunostaining. Ki67 was detected with an antibody from Abcam (polyclonal, catalog number ab15580, 1:100) and a Cy3-labeled secondary antibody (Jackson ImmunoResearch, polyclonal, catalog number 111-165-144, 1:200). CRELD2 was visualized by confocal immunofluorescence microscopy (Leica DM IRB with a TCS SP2 AOBS scan head) after staining with an antibody from R&D Systems (polyclonal, catalog number AF3686, 1:100) and an Alexa Fluor 488-conjugated secondary antibody (Invitrogen, polyclonal, catalog number A-11055, 1:100).

Apoptotic cardiomyocytes were identified by TUNEL (ApopTag fluorescein in situ apoptosis detection kit, Merck) and co-staining with DAPI and an alpha-actinin antibody (clone EA-53, catalog number A7732, 1:400) and a TRITC-labeled secondary antibody (polyclonal, catalog number T5393, 1:120) from Sigma-Aldrich. In pilot experiments, all secondary antibodies were confirmed to yield low background signals.

### Cell sorting

FACS and flow cytometry methods have previously been described^5,56,57^. Briefly, the infarct region of the left ventricle was digested with collagenase D (Roche), DNase I (Sigma-Aldrich) and dispase (Gibco) and processed with a gentleMACS dissociator (Miltenyi Biotec). Cell suspensions were filtered (40 μm cell strainer, Falcon), washed and incubated for 5 min at 4 °C in PBS with 4% FBS, 2 mmol l^−1^ EDTA and a purified mouse CD16/CD32 antibody (clone 2.4G2, mouse BD Fc Block, BD Biosciences, catalog number 553141, 1:55). For inflammatory cell isolation by FACS, cells were incubated for 20 min at 4 °C with the following antibodies: CD45R/B220-PE (clone RA3-6B2, 1:500), CD90.2/Thy-1.2-PE (clone 53-2.1, 1:2,500), NK-1.1-PE (clone PK136, 1:500), CD49b/DX5-PE (clone DX5, 1:500), Ly6G-PE (clone 1A8, 1:500), I-Ab-FITC (clone AF6-120.1, 1:500) and CD11b-Alexa Fluor 700 (clone M1/70, 1:50) from BD Biosciences; CD45-Brilliant Violet 570 (clone 30-F11, 1:33), F4/80-FITC (clone BM8, 1:33), CD3-PE/Cy7 (clone 17A2, 1:33) and CD19-PerCP/Cy5.5 (clone 6D5, 1:33) from BioLegend; and CD11c-FITC (clone N418, 1:8) and Ly6C-APC (clone 1G7.G10, 1:8) from Miltenyi Biotec. After washing, the cells were sorted on a FACSAria IIu instrument (Becton Dickinson). Ly6C^high^ monocytes were identified as CD45^high^ CD11b^high^ (CD45R/B220, CD90.2/Thy-1.2, NK-1.1, CD49b/DX5, Ly6G)^low^ (CD11c, F4/80, I-Ab)^low^ Ly6C^high^; Ly6C^low^ monocytes or macrophages as CD45^high^ CD11b^high^ (CD45R/B220, CD90.2/Thy-1.2, NK-1.1, CD49b/DX5, Ly6G)^low^ (CD11c, F4/80, I-Ab)^high/low^ Ly6C^low^; neutrophils as CD45^high^ CD11b^high^ (CD45R/B220, CD90.2/Thy-1.2, NK-1.1, CD49b/DX5, Ly6G)^high^; T cells as CD45^high^ CD11b^low^ (CD45R/B220, CD90.2/Thy-1.2, NK-1.1, CD49b/DX5, Ly6G)^high^ CD3^high^ CD19^low^; and B cells as CD45^high^ CD11b^low^ (CD45R/B220, CD90.2/Thy-1.2, NK-1.1, CD49b/DX5, Ly6G)^high^ CD3^low^ CD19^high^. For flow cytometry, inflammatory cells from the infarct region were incubated with labeled antibodies as described above. Cells were then added to TruCOUNT tubes (BD Biosciences), counted on an LSR II flow cytometer (Becton Dickinson) and analyzed with FlowJo software 10.7.2.

### Cell isolation

ECs and fibroblasts were isolated by magnetic-activated cell sorting (protocols, reagents and equipment from Miltenyi Biotec). In brief, LV myocardium from the infarct region was digested with collagenase I (Worthington) and DNase I (Sigma-Aldrich). Cell suspensions were filtered (40 μm cell strainer, Falcon), washed and incubated with CD45 MicroBeads and applied to LD columns. The flow-through CD45^low^ cell fraction was washed, incubated with CD146 MicroBeads and reapplied to LD columns. CD45^low^ CD146^high^ ECs were eluted from the columns. The flow-through CD45^low^ CD146^low^ cell fraction was washed, incubated with feeder removal MicroBeads and reapplied to LS columns. Fibroblasts were eluted from the columns. Adult mouse ventricular cardiomyocytes were isolated by enzymatic digestion using a protocol provided by the Alliance for Cellular Signaling^49^.

### LV tissue explants

MI was induced in *Tie2*-GFP mice. After 3 days, tissue samples (∼1 mm^3^) were obtained from the infarct region of the left ventricle and cultured in 48-well plates (1 sample per well) on growth factor-reduced Matrigel (Corning) in DMEM-HA medium (Capricorn Scientific) supplemented with 2% FBS, 25 mmol l^−1^ HEPES, penicillin and streptomycin in the absence or presence of CRELD2 or VEGFA and/or small-molecule inhibitors. After 3 days, explants were fixed in 4% paraformaldehyde and green fluorescent endothelial sprouts were imaged by fluorescence microscopy. Average sprout length was calculated from eight random sprouts per sample^6^. LV tissue explants were infected with adenoviruses (2 × 10^6^ infectious virus particles per explant) in DMEM-HA medium supplemented with 20% FBS, 25 mmol l^−1^ HEPES, 1 mmol l^−1^ sodium pyruvate, 100 μg ml^−1^ endothelial cell growth supplement (PromoCell, catalog number C-30140), nonessential amino acids (Gibco, catalog number 11140050), penicillin and streptomycin. After 24 h, explants were used for sprouting assays.

### Isolating ECM proteins

ECM proteins were isolated from tissue samples from the infarct region (or corresponding parts of the left ventricle after sham surgery) with the Millipore compartment protein extraction kit (catalog number 2145) according to the manufacturer’s protocol. CRELD2 expression in the ECM was normalized to collagen type I alpha 1 chain expression.

### Detecting HS-bound CRELD2

LV tissue samples were digested with heparinases to depolymerize HS polysaccharide chains and release HS-bound proteins into the tissue supernatant^25^. To this end, ∼10 mg samples from the infarct region (or corresponding parts of the left ventricle after sham surgery) were digested with *Bacteroides* heparinases I (180 units ml^−1^), II (60 units ml^−1^) and III (10 units ml^−1^, all from New England Biolabs) in 100 μl buffer containing (in mmol l^−1^) 100 NaCl, 1.5 CaCl_2_ and 20 Tris–HCl (pH 7.0). In control tubes, the heparinases were omitted from the reactions. After 18 h at 30 °C, tubes were centrifuged for 10 min at 16,000 × *g* and CRELD2 in the supernatants was measured by ELISA (RayBiotech; human CRELD2, catalog number ELH-CRELD2-1; mouse CRELD2, catalog number ELM-CRELD2-1). Protein abundance was calculated as protein abundance in supernatants from reactions with heparinases minus protein abundance in supernatants from reactions without heparinases. ELISA results were normalized to LV tissue input (in mg).

### Human myocardial tissue and plasma samples

We collected LV myocardial tissue samples at autopsy from 12 patients (45–96 years old; 6 men and 6 women; all white) who had died of an acute MI (samples were taken from the infarcted myocardium) and from 9 patients (56–97 years old; 7 men and 2 women; all white) who had died from noncardiac causes (controls). Autopsies were performed within 24 h after death, and tissue samples were stored in liquid N_2_ for later immunoblotting analyses. We collected EDTA-treated plasma samples from 8 patients (46–71 years old; 6 men and 2 women; all white) admitted to Hannover Medical School with acute ST-segment-elevation MI. Plasma samples were obtained 1, 3 and 5 days after primary coronary intervention. We also obtained EDTA plasma samples from 7 apparently healthy individuals (52–69 years old; 5 men and 2 women; all white) recruited at Heidelberg University^58^. Plasma samples were stored at −80 °C.

### Statistical analyses

Sample sizes were chosen based on our previous experience with angiogenic growth factors driving infarct repair^5–7^. Mouse littermates were used in all experiments and randomly allocated to the experimental groups. All mice surviving until the end of the experiment were included in the analyses. Positive and negative controls were included in all cell culture experiments. When these controls yielded the expected results, we included all samples from that experiment in the analyses. Whenever possible, investigators were blinded to group allocation during the experiment and when assessing outcome. All data presented in the paper were found to be reproducible in independent experiments. Unless otherwise stated, summary data are presented as mean values with s.e.m. Whenever possible, results from individual mice and independent cell culture experiments are reported. The two-sided independent-sample *t*-test was used to compare two groups. For comparisons among more than two groups, one-way ANOVA was used if there was one independent variable and two-way ANOVA if there were two independent variables. We used the Dunnett test to adjust for multiple comparisons with a single control group, the Tukey test to adjust for comparisons between multiple groups and the Sidak test to adjust for pairwise comparisons between selected groups. Expression matrices in the snRNA-seq dataset were compared using the Wilcoxon rank sum test. The analyses were performed with GraphPad Prism software 9.5. We considered a two-tailed *P* value less than 0.05 to indicate statistical significance.

### Reporting summary

Further information on research design is available in the Nature Portfolio Reporting Summary linked to this article.

### Supplementary information

Supplementary Information Supplementary Tables 1–5.

Reporting Summary

Supplementary Data 1 Source data for Supplementary Table 3.

Supplementary Data 2 Source data for Supplementary Table 4.

Supplementary Data 3 Source data for Supplementary Table 5.

### Source data

Source Data Fig. 1 Statistical source data.

Source Data Fig. 2 Unprocessed western blots and gels.

Source Data Fig. 2 Statistical source data.

Source Data Fig. 3 Unprocessed western blots.

Source Data Fig. 3 Statistical source data.

Source Data Fig. 4 Statistical source data.

Source Data Fig. 5 Unprocessed western blots.

Source Data Fig. 5 Statistical source data.

Source Data Fig. 6 Unprocessed western blots.

Source Data Fig. 6 Statistical source data.

Source Data Fig. 7 Statistical source data.

Source Data Fig. 8 Unprocessed western blots.

Source Data Fig. 8 Statistical source data.

Source Data Extended Data Fig. 1 Unprocessed western blots and gels.

Source Data Extended Data Fig. 1 Statistical source data.

Source Data Extended Data Fig. 2 Statistical source data.

Source Data Extended Data Fig. 3 Statistical source data.

Source Data Extended Data Fig. 4 Statistical source data.

Source Data Extended Data Fig. 5 Unprocessed western blots.

Source Data Extended Data Fig. 5 Statistical source data.

Source Data Extended Data Fig. 6 Statistical source data.

## Data Availability

We have deposited the scRNA-seq raw data in Gene Expression Omnibus (GEO; accession number, GSE198401). Human snRNA-seq data are publicly available at the Zenodo data archive (https://zenodo.org/record/6578047). We have deposited the phosphoproteomics dataset to the ProteomeXchange consortium via the PRIDE partner repository (dataset identifier, PXD045013). All other data supporting the findings in this study are included in the main article and its associated files.Source data are provided with this paper.
