# Supplementary material for: Cysteine-rich with EGF-like domains 2 (CRELD2) is an endoplasmic reticulum stress-inducible angiogenic growth factor promoting ischemic heart repair
Source: Nat Cardiovasc Res. 2024 Jan 17;3(2):186–202. doi: 10.1038/s44161-023-00411-x (PMC11358006; doi:10.1038/s44161-023-00411-x)
Supplement: Supplementary file 1 — Supplementary Tables 1–5. [file 44161_2023_411_MOESM1_ESM.pdf]

nature cardiovascular research

Article https://doi.org/10.1038/s44161-023-00411-x

Cysteine-rich with EGF-like domains 2 (CRELD2) is an endoplasmic reticulum stress-inducible angiogenic growth factor promoting ischemic heart repair

In the format provided by the authors and unedited

Supplementary data table 1. Functional annotation of *Hspa5*^{high} *Manf*^{high} endothelial cells after MI.

Annotations	z-score	*P* value	Number of genes
Cell survival	4.18	9.70×10^{-20}	92
Cell viability	4.04	3.01×10^{-18}	87
Cell movement	3.63	2.10×10^{-21}	124
Migration of cells	3.24	7.12×10^{-21}	116
Formation of cellular protrusions	2.98	2.39×10^{-7}	49
Microtubule dynamics	2.88	2.76×10^{-13}	70
Organization of cytoplasm	2.82	7.58×10^{-12}	77
Organization of cytoskeleton	2.82	6.91×10^{-13}	75
M phase	2.74	6.77×10^{-10}	21
Invasion of cells	2.74	2.35×10^{-20}	83
Cell cycle progression	2.73	2.14×10^{-18}	68
Chemotaxis	2.46	1.78×10^{-14}	41
G1 phase	2.18	1.66×10^{-8}	29
Synthesis of DNA	2.07	7.92×10^{-11}	29
Interaction of DNA	1.94	7.85×10^{-10}	32
Colony formation	1.83	2.60×10^{-13}	47
Organization of organelle	1.80	7.45×10^{-9}	39
Stimulation of cells	1.64	7.76×10^{-8}	21
Outgrowth of cells	1.59	1.64×10^{-7}	25
Binding of DNA	1.56	6.39×10^{-9}	30
Permeability of vascular system	-1.73	1.23×10^{-12}	22
Apoptosis	-3.87	1.45×10^{-28}	140

Pathway analysis based on 299 genes (expression > 0.1) that were differentially expressed ($P < 0.05$, Benjamini-Hochberg false discovery rate-corrected negative binominal exact test) in *Hspa5*^{high} *Manf*^{high} vs. non-*Hspa5*^{high} *Manf*^{high} cardiac endothelial cells after MI. Annotations with $P < 0.05$ (right-tailed Fisher exact test, no adjustments were made for multiple comparisons) and a z-score > 1.5 (enriched) or < -1.5 (downregulated) are listed.

Supplementary data table 2. Gene expression screen in human coronary artery endothelial cells.

Gene name	Symbol and gene ID	Primer sequences (matching the human orthologs)	Expression (vs. control)	
			Tg	Tm
Heat shock protein 5	*Hspa5*<br>ID: 14828	GAACGTCTGATTGGCGATG<br>ACCACCTTGAACGGCAAGAA	84.6	20.5
Procollagen C-endopeptidase enhancer protein	*Pcolce*<br>ID: 18542	GGGAATCAGGTTACGTGGCA<br>GACCTCCAGAGCATCGTAGC	66.0	15.4
Cysteine-rich with EGF-like domains 2	*Creld2*<br>ID: 76737	TTCTTACCCGCCTTGCTGTC<br>ACACTCGTCCACATCCACAC	38.8	31.8
Collagen, type XVIII, alpha 1	*Col18a1*<br>ID: 12822	TTCGTCCAGCTGTGGAATGAC<br>GCTGATGCGCTCTGAAGATGGT	36.1	35.8
Mesencephalic astrocyte-derived neurotrophic factor	*Manf*<br>ID: 74840	ACAAGCAGATCGACCTGAGCAC<br>TGCACAGCCTTTGCATGTCTCC	31.0	9.6
Stromal cell-derived factor 2-like 1	*Sdf2l1*<br>ID: 64136	CCTGGACCTATGGACAGTGC<br>CTTCCATGGCCTTCCACGTA	27.5	6.0
Secreted phosphoprotein 1	*Spp1*<br>ID: 20750	CTGAACGCGCCTTCTGATTG<br>GGGTTTCAGCACTCTGGTCA	13.7	2.8
FK506 binding protein 2	*Fkbp2*<br>ID: 14227	TGTCCTGCACATGCACTACACG<br>ATCACCAGCTTGCGCTTTTCCC	4.5	2.8
Reticulocalbin 3, EF-hand calcium binding domain	*Rcn3*<br>ID: 52377	CGTGATTGCTGAAACCCTGGAG<br>TGCCCATCCTTGTTCAGATCCC	3.9	2.2
Serine (or cysteine) peptidase inhibitor, clade H, member 1	*Serpinh1*<br>ID: 12406	GCTCTCCAGCCTCATCATCC<br>TTCTGCAGGTCATGGGTCAC	3.9	2.0
Glutathione peroxidase 7	*Gpx7*<br>ID: 67305	CGCACCTACAGTGTCTCATTCC<br>AAGCCCCTACCACCTTTCCATC	3.0	1.6
Protein disulfide isomerase associated 6	*Pdia6*<br>ID: 71853	ACTGTTCTTTGCTGGACGGCAC<br>ACCTCAAACAGCAGCACAGCAC	2.3	2.2
Platelet factor 4	*Pf4*<br>ID: 56744	TGCTGCTCCTGCCACTTG<br>TTCCATTCTTCAGCGTGGCT	2.0	1.8
Microfibrillar-associated	*Mfap4*	TTCTCTTGAAACTCGCACTCCT	1.9	2.4

protein 4	ID: 76293	ATGAGGTACACGCCGTCTGA		
Asporin	*Aspn*<br>ID: 66695	TGCGAAGGCTGTATCTGTCC<br>AGTTGGTGGTAAGCCTTTAGGA	1.8	1.3
Legumain	*Lgmn*<br>ID: 19141	CCACTGCTTCAACTGGCACT<br>AAAGGTTGACCAGCACGTACA	1.8	1.4
Out at first homolog	*Oaf*<br>ID: 102644	ACAGTTCTGTGTTCGAGGCT<br>GGCAGTACTTGAGCATGCAG	1.7	2.0
Serine (or cysteine) peptidase inhibitor, clade F, member 1	*Serpinf1*<br>ID: 20317	CCTGGTGCTACTCCTCTGCAT<br>ACAGGTCATAGCCGAAGTTGG	1.7	1.3
Collagen, type I, alpha 1	Col1a1<br>ID: 12842	ACAGAGGTTTCAGTGGTTTGGA<br>CACCAGTAGCACCATCATTTCCAC	1.6	1.3
Periostin, osteoblast specific factor	*Postn*<br>ID: 50706	ACTGAAGGACCCACACTAACA<br>TTCCACAGGCACTCCATCAA	1.6	1.0
Collagen, type I, alpha 2	*Col1a2*<br>ID: 12843	GCTCAGCTTTGTGGATACGC<br>CAAAGTTCCCACCGAGACCA	1.6	0.4
Osteoglycin	*Ogn*<br>ID: 18295	CTCCTGTTACTGCTTGTGCC<br>GCAGACACGTGGGCATTTCA	1.5	1.6
Tenascin C	*Tnc*<br>ID: 21923	AGGCAACTGTCACCTTCGAG<br>TCCATTTACGCACTTGCCCT	1.5	1.2
Fibronectin 1	*Fn1*<br>ID: 14268	GCTCAAGTGGTCCTGTCGAA<br>GTCACTTCTTGGTGGCCGTA	1.5	0.8
FK506 binding protein 10	*Fkbp10*<br>ID: 14230	ACTGTTCTTTGCTGGACGGCAC<br>ACCTCAAACAGCAGCACAGCAC	1.4	1.1
Collagen, type III, alpha 1	*Col3a1*<br>ID: 12825	AGGATCCGTTCTCTGCGATG<br>TGACCATTAGGAGGGCGAGT	1.1	1.3
Glutathione peroxidase 3	*Gpx3*<br>ID: 14778	AGAAGTCGAAGATGGACTGCC<br>CCGAATGGTGCAAGCTCTTC	1.0	0.9
Platelet derived growth factor, alpha	*Pdgfa*<br>ID: 18590	GATACCTCGCCCATGTTCTGG<br>ATGCTGTGGATCTGACTGCG	0.9	1.9
AE binding protein 1	*Aebp1*<br>ID: 11568	ACCGATGACCTGGATTTCCG<br>GTTCCCATCGCGGTACTCTC	0.9	0.6
Collagen, type V, alpha 2	*Col5a2*<br>ID: 12832	AAACACCTGGAGGTGGCAAT<br>AGGGCCAGGTCTTCCTTTTG	0.9	0.5

Microfibrillar associated protein 5	*Mfap5*<br>ID: 50530	TAGTCAACGAGGAGACGATGTG<br>CCCAGCACTCTGCAGTGGTA	0.9	0.4
Collagen, type VIII, alpha 1	*Col8a1*<br>ID: 12837	GGAGTGGCAGGACTTCATGG<br>TGCAGGCATCTCATAGGCTG	0.8	0.9
Serine (or cysteine) peptidase inhibitor, clade G, member 1	*Serping1*<br>ID: 12258	CTACCACGCCTTCTCAGCAA<br>GGTCCGAGAGGCATTCACAA	0.8	0.7
Lysyl oxidase-like 2	*Loxl2*<br>ID: 94352	GCCATCCTCTCAACCTACCG<br>CGTCCAGTGCTTGTCACAGA	0.7	0.8
Placental growth factor	*Pgf*<br>ID: 18654	TGTTCAGCCCATCCTGTGTC<br>GTGGCAGTCTGTGGGTCTC	0.7	0.4
Tissue inhibitor of metalloproteinase 1	*Timp1*<br>ID: 21857	GGCTTCTGGCATCCTGTTGT<br>TGACGAGGTCGGAATTGCAG	0.6	0.8
Lumican	*Lum*<br>ID: 17022	ACCCAAGTGCCATGTACTGT<br>GCTGCAGATCCTCCAGAGATT	0.6	0.3
Insulin-like growth factor binding protein 4	*Igfbp4*<br>ID: 16010	TCTGACAAGGACGAGGGTGA<br>GGTGCTCCGGTCTCGAATTT	0.5	0.6
Decorin	*Dcn*<br>ID: 13179	CCTGAAGAACCTTCACGCATTG<br>CCTGAGCTCTTCAGCGGATT	0.5	0.4
Thioredoxin domain containing 5	*Txndc5*<br>ID: 105245	GCCGACATGTTCACGCAC<br>CTCCCAGGTCATTCCAAGTCG	0.5	0.4
Follistatin-like 1	*Fstl1*<br>ID: 14314	AATGGTGATTCTCGCCTGGA<br>CCAGGGCACACTTCTTCTCA	0.4	0.9
Collagen, type VI, alpha 1	*Col6a1*<br>ID: 12833	TTGCTGTGAATGCAAGTGCG<br>ACGACGAAGTCCTTGGCAAT	0.4	0.7
Angiopoietin-like 4	*Angptl4*<br>ID: 57875	TCTCTGGAGGCTGGTGGTTT<br>GATCAACATGGTGGTGGCCT	0.4	0.4
Chemokine (C-C motif) ligand 8	*Ccl8*<br>ID: 20307	GCTCAGCCAGATTCAGTTTCC<br>TTCTTGTGTAGCTCTCCAGCC	0.4	0.2
Fibrillin 1	*Fbn1*<br>ID: 14118	TTGAGTGCAAATGCCCTGCT<br>GGCGAACATCTATGCATCTGG	0.3	0.8
Thrombospondin 1	*Thbs1*<br>ID: 21825	GATCCGGCTCTGCAACTCTC<br>TACATCACCAACGCAGTCCT	0.2	0.7
Cellular communication	*Ccn2*	CATCTTCGGTGGTACGGTGT	0.2	0.6

network factor 2	ID: 14219	GTCTTCCAGTCGGTAAGCCG		
Epidermal growth factor-containing fibulin-like extracellular matrix protein 2	*Efemp2*<br>ID: 58859	ATGAGTGTGAGTCTGGTGCG<br>TGTAGCGGTGCACAATGGAT	0.2	0.3
Serine (or cysteine) peptidase inhibitor, clade E, member 1	*Serpine1*<br>ID: 18787	TCAGACCAAGAGCCTCTCCA<br>TGGCAGGCAGTACAAGAGTG	0.2	0.3
Chemokine (C-X-C motif) ligand 1	*Cxcl1*<br>ID: 14825	CAGGGAATTCACCCCAAGAACA<br>GGATGCAGGATTGAGGCAAGC	0.1	0.5
Biglycan	*Bgn*<br>ID: 12111	GGATGATCGAGAACGGGAGC<br>GATGCCGTTGTAGTAGGCCC	0.1	0.4
Gelsolin	*Gsn*<br>ID: 227753	GGCGTGTGGAGAAGTTCGAT<br>CTCATCCTGGCTGCACTCAT	0.1	0.4
Lysyl oxidase-like 1	*Loxl1*<br>ID: 16949	GTGTACCGGCCCAACCAGAA<br>ACCCGCACATCGTAGTCG	0.1	0.3
Neurotensin	*Nts*<br>ID: 67405	AATCTGTCACAGCAGGGCTT<br>TTCTCATACAGCTGCCGTTTC	0.1	0.1
Chemokine (C-C motif) ligand 2	*Ccl2*<br>ID: 20296	GAAAGTCTCTGCCGCCCTT<br>GGTGACTGGGGCATTGATTG	<0.1	0.5

List of 55 genes that were preferentially expressed in *Hspa5*^{high} *Manf*^{high} cardiac endothelial cells after MI in mice and predicted to encode secreted factors. Gene names, gene symbols, and gene IDs are shown (https://www.ncbi.nlm.nih.gov/gene/). Expression of these genes in human coronary artery endothelial cells cultured for 20 hours in the absence (control) or presence of thapsigargin (Tg, 2 µmol/L) or tunicamycin (Tm, 2.5 µmol/L) was measured by RT-PCR. Primer pairs and means of 2 experiments are shown.

Supplementary data table 3. Heart size and left ventricular pressure-volume measurements in wild-type and *Creld2*-deficient mice.

	WT, sham	KO, sham	WT, MI	KO, MI
Gravimetry				
Body mass (g)	26.3 ± 0.8	26.1 ± 1.0	27.9 ± 0.6	28.4 ± 0.8
LV mass/body mass (mg/g)	3.6 ± 0.1	3.6 ± 0.1	4.1 ± 0.2	4.7 ± 0.2***,#
RV mass/body mass (mg/g)	1.4 ± 0.1	1.3 ± 0.1	1.6 ± 0.1	1.9 ± 0.3
LV pressure-volume measurements				
Heart rate (min^{-1})	488 ± 15	468 ± 18	493 ± 11	495 ± 5
LVEDP (mmHg)	6 ± 0	5 ± 2	9 ± 2	9 ± 3
LVESP (mmHg)	89 ± 3	92 ± 1	91 ± 5	91 ± 3
LVEDV (µL)	48 ± 3	53 ± 5	61 ± 6	72 ± 4*
LVESV (µL)	17 ± 2	23 ± 4	38 ± 4***	56 ± 3***,##
LVEF (%)	74 ± 4	65 ± 6	48 ± 3***	29 ± 2***,##
dP/dt_{max} (mmHg/s)	8,639 ± 378	9,323 ± 920	7,326 ± 576	7,154 ± 560
dP/dt_{min} (mmHg/s)	-8,315 ± 185	-8,703 ± 358	-6,089 ± 460**	-5,977 ± 712**
τ (ms)	7.1 ± 0.3	6.6 ± 0.3	8.9 ± 0.4	9.9 ± 1.2**
Cardiac output (mL/min)	16.5 ± 1.1	15.4 ± 1.8	13.5 ± 1.5	10.1 ± 0.8
Stroke work (mmHg × µL)	2,546 ± 117	2,646 ± 280	1,814 ± 311*	1,325 ± 175**

Wild-type (WT) and *Creld2*-deficient (knockout, KO) mice underwent sham or myocardial infarction (MI) surgery. Measurements were performed after 28 days. LV denotes left ventricular; RV, right ventricular; LVEDP, LV end-diastolic pressure; LVESP, LV end-systolic pressure; LVEDV, LV end-diastolic volume; LVESV, LV end-systolic volume; LVEF, LV ejection fraction; dP/dt_{max}, maximum rate of pressure change in the left ventricle; dP/dt_{min}, minimum rate of pressure change in the left ventricle; τ, LV diastolic time constant. 4–8 mice per group. *$P < 0.05$, **$P < 0.01$, ***$P < 0.001$ vs. same-genotype sham; #$P < 0.05$, ##$P < 0.01$ vs. WT, MI (two-way ANOVA with Sidak test).

Supplementary data table 4. Left ventricular pressure-volume measurements in wild-type mice treated with a CRELD2-neutralizing or control antibody.

	Sham, conAb	Sham, nAb	MI, conAb	MI, nAb
Heart rate (min^{-1})	508 ± 18	478 ± 18	464 ± 13	448 ± 5
LVEDP (mmHg)	5 ± 0	7 ± 1	10 ± 1*	8 ± 1
LVESP (mmHg)	91 ± 3	99 ± 1	103 ± 1**	93 ± 3$^{\#\#}$
LVEDV (µL)	52 ± 4	53 ± 5	77 ± 2***	81 ± 3***
LVESV (µL)	21 ± 3	24 ± 3	49 ± 2***	57 ± 3***
LVEF (%)	67 ± 4	61 ± 3	45 ± 2***	36 ± 2$^{***,\#}$
dP/dt_{max} (mmHg/s)	8,481 ± 249	9,017 ± 590	9,052 ± 402	7,237 ± 242$^{**,\#\#}$
dP/dt_{min} (mmHg/s)	-8,044 ± 452	-8,761 ± 683	-8,437 ± 173	-6,935 ± 403$^{*,\#}$
τ (ms)	7.1 ± 0.3	7.2 ± 0.3	7.8 ± 0.3	8.6 ± 0.2**
Cardiac output (mL/min)	17.1 ± 1.1	14.6 ± 1.3	15.6 ± 0.9	12.9 ± 0.9
Stroke work (mmHg × µL)	2,537 ± 121	2,481 ± 210	2,574 ± 133	2,052 ± 211

Wild-type mice underwent sham or myocardial infarction (MI) surgery and were injected with a CRELD2-neutralizing antibody (nAb) or control antibody (conAb). Antibodies (100 µg each) were injected into the left ventricular (LV) cavity at the time of reperfusion (matching time point in sham-operated mice). Measurements were performed after 28 days. RV denotes right ventricular; LVEDP, LV end-diastolic pressure; LVESP, LV end-systolic pressure; LVEDV, LV end-diastolic volume; LVESV, LV end-systolic volume; LVEF, LV ejection fraction; dP/dt_{max}, maximum rate of pressure change in the left ventricle; dP/dt_{min}, minimum rate of pressure change in the left ventricle; τ, LV diastolic time constant. 6–7 mice per group. *$P < 0.05$, **$P < 0.01$, ***$P < 0.001$ vs. same-antibody sham; $^{\#}P = 0.06$, $^{\#\#}P < 0.01$ vs. MI, conAb (two-way ANOVA with Sidak test).

Supplementary data table 5. Left ventricular pressure-volume measurements in wild-type mice treated with recombinant CRELD2.

	MI, control	MI, CRELD2
Heart rate (min^{-1})	419 ± 13	439 ± 8
LVEDP (mmHg)	13 ± 2	9 ± 1*
LVESP (mmHg)	93 ± 3	96 ± 4
LVEDV (µL)	81 ± 2	78 ± 2
LVESV (µL)	60 ± 3	48 ± 1***
LVEF (%)	33 ± 2	46 ± 1***
dP/dt_{max} (mmHg/s)	6,688 ± 331	8,196 ± 361**
dP/dt_{min} (mmHg/s)	-6,628± 327	-7,608 ± 273*
τ (ms)	8.7 ± 0.5	7.3 ± 0.2*
Cardiac output (mL/min)	11.0 ± 0.5	15.4 ± 0.8**
Stroke work (mmHg × µL)	1,876 ± 60	2,561 ± 133***

Wild-type mice underwent myocardial infarction (MI) surgery. Recombinant CRELD2 (10 µg) was injected into the left ventricular (LV) cavity at the time of reperfusion and then subcutaneously infused for 7 days (10 µg/day, n = 9). Control mice (n = 7) were treated with diluent (phosphate-buffered saline) only (bolus and infusion). LV pressure-volume measurements were performed after 28 days. LVEDP denotes LV end-diastolic pressure; LVESP, LV end-systolic pressure; LVEDV, LV end-diastolic volume; LVESV, LV end-systolic volume; LVEF, LV ejection fraction; dP/dt_{max}, maximum rate of pressure change in the left ventricle; dP/dt_{min}, minimum rate of pressure change in the left ventricle; τ, LV diastolic time constant. *$P < 0.05$, **$P < 0.01$, ***$P < 0.001$ vs. MI, control (two-sided independent samples *t* test).
