# Supplementary material for: Cysteine-rich with EGF-like domains 2 (CRELD2) is an endoplasmic reticulum stress-inducible angiogenic growth factor promoting ischemic heart repair
Source: Nat Cardiovasc Res. 2024 Jan 17;3(2):186–202. doi: 10.1038/s44161-023-00411-x (PMC11358006; doi:10.1038/s44161-023-00411-x)
Supplement: Supplementary file 2 — Reporting Summary [file 44161_2023_411_MOESM2_ESM.pdf]

nature portfolio

Corresponding author(s): Kai C. Wollert

Last updated by author(s): Jul 11, 2023

Reporting Summary

Nature Portfolio wishes to improve the reproducibility of the work that we publish. This form provides structure for consistency and transparency in reporting. For further information on Nature Portfolio policies, see our Editorial Policies and the Editorial Policy Checklist.

Statistics

For all statistical analyses, confirm that the following items are present in the figure legend, table legend, main text, or Methods section.

n/a	Confirmed	
☐	☒	The exact sample size (*n*) for each experimental group/condition, given as a discrete number and unit of measurement
☐	☒	A statement on whether measurements were taken from distinct samples or whether the same sample was measured repeatedly
☐	☒	The statistical test(s) used AND whether they are one- or two-sided<br>*Only common tests should be described solely by name; describe more complex techniques in the Methods section.*
☒	☐	A description of all covariates tested
☐	☒	A description of any assumptions or corrections, such as tests of normality and adjustment for multiple comparisons
☐	☒	A full description of the statistical parameters including central tendency (e.g. means) or other basic estimates (e.g. regression coefficient) AND variation (e.g. standard deviation) or associated estimates of uncertainty (e.g. confidence intervals)
☒	☐	For null hypothesis testing, the test statistic (e.g. *F*, *t*, *r*) with confidence intervals, effect sizes, degrees of freedom and *P* value noted<br>*Give P values as exact values whenever suitable.*
☒	☐	For Bayesian analysis, information on the choice of priors and Markov chain Monte Carlo settings
☒	☐	For hierarchical and complex designs, identification of the appropriate level for tests and full reporting of outcomes
☒	☐	Estimates of effect sizes (e.g. Cohen's *d*, Pearson's *r*), indicating how they were calculated

Our web collection on statistics for biologists contains articles on many of the points above.

Software and code

Policy information about availability of computer code

Data collection	10x Genomics cell ranger software 4.0.0
Data analysis	10x Genomics Loupe Browser 6.3.0, RStudio (2022.07.1+554 and R4.2.1), Ingenuity Pathway Analysis software (release 07/2023), Seurat 4.1.1, ggplot2 3.3.6, Scanpy 1.9.1, SignalP 4.1, TMHMM 2.0, GraphPad Prism 9.5, AxioVision 4.9, LabChart 7 Pro, MaxQuant 1.6.17, Perseus 1.6.14, ComplexHeatmap 2.18.0, and FlowJo 10.7.2

For manuscripts utilizing custom algorithms or software that are central to the research but not yet described in published literature, software must be made available to editors and reviewers. We strongly encourage code deposition in a community repository (e.g. GitHub). See the Nature Portfolio guidelines for submitting code & software for further information.

Data

Policy information about availability of data

All manuscripts must include a data availability statement. This statement should provide the following information, where applicable:

- Accession codes, unique identifiers, or web links for publicly available datasets
- A description of any restrictions on data availability
- For clinical datasets or third party data, please ensure that the statement adheres to our policy

We have deposited the scRNA-sequencing raw data in Gene Expression Omnibus (GEO; accession number, GSE198401). Human snRNA-seq data are publicly available at the Zenodo data archive (https://zenodo.org/record/6578047). We have deposited the phosphoproteomics dataset to the ProteomeXchange

consortium via the PRIDE partner repository (dataset identifier, PXD045013). All other data supporting the findings in this study are included in the main article and its associated files. Source data are provided with this paper.

Research involving human participants, their data, or biological material

Policy information about studies with human participants or human data. See also policy information about sex, gender (identity/presentation), and sexual orientation and race, ethnicity and racism.

Reporting on sex and gender	Age and sex are reported in the manuscript.
Reporting on race, ethnicity, or other socially relevant groupings	Race is reported as well.
Population characteristics	Left ventricular myocardial tissue samples were collected at autopsy from 12 patients (45–96 years; 6 men, 6 women; all white) who had died of an acute MI and 9 patients (56–97 years; 7 men, 2 women; all white) who had died from noncardiac causes. EDTA-treated plasma samples were obtained from 8 patients (46–71 years; 6 men, 2 women; all white) with acute myocardial infarction and from 7 apparently healthy individuals 7 (52–69 years; 5 men, 2 women; all white).
Recruitment	Autopsy samples were collected at the Department of Pathology at the VU University Medical Center in Amsterdam, Amsterdam, The Netherlands. Plasma samples from patients with acute myocardial infarction were collected at Hannover Medical School, Hannover, Germany. Plasma samples from apparently healthy individuals were collected at the Department of Medicine III at Heidelberg University, Heidelberg, Germany.
Ethics oversight	The Ethics Committees of the VU University Medical Center in Amsterdam, Hannover Medical School, and Heidelberg University approved our studies.

Note that full information on the approval of the study protocol must also be provided in the manuscript.

Field-specific reporting

Please select the one below that is the best fit for your research. If you are not sure, read the appropriate sections before making your selection.

☒ Life sciences ☐ Behavioural & social sciences ☐ Ecological, evolutionary & environmental sciences

For a reference copy of the document with all sections, see nature.com/documents/nr-reporting-summary-flat.pdf

Life sciences study design

All studies must disclose on these points even when the disclosure is negative.

Sample size	Sample sizes were chosen based on our previous experience with angiogenic growth factors driving infarct repair (Refs. 5-7).
Data exclusions	All mice surviving until the end of the experiment were included in the analyses. Positive and negative controls were included in all cell culture experiments. When these controls yielded the expected results, we included all samples from that experiment in the analyses.
Replication	All data presented in the manuscript were found to be reproducible in independent experiments. Whenever possible, results from individual mice and independent cell culture experiments are reported. The number of independent experiments (n) is reported in the Figure Legends.
Randomization	Mouse littermates were used in all experiments and randomly allocated to the experimental groups. In cell culture experiments using multi-titer plates, different treatment groups were randomly allocated to individual wells.
Blinding	For the animal experiments, investigators were blinded to group allocation during the experiment and when assessing outcome. For in vitro experiments, investigators were blinded when assessing functional read outs. Investigators were not blinded when assessing (phospho)protein or RNA expression data.

Reporting for specific materials, systems and methods

We require information from authors about some types of materials, experimental systems and methods used in many studies. Here, indicate whether each material, system or method listed is relevant to your study. If you are not sure if a list item applies to your research, read the appropriate section before selecting a response.

Materials & experimental systems

n/a	Involved in the study	
☐	☒	Antibodies
☐	☒	Eukaryotic cell lines
☒	☐	Palaeontology and archaeology
☐	☒	Animals and other organisms
☒	☐	Clinical data
☒	☐	Dual use research of concern
☒	☐	Plants

Methods

n/a	Involved in the study	
☒	☐	ChIP-seq
☐	☒	Flow cytometry
☒	☐	MRI-based neuroimaging

Antibodies

Antibodies used

Alpha-tubulin (clone EPR13478(B), Abcam, #ab185067, 1:3,500);
ATF6 (clone EPR22690-84, Abcam, #ab227830, 1:1,000);
P-eIF2alpha (Ser 51) (clone E90, Abcam, #ab32157, 1:1,000);
GAPDH (clone mAbcam 9484, Abcam, #ab9482, 1:1,000);
PERK (clone D11A8, Cell Signaling Technology, #5683, 1:1,000);
IRE1alpha (clone 14C10, Cell Signaling Technology, #3294, 1:1,000);
P-IRE1 alpha (S724) (polyclonal, Invitrogen, #PA1-16927, 1:1,000);
BIP (clone C50B12, Cell Signaling Technology, #3177, 1:1,000);
ATF4 (clone D4B8, Cell Signaling Technology, #11815, 1:1,000);
AKT (polyclonal, Cell Signaling Technology, #9272, 1:1,000);
P-AKT (S473) (polyclonal, Cell Signaling Technology, #9271, 1:1,000);
P AKT (T308) (polyclonal, Cell Signaling Technology, #9275, 1:1,000);
AMPKalpha1 (polyclonal, Cell Signaling Technology, #2795, 1:1,000);
AMPKalpha2 (polyclonal, Cell Signaling Technology, #2757, 1:1,000);
AMPKalpha (clone D5A2, Cell Signaling Technology, #5831, 1:1,000);
P-AMPKalpha (T172) (clone 40H9, Cell Signaling Technology, #2535, 1:1,000);
Caspase-3 (clone D3R6Y, Cell Signaling Technology, #14220, 1:1,000);
Caspase-9 (clone C9, Cell Signaling Technology, #9508, 1:1,000);
SMAD2/3 (clone D7G7, Cell Signaling Technology, #8685, 1:1,000);
P-SMAD2 (S465/S467) (clone138D4, Cell Signaling Technology, #3108, 1:1,000);
P-SMAD3 (S423/S425) (clone C25A9, Cell Signaling Technology, #9520, 1:1,000);
Human CRELD2 (polyclonal, MyBioSource, #MBS2527807, 1:1,000);
Mouse CRELD2 (polyclonal, R&D Systems, #AF3686, 1:1,000);
Human MANF (polyclonal, R&D Systems, #AF3748, 1:1,000);
COL1A1 (clone 3G3, Santa Cruz Biotechnology, #sc-293182, 1:1,000);
Neutralizing CRELD2 antibody (monoclonal, Bio-Rad);
GFP antibody (monoclonal, Bio-Rad);
CD31 (clone MEC 13.3, BD Biosciences, #553370, 1:100);
DyLight 550-labeled secondary antibody (polyclonal, Abcam, #ab98406, 1:200);
Ki67 (polyclonal, Abcam, #ab15580, 1:100);
Cy3-labeled secondary antibody (polyclonal, Jackson ImmunoResearch, #111-165-144, 1:200);
Alexa Fluor 488-conjugated secondary antibody (polyclonal, Invitrogen, #A-11055, 1:100);
Alpha-actinin (clone EA-53, Sigma-Aldrich, #A7732, 1:400);
TRITC-labeled secondary antibody (polyclonal, Sigma Aldrich, #T5393, 1:120);
Mouse CD16/CD32 (clone 2.4G2, BD Biosciences, #553141, 1:55);
CD45R/B220-PE (clone RA3-6B2, BD Biosciences, #553089, 1:500);
CD90.2/Thy-1.2-PE (clone 53-2.1, BD Biosciences, #553005, 1:2,500);
NK 1.1-PE (clone, PK136, BD Biosciences, #557391, 1:500);
CD49b/DX5-PE (clone DX5, BD Biosciences, #553858, 1:500);
Ly6G-PE (clone 1A8, BD Biosciences, #551461, 1:500);
I-Ab-FITC (clone AF6-120.1, BD Biosciences, #553551, 1:500);
CD11b-Alexa Fluor 700 (clone M1/70, BD Biosciences, #557960, 1:50);
CD45-Brilliant Violet 570 (clone 30-F11, BioLegend, #103136, 1:33);
F4/80-FITC (clone BM8, BioLegend, #123108, 1:33);
CD3-PE/Cy7 (clone 17A2, BioLegend, #100220, 1:33);
CD19-PerCP/Cy5.5 (clone 6D5, BioLegend, #115534, 1:33);
Ly6C-APC (clone 1G7.G10, Miltenyi Biotec, #130-123-796, 1:8);
CD11c-FITC (clone N418, Miltenyi Biotec, #130-122-939, 1:8).

Validation

Alpha-tubulin (clone EPR13478(B), Abcam, #ab185067)
Host species: Rabbit
Applications: IHC, WB
Species reactivity: Mouse, Rat, Human

ATF6 (clone EPR22690-84, Abcam, #ab227830)
Host species: Rabbit
Applications: WB, IHC, ChIP, IP
Species reactivity: Mouse, Human

P-eIF2alpha (Ser 51) (clone E90, Abcam, #ab32157)
Host species: Rabbit
Applications: WB, IHC, Dot blot
Species reactivity: Mouse, Rat, Human, Neurospora crassa

GAPDH (clone mAbcam 9484, Abcam, #ab9482)
Host species: Mouse
Application: WB
Species reactivity: Mouse, Rat, Human

PERK (clone D11A8, Cell Signaling Technology, #5683)
Host species: Rabbit
Applications: WB, IHC, IP
Species reactivity: Human

IRE1alpha (clone 14C10, Cell Signaling Technology, #3294)
Host species: Rabbit
Applications: WB, IP
Species reactivity: Human, Mouse, Rat

P-IRE1alpha (S724) (Polyclonal, Invitrogen, #PA1-16927)
Host species: Rabbit
Applications: WB, IHC, ICC/IF, IP
Species reactivity: Fruit fly, Goat, Human, Mammal, Mouse, Non-human primate, Pig, Rabbit, Rat

BIP (clone C50B12, Cell Signaling Technology, #3177)
Host species: Rabbit
Applications: WB, IHC, FC
Species reactivity: Human, Mouse

ATF4 (clone D4B8, Cell Signaling Technology, #11815)
Host species: Rabbit
Applications: WB, IP, IF, ChIP
Species reactivity: Human, Mouse, Rat

AKT (polyclonal, Cell Signaling Technology, #9272)
Host species: Rabbit
Applications: WB, IP, IF, FC
Species reactivity: Human, Mouse, Rat, Hamster, Monkey, Chicken, D. melanogaster, Bovine, Dog, Pig, Guinea Pig

P-AKT (S473) (polyclonal, Cell Signaling Technology, #9271)
Host species: Rabbit
Applications: WB, IP, IF, FC
Species reactivity: Human, Mouse, Rat, Hamster, Monkey, D. melanogaster, Bovine, Dog

P-AKT (T308) (polyclonal, Cell Signaling Technology, #9275)
Host species: Rabbit
Applications: WB, IP
Species reactivity: Human, Mouse, Rat

AMPKalpha1 (polyclonal, Cell Signaling Technology, #2795)
Host species: Rabbit
Application: WB
Species reactivity: Human, Monkey

AMPKalpha2 (polyclonal, Cell Signaling Technology, #2757)
Host species: Rabbit
Applications: WB, IP
Species reactivity: Human, Monkey

AMPKalpha (clone D5A2, Cell Signaling Technology, #5831)
Host species: Rabbit
Applications: WB, IP
Species reactivity: Human, Mouse, Rat, Monkey, Bovine

P-AMPKalpha (T172) (clone 40H9, Cell Signaling Technology, #2535)
Host species: Rabbit
Applications: WB, IHC, IP
Species reactivity: Human, Mouse, Rat, Hamster, Monkey, D. melanogaster, S. cerevisiae

Caspase-3 (clone D3R6Y, Cell Signaling Technology, #14220)
Host species: Rabbit
Applications: WB, IP
Species reactivity: Human, Mouse, Rat, Monkey

Caspase-9 (clone C9, Cell Signaling Technology, #9508)
Host species: Mouse
Applications: WB
Species reactivity: Human, Mouse, Rat, Hamster, Monkey

SMAD2/3 (clone D7G7, Cell Signaling Technology, #8685)
Host species: Rabbit
Applications: WB, IP, IF, FC
Species reactivity: Human, Mouse, Rat, Monkey

P-SMAD2 (S465/S467) (clone138D4, Cell Signaling Technology, #3108)
Host species: Rabbit
Applications: WB
Species reactivity: Human, Mouse, Rat, Mink

P-SMAD3 (S423/S425) (clone C25A9, Cell Signaling Technology, #9520)
Host species: Rabbit
Applications: WB, IP
Species reactivity: Human, Mouse, Rat

Human CRELD2 (polyclonal, MyBioSource, #MBS2527807)
Host species: Rabbit
Applications: WB, IHC
Species reactivity: Human

Mouse CRELD2 (polyclonal, R&D Systems, #AF3686)
Host species: Goat
Application: WB
Species reactivity: Mouse

Human MANF (polyclonal, R&D Systems, #AF3748)
Host species: Goat
Application: WB
Species reactivity: Human

COL1A1 (clone 3G3, Santa Cruz Biotechnology, #sc-293182)
Host species: Mouse
Applications: WB, IP, IF, IHC, ELISA
Species reactivity: Mouse, Rat, Human

Neutralizing CRELD2 antibody (monoclonal, Bio-Rad, custom-made)
Host species: Recombinant Mouse (Fab)/Human (Fc)
Application: Neutralization of CRELD2
Species reactivity: Mouse

GFP antibody (monoclonal, Bio-Rad, custom-made)
Host species: Recombinant Mouse (Fab)/Human (Fc)
Application: Negative control
Species reactivity: Aequorea Victoria

CD31 (clone MEC 13.3, BD Biosciences, #553370)
Host species: Rat
Applications: FC, IHC
Species reactivity: Mouse

DyLight 550-labeled secondary antibody (polyclonal, Abcam, #ab98406)
Host species: Goat
Applications: IHC, IF, FC
Target Species: Rat

Ki67 (polyclonal, Abcam, #ab15580)
Host species: Rabbit
Applications: IHC, IF
Species reactivity: Mouse, Human

Cy3-labeled secondary antibody (polyclonal, Jackson ImmunoResearch, #111-165-144)
Host species: Goat
Applications: IHC
Target Species: Rabbit

Alexa Fluor 488-conjugated secondary antibody (polyclonal, Invitrogen, #A-11055)
Host species: Donkey
Applications: IHC, IF, FC
Target Species: Goat

Alpha-actinin (clone EA-53, Sigma-Aldrich, #A7732)

Host species: Mouse
Applications: IF, IHC
Species reactivity: Mouse, Human, Rat, Cat, Fish, Rabbit, Sheep, Bovine, Goat, Canine, Pig, Hamster, Snake, Frog, Lizard, Chicken

TRITC-labeled secondary antibody (polyclonal, Sigma Aldrich, #T5393)
Host species: Goat
Applications: IF
Target Species: Mouse

Mouse CD16/CD32 (clone 2.4G2, BD Biosciences, #553141)
Host species: Rat
Applications: FC, IHC
Species reactivity: Mouse

CD45R/B220-PE (clone RA3-6B2, BD Biosciences, #553089)
Host species: Rat
Application: FC
Species reactivity: Mouse

CD90.2/Thy-1.2-PE (clone 53-2.1, BD Biosciences, #553005)
Host species: Rat
Application: FC
Species reactivity: Mouse

NK-1.1-PE (clone, PK136, BD Biosciences, #557391)
Host species: Mouse
Application: FC
Species reactivity: Mouse

CD49b/DX5-PE (clone DX5, BD Biosciences, #553858)
Host species: Rat
Application: FC
Species reactivity: Mouse

Ly6G-PE (clone 1A8, BD Biosciences, #551461)
Host species: Rat
Application: FC
Species reactivity: Mouse

I-Ab-FITC (clone AF6-120.1, BD Biosciences, #553551)
Host species: Mouse
Applications: FC
Species reactivity: Mouse

CD11b-Alexa Fluor 700 (clone M1/70, BD Biosciences, #557960)
Host species: Rat
Application: FC
Species reactivity: Mouse

CD45-Brilliant Violet 570 (clone 30-F11, BioLegend, #103136)
Host species: Rat
Application: FC
Species reactivity: Mouse

F4/80-FITC (clone BM8, BioLegend, #123108)
Host species: Rat
Application: FC
Species reactivity: Mouse

CD3-PE/Cy7 (clone 17A2, BioLegend, #100220)
Host species: Rat
Application: FC
Species reactivity: Mouse

CD19-PerCP/Cy5.5 (clone 6D5, BioLegend, #115534)
Host species: Rat
Application: FC
Species reactivity: Mouse

Ly6C-APC (clone 1G7.G10, Miltenyi Biotec, #130-123-796)
Host species: Rat
Application: FC
Species reactivity: Mouse

CD11c-FITC (clone N418, Miltenyi Biotec, #130-122-939)
Host species: Hamster

Applications: FC, IF, IHC
Species reactivity: Mouse

Eukaryotic cell lines

Policy information about cell lines and Sex and Gender in Research

Cell line source(s)	HEK-293T (American Type Culture Collection)
Authentication	Cells were directly obtained from the American Type Culture Collection (ATCC, #CRL-3216) and ATCC performed authentification using short tandem repeat profiling.
Mycoplasma contamination	HEK-293T cells were not tested for mycoplasma contamination.
Commonly misidentified lines (See ICLAC register)	We did not use a commonly misidentified cell line.

Animals and other research organisms

Policy information about studies involving animals; ARRIVE guidelines recommended for reporting animal research, and Sex and Gender in Research

Laboratory animals	Mice were housed in individually ventilated cages in a 12-hour light/dark cycle at stable room temperature (20-22°C) and humidity (45-50%). Food and water were provided ad libitum. We induced myocardial infarction (MI) in 8–10-week-old male mice. The following mouse strains were used: B6.129P2(SJL)-Creld2tm1.1Emass/J, Tg(TIE2GFP)287Sato/J, and C57BL/6J. Neonatal rat cardiomyocytes were isolated from Sprague-Dawley rats (1-3 days old, male and female).
Wild animals	Wild animals were not used in this study.
Reporting on sex	Male mice were used.
Field-collected samples	No field-collected samples were used in this study.
Ethics oversight	All animal procedures conformed to the guidelines from the EU directive 2010/63 on the protection of animals used for scientific purposes and were approved by the authorities in Hannover, Germany (Niedersächsisches Landesamt für Verbraucherschutz und Lebensmittelsicherheit).

Note that full information on the approval of the study protocol must also be provided in the manuscript.

Flow Cytometry

Plots

Confirm that:

- [x] The axis labels state the marker and fluorochrome used (e.g. CD4-FITC).
- [x] The axis scales are clearly visible. Include numbers along axes only for bottom left plot of group (a 'group' is an analysis of identical markers).
- [x] All plots are contour plots with outliers or pseudocolor plots.
- [x] A numerical value for number of cells or percentage (with statistics) is provided.

Methodology

Sample preparation	FACS and flow cytometry methods have previously been described (Refs. 5, 56, 57). Briefly, the infarct region of the left ventricle was digested with collagenase D (Roche), DNase I (Sigma-Aldrich), and dispase (Gibco) and processed with a gentleMACS dissociator (Miltenyi Biotec). Cell suspensions were filtered (40 µm cell strainer, Falcon), washed, and incubated for 5 min at 4 °C in PBS with 4% FBS, 2 mmol/L EDTA, and a purified mouse CD16/CD32 antibody (clone 2.4G2, mouse BD Fc Block, BD Biosciences, #553141, 1:55).
Instrument	FACSAria IIu and LSR II (Becton Dickinson)
Software	FlowJo 10.7.2
Cell population abundance	The relevant cell populations were quantified using TruCOUNT tubes (BD Biosciences) and the observed cell numbers per mg LV tissue were comparable to previous studies (Refs. 5, 6).
Gating strategy	Ly6Chigh monocytes were identified as CD45high CD11bhigh (CD45R/B220, CD90.2/Thy-1.2, NK 1.1, CD49b/DX5, Ly6G)low (CD11c, F4/80, I Ab)low Ly6Chigh; Ly6Clow monocytes or macrophages as CD45high CD11bhigh (CD45R/B220, CD90.2/Thy-1.2, NK 1.1, CD49b/DX5, Ly6G)low (CD11c, F4/80, I Ab)high/low Ly6Clow; neutrophils as CD45high CD11bhigh (CD45R/B220, CD90.2/Thy-1.2, NK 1.1, CD49b/DX5, Ly6G)high; T cells as CD45high CD11blow (CD45R/B220, CD90.2/Thy-1.2, NK 1.1,

CD49b/DX5, Ly6G)high CD3high CD19low; and B cells as CD45high CD11blow (CD45R/B220, CD90.2/Thy-1.2, NK 1.1, CD49b/DX5, Ly6G)high CD3low CD19high.

☒ Tick this box to confirm that a figure exemplifying the gating strategy is provided in the Supplementary Information.
