# Supplementary figures and images for: Cysteine-rich with EGF-like domains 2 (CRELD2) is an endoplasmic reticulum stress-inducible angiogenic growth factor promoting ischemic heart repair
Source: Nat Cardiovasc Res. 2024 Jan 17;3(2):186–202. doi: 10.1038/s44161-023-00411-x (PMC11358006; doi:10.1038/s44161-023-00411-x)

Source data Figure 2 - uncropped blots

Figure 2b

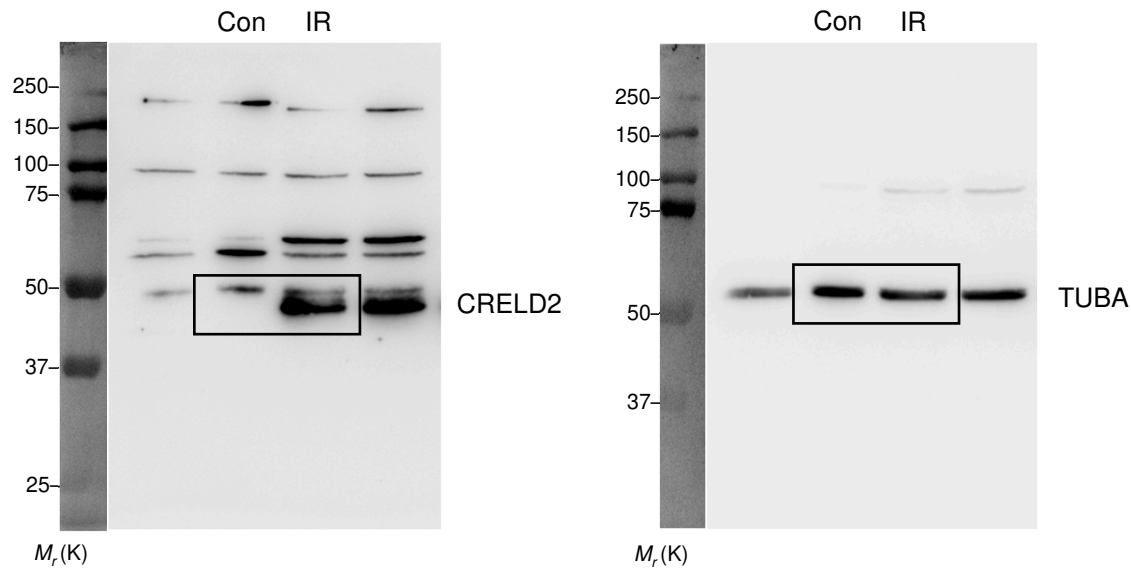

Figure 2c

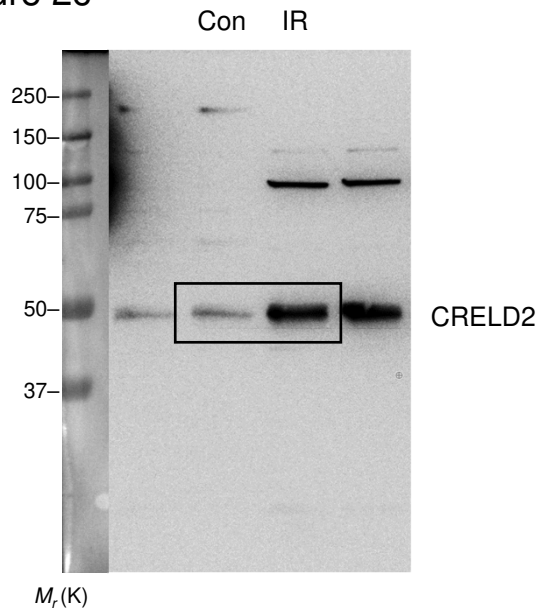

Figure 2e

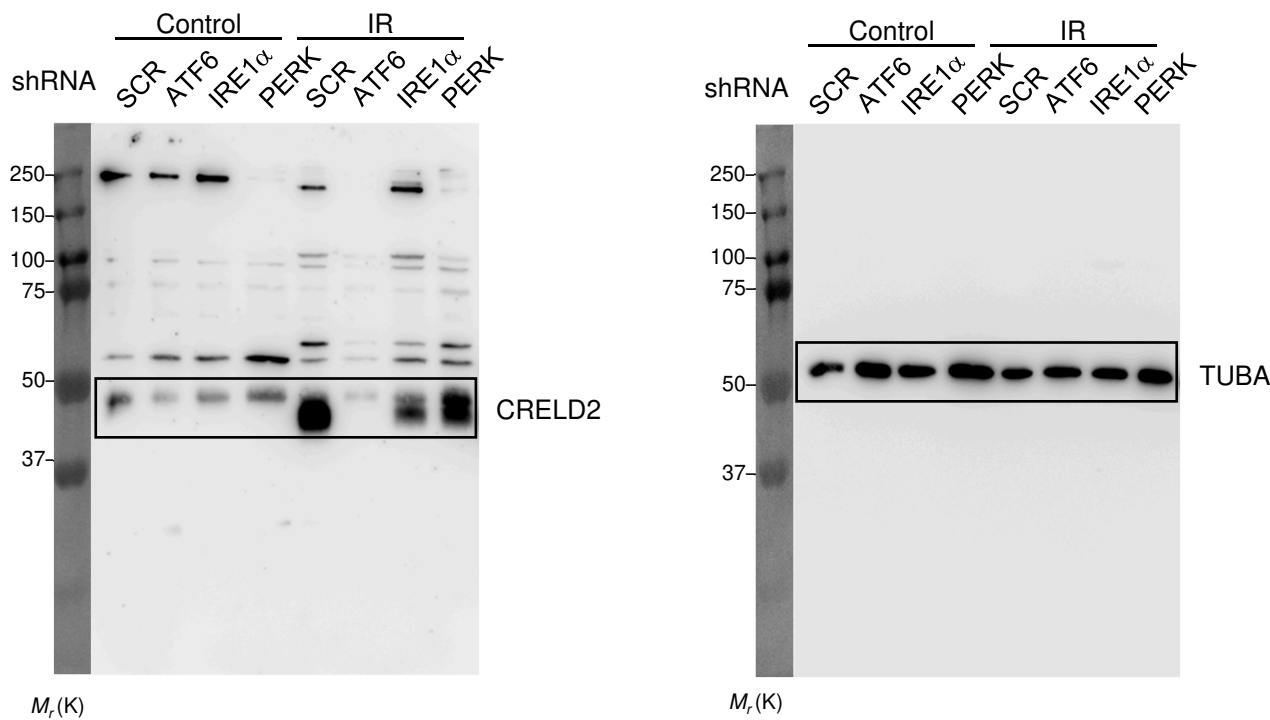

Figure 2f

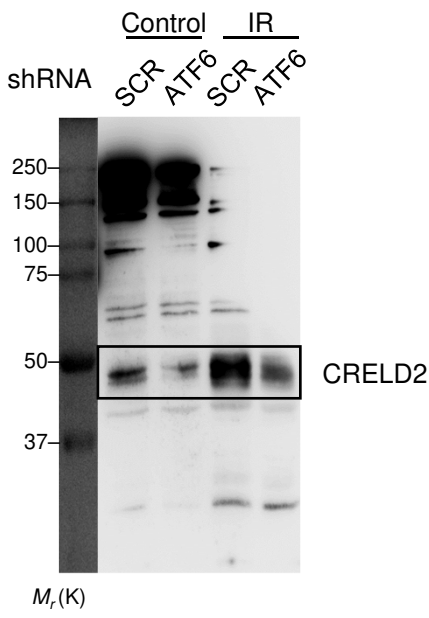

Figure 2h

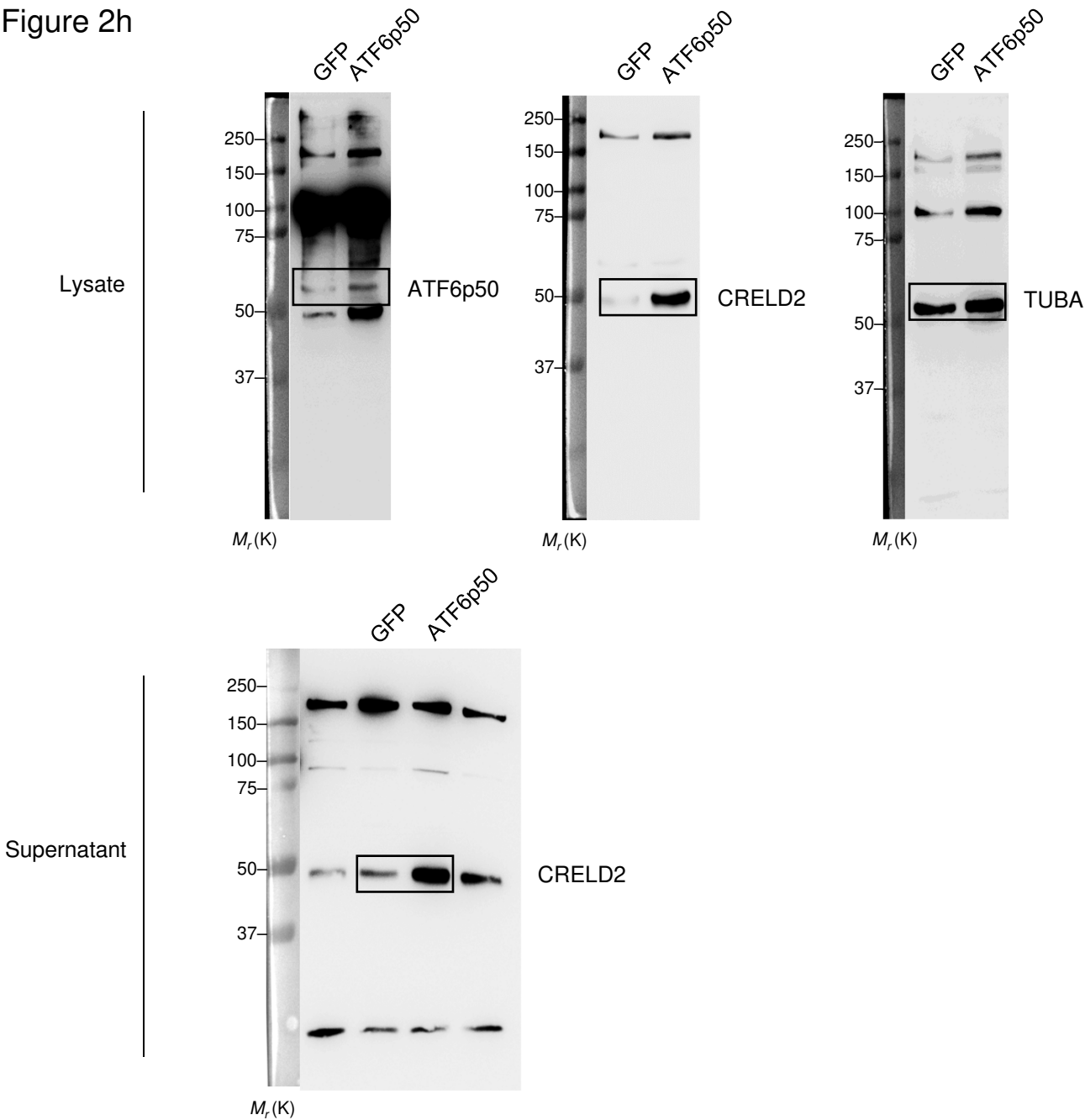

Supplement: Supplementary file 7 — Unprocessed western blots and gels. [file 44161_2023_411_MOESM7_ESM.pdf]

Source data Figure 3 - uncropped blots

Figure 3a

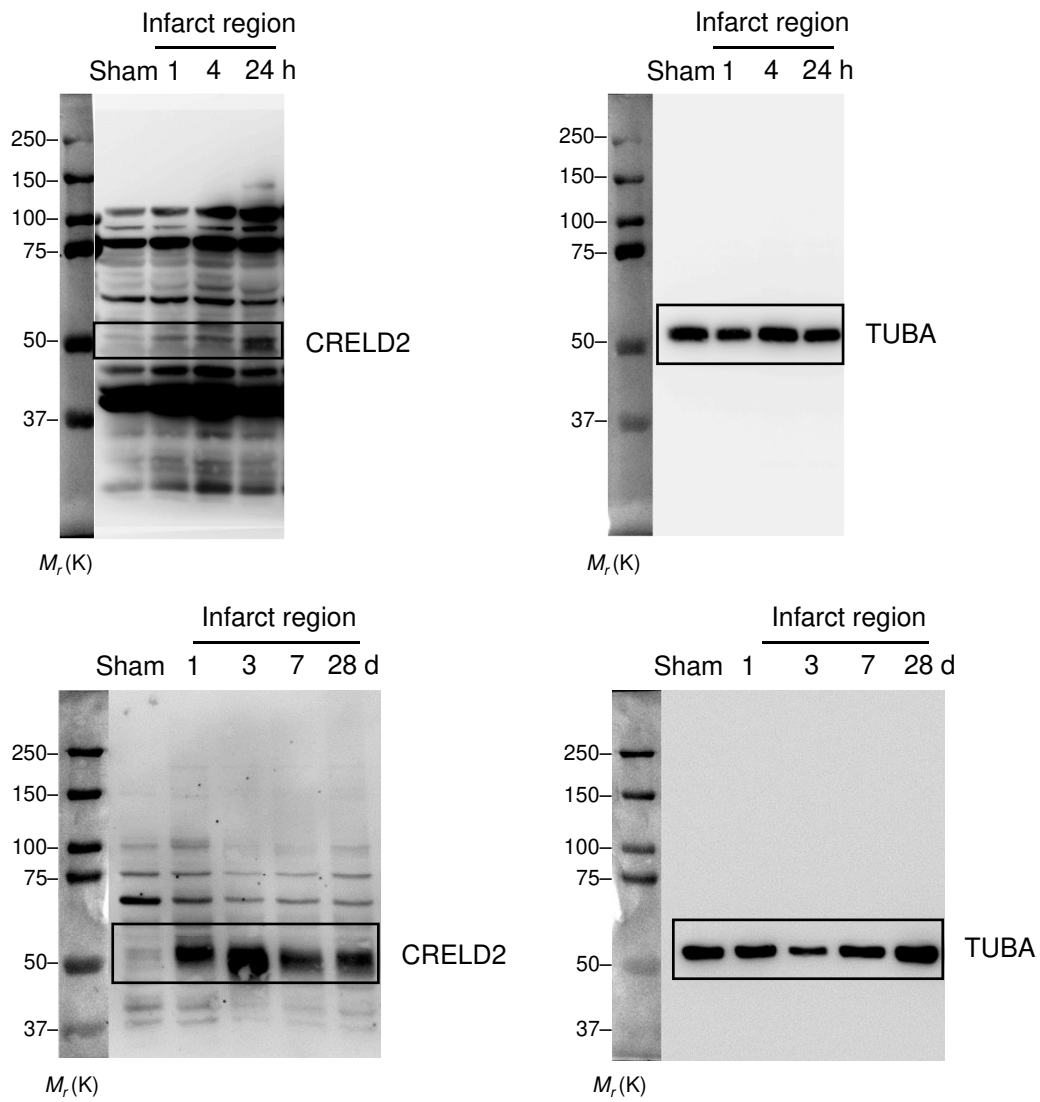

Figure 3c

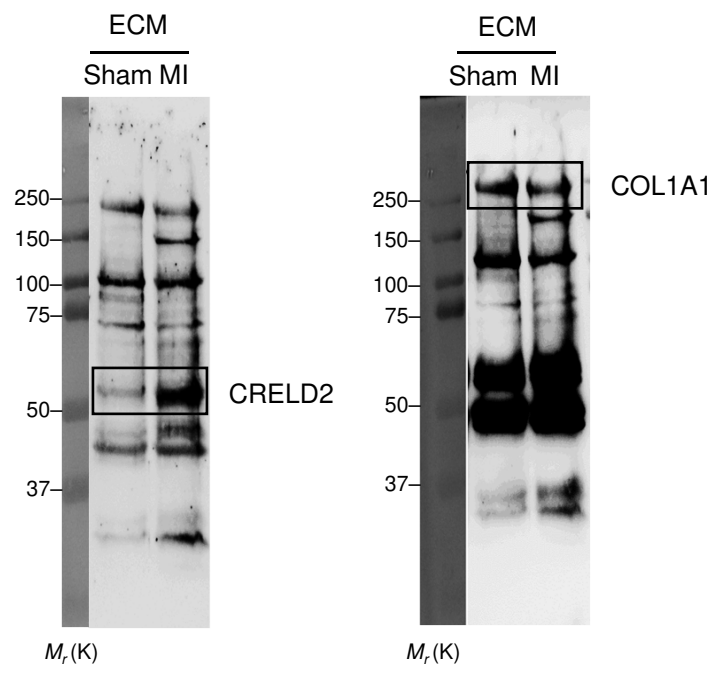

Figure 3g

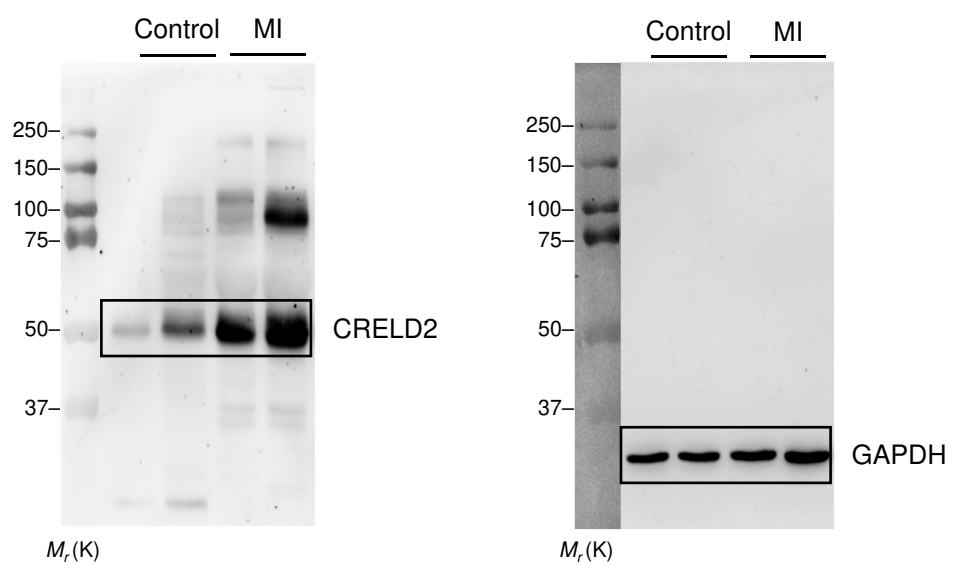

Supplement: Supplementary file 9 — Unprocessed western blots. [file 44161_2023_411_MOESM9_ESM.pdf]

## Figure 5a

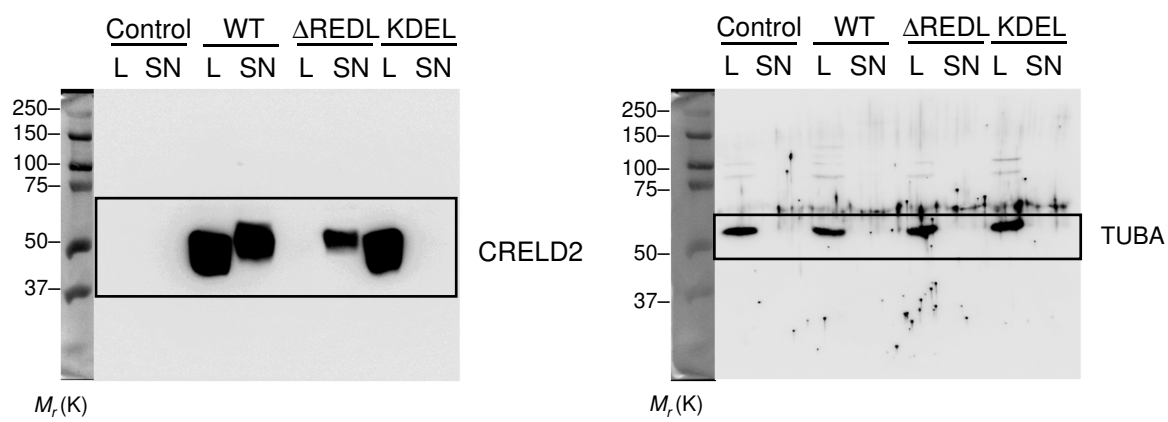

Supplement: Supplementary file 12 — Unprocessed western blots. [file 44161_2023_411_MOESM12_ESM.pdf]

Source data Figure 6 - uncropped blots

Figure 6c

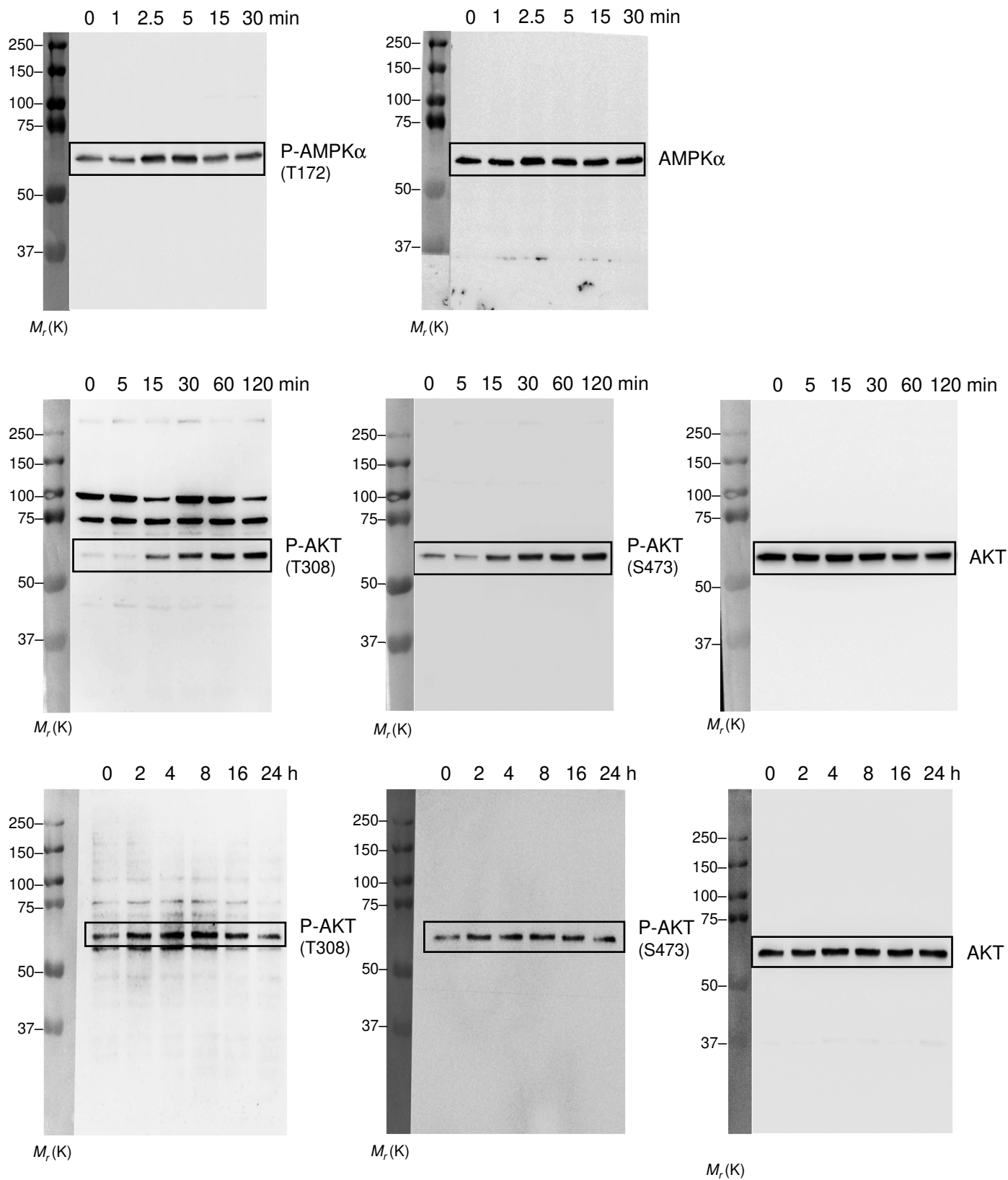

Figure 6g

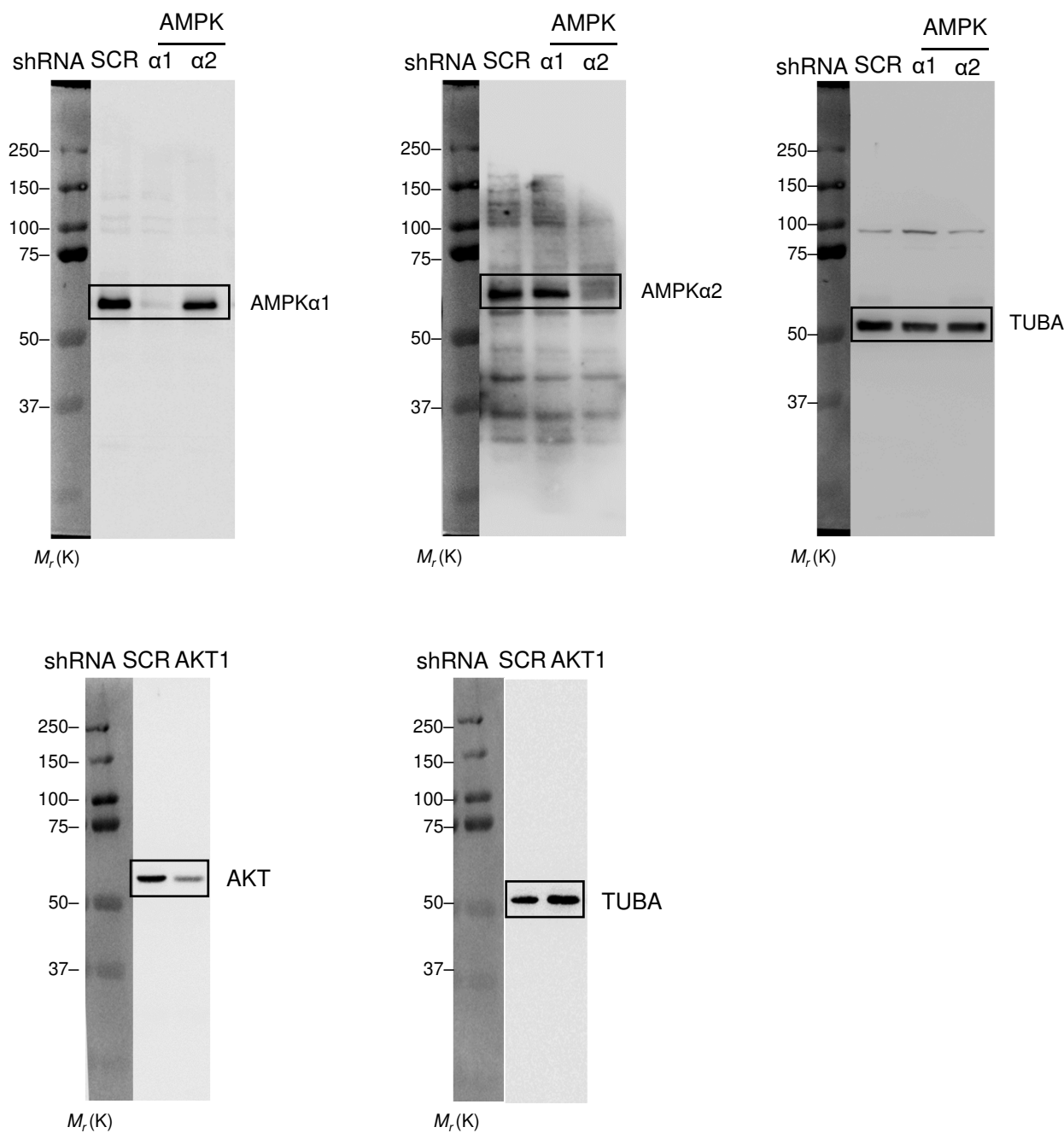

Supplement: Supplementary file 14 — Unprocessed western blots. [file 44161_2023_411_MOESM14_ESM.pdf]

Source data Figure 8 - uncropped blots

Figure 8e

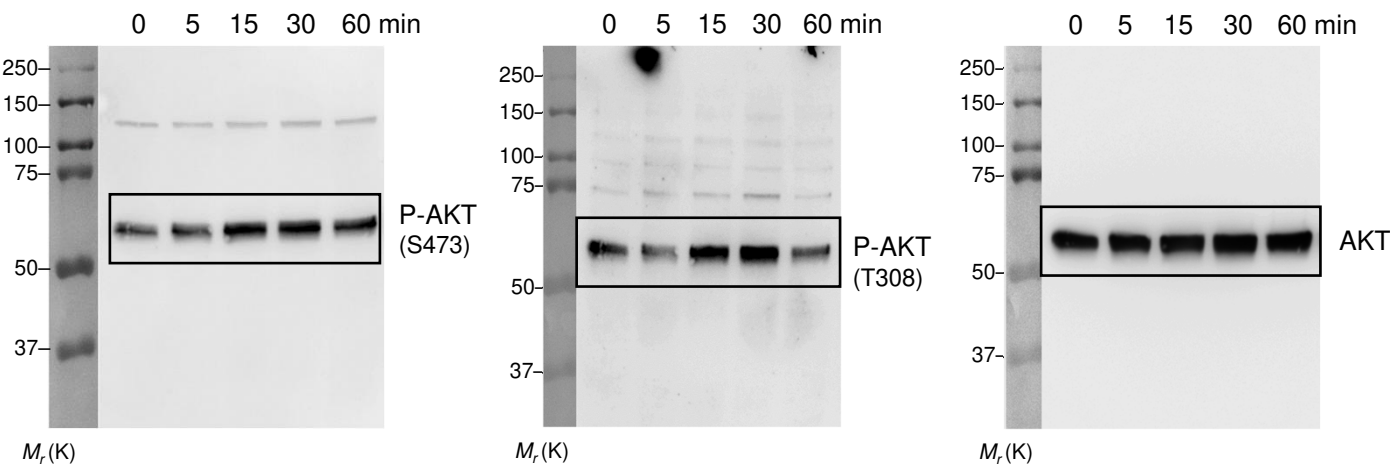

Figure 8g

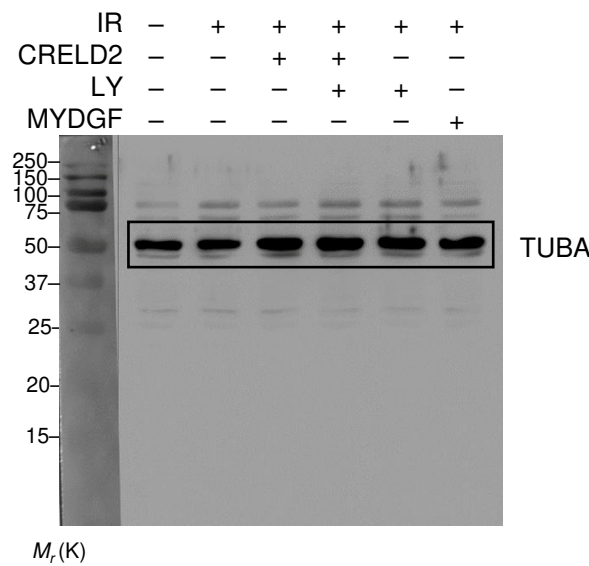

Supplement: Supplementary file 17 — Unprocessed western blots. [file 44161_2023_411_MOESM17_ESM.pdf]

Source data ED Figure 5 - uncropped blots

ED Figure 5e

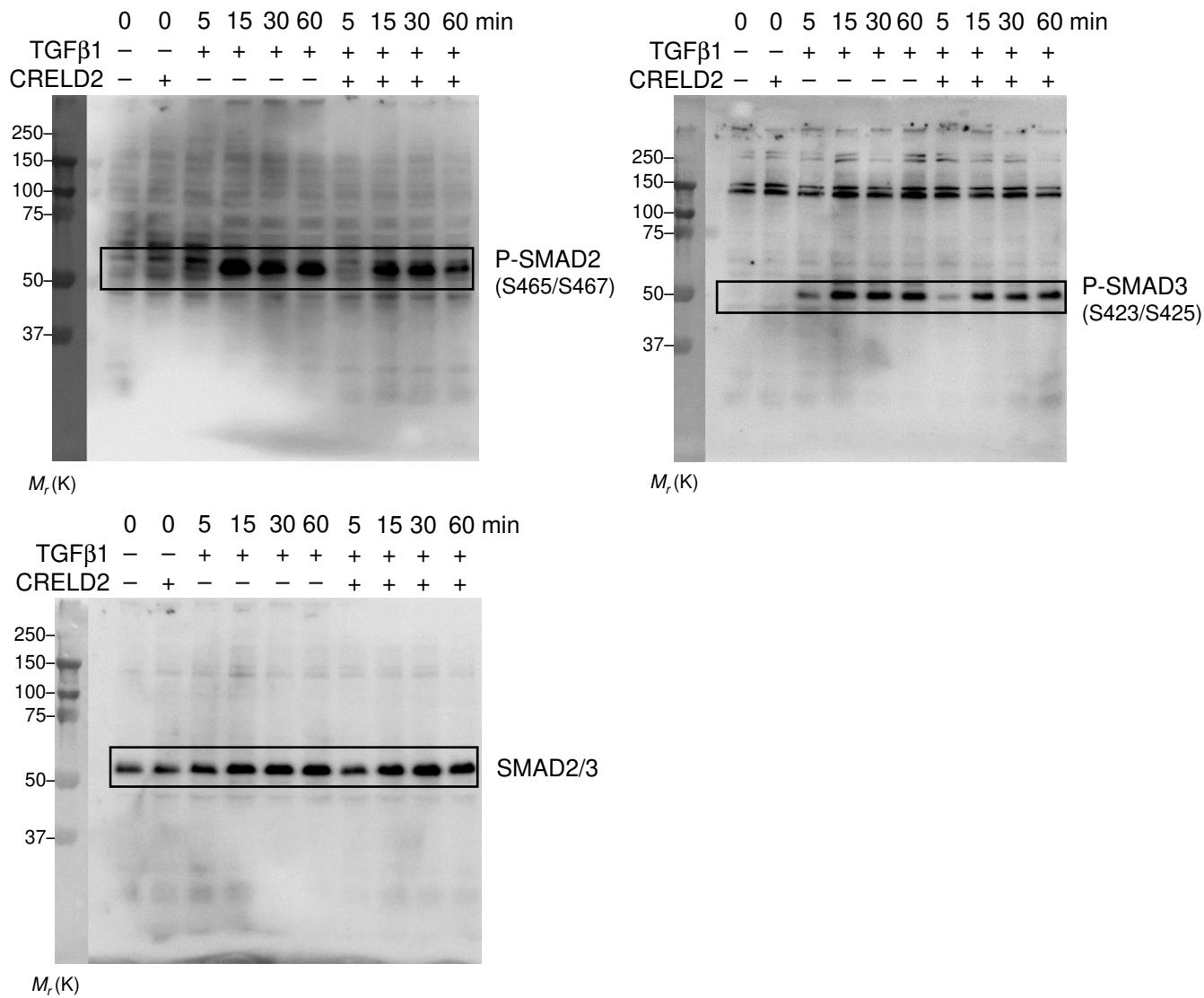

Supplement: Supplementary file 24 — Unprocessed western blots. [file 44161_2023_411_MOESM24_ESM.pdf]
