# Supplementary material for: Cysteine-rich with EGF-like domains 2 (CRELD2) is an endoplasmic reticulum stress-inducible angiogenic growth factor promoting ischemic heart repair
Source: Nat Cardiovasc Res. 2024 Jan 17;3(2):186–202. doi: 10.1038/s44161-023-00411-x (PMC11358006; doi:10.1038/s44161-023-00411-x)
Supplement: Supplementary file 19 — Unprocessed western blots and gels. [file 44161_2023_411_MOESM19_ESM.pdf]

Source data ED Figure 1 - uncropped blots

ED Figure 1a

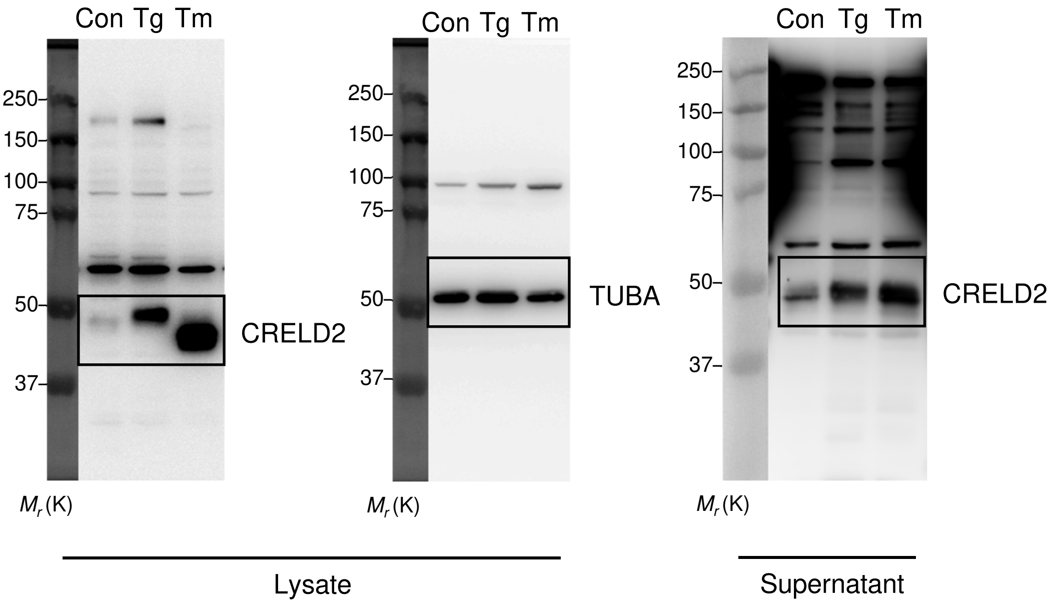

ED Figure 1b

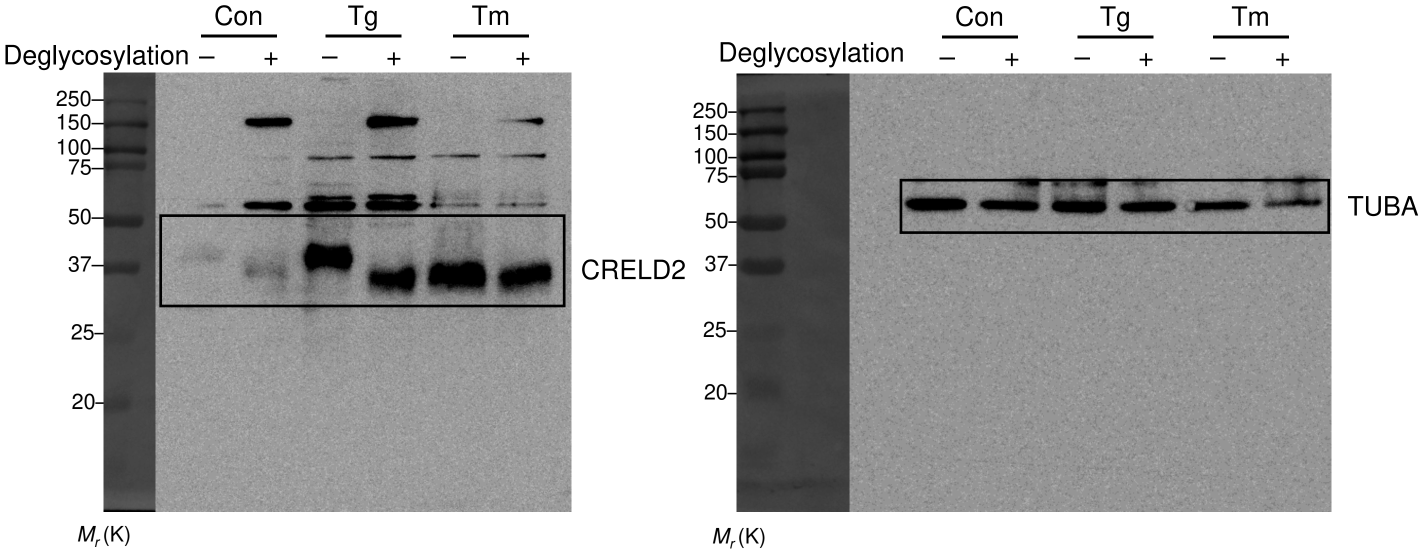

ED Figure 1c

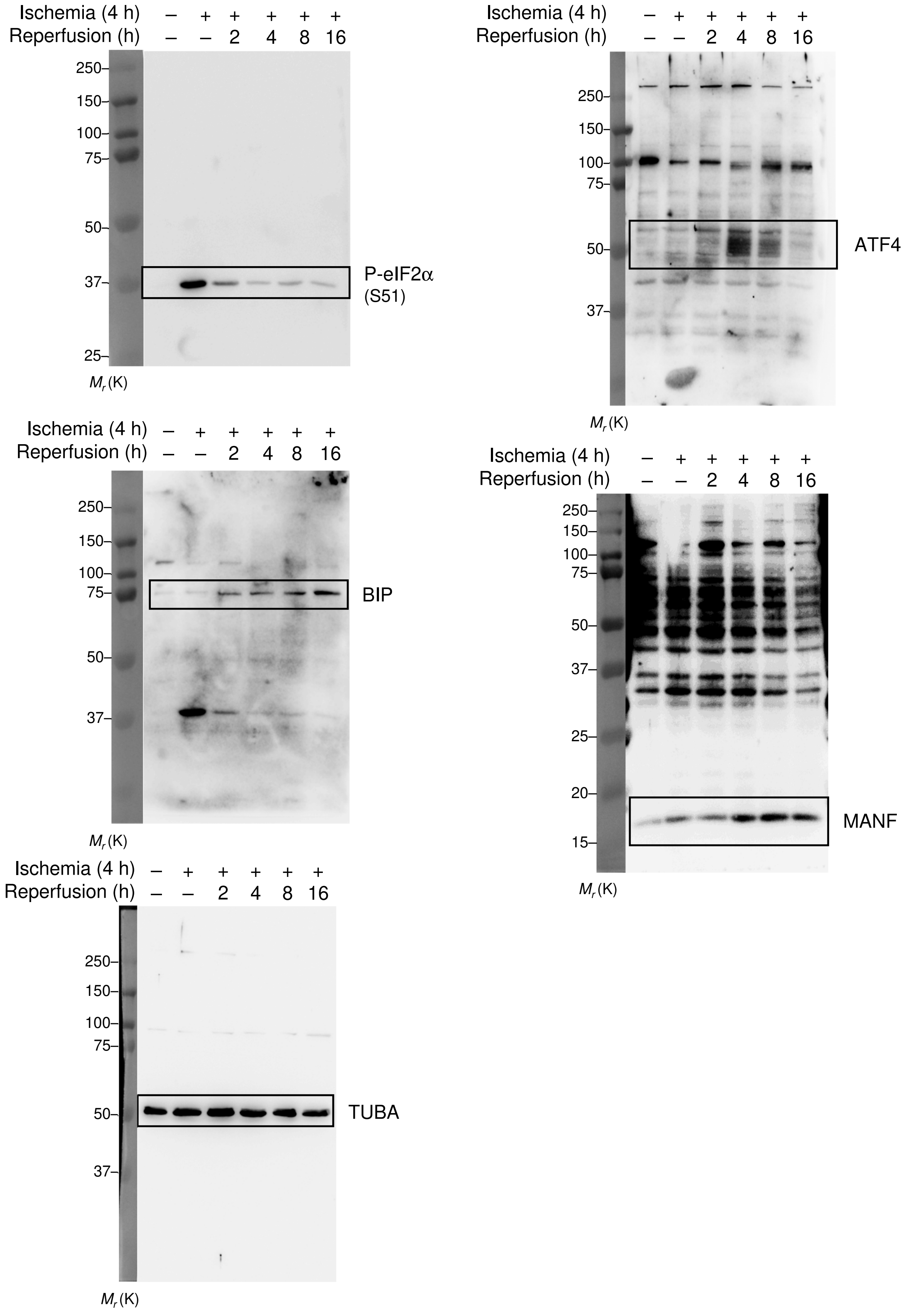
Ischemia (4 h) – + + + + +
Reperfusion (h) – – 2 4 8 16
250–
150–
100–
75–
50–
37–
25–
P-eIF2α
(S51)
M_r (K)
Ischemia (4 h) – + + + + +
Reperfusion (h) – – 2 4 8 16
250–
150–
100–
75–
50–
37–
ATF4
M_r (K)
Ischemia (4 h) – + + + + +
Reperfusion (h) – – 2 4 8 16
250–
150–
100–
75–
50–
37–
BIP
M_r (K)
Ischemia (4 h) – + + + + +
Reperfusion (h) – – 2 4 8 16
250–
150–
100–
75–
50–
37–
25–
20–
15–
MANF
M_r (K)
Ischemia (4 h) – + + + + +
Reperfusion (h) – – 2 4 8 16
250–
150–
100–
75–
50–
37–
TUBA
M_r (K)

ED Figure 1d

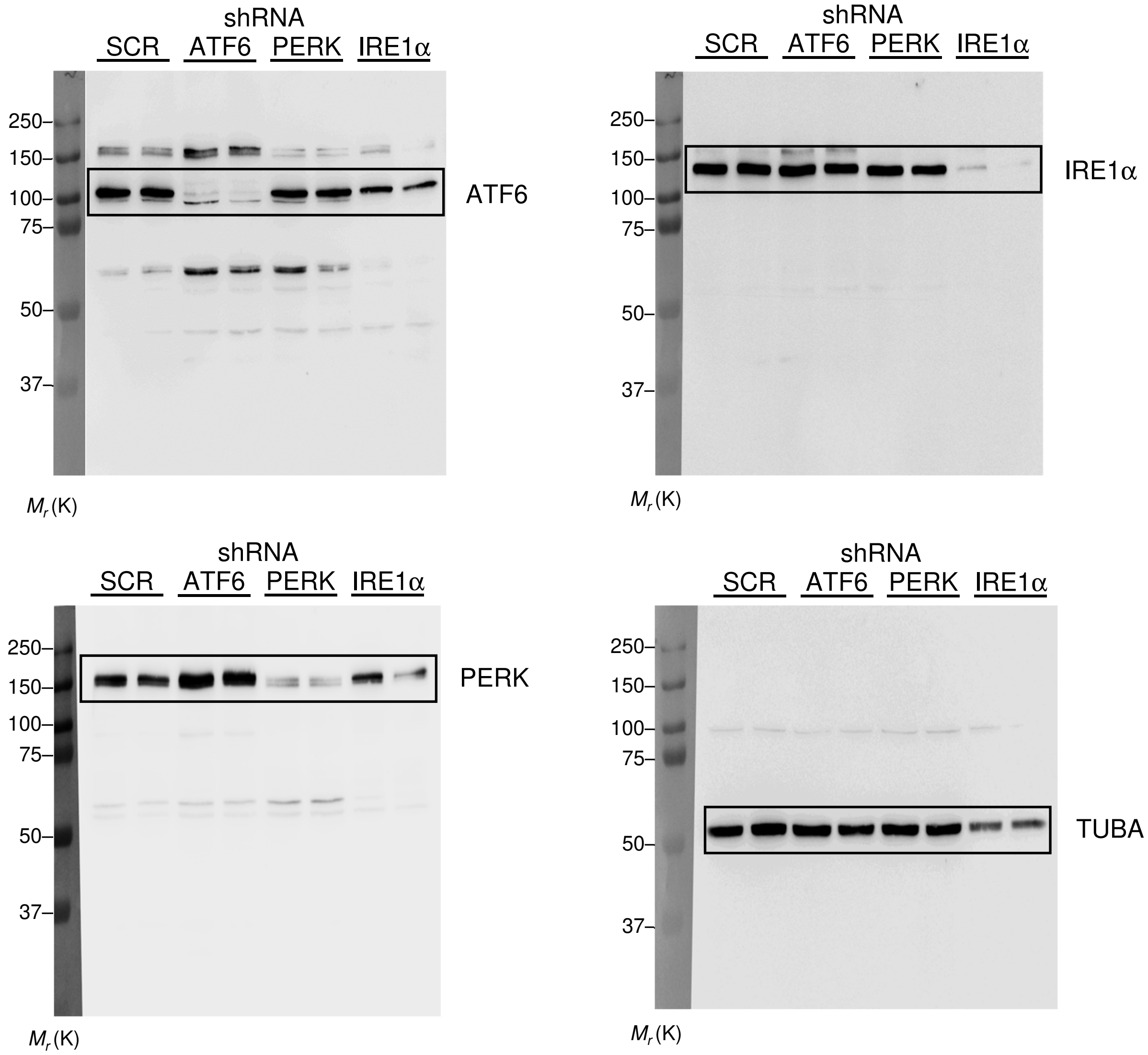
shRNA
SCR
ATF6
PERK
IRE1α
250–
150–
100–
75–
50–
37–
ATF6
M_r (K)
shRNA
SCR
ATF6
PERK
IRE1α
250–
150–
100–
75–
50–
37–
IRE1α
M_r (K)
shRNA
SCR
ATF6
PERK
IRE1α
250–
150–
100–
75–
50–
37–
PERK
M_r (K)
shRNA
SCR
ATF6
PERK
IRE1α
250–
150–
100–
75–
50–
37–
TUBA
M_r (K)

ED Figure 1f

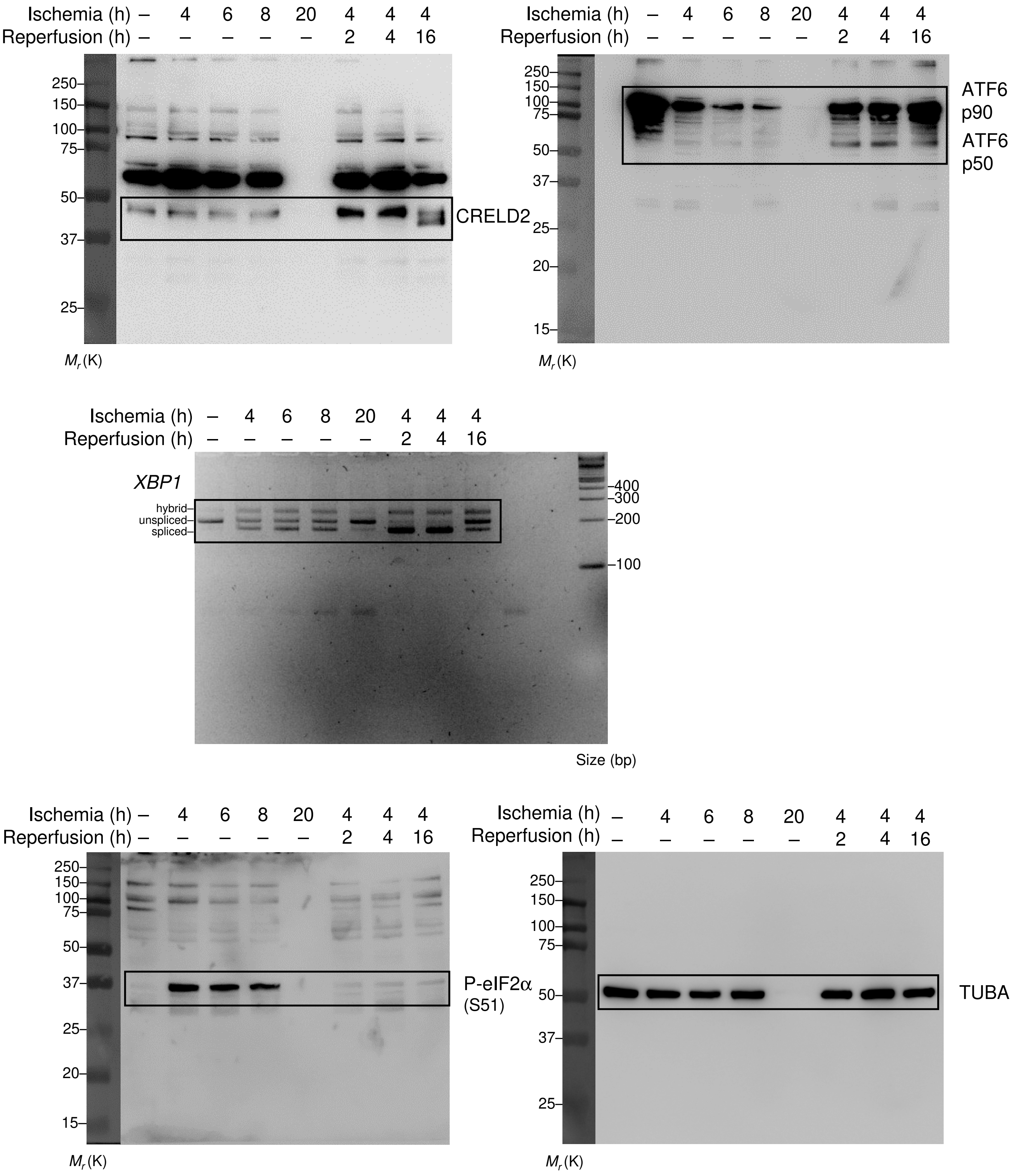
Ischemia (h) – 4 6 8 20 4 4 4
Reperfusion (h) – – – – – 2 4 16
250 150 100 75 50 37 25
CRELD2
Mr (K)
Ischemia (h) – 4 6 8 20 4 4 4
Reperfusion (h) – – – – – 2 4 16
250 150 100 75 50 37 25 20 15
ATF6 p90
ATF6 p50
Mr (K)
Ischemia (h) – 4 6 8 20 4 4 4
Reperfusion (h) – – – – – 2 4 16
XBP1
hybrid
unspliced
spliced
400 300 200 100
Size (bp)
Ischemia (h) – 4 6 8 20 4 4 4
Reperfusion (h) – – – – – 2 4 16
250 150 100 75 50 37 25 20 15
P-eIF2α (S51)
Mr (K)
Ischemia (h) – 4 6 8 20 4 4 4
Reperfusion (h) – – – – – 2 4 16
250 150 100 75 50 37 25
TUBA
Mr (K)
